# Helical foldamers replicating membrane-spanning gramicidin a with pH responsiveness and ultrafast potassium permeability†

**DOI:** 10.1039/d5sc01362c

**Published:** 2025-07-22

**Authors:** Jun Tian, Lei Zhang, Ze Lin, Shizhong Mao, Zeyuan Dong

**Affiliations:** a State Key Laboratory of Supramolecular Structure and Materials, College of Chemistry, Jilin University Changchun 130012 China zdong@jlu.edu.cn; b Center for Supramolecular Chemical Biology, Jilin University Changchun 130012 China

## Abstract

Structural simulation of natural ion channels remains a challenging topic. To fabricate artificial ion channels structurally resembling natural gramicidin A (gA), we prepared a type of precise hollow helical molecular channel by means of a modular synthesis strategy. Helical molecules are able to form 2.9 nm membrane-spanning channels through dimeric π-stacking assembly and efficiently accelerate ion transmembrane transport, with ultrahigh transport activity of up to 28 nM. Among these molecular channels with transmembrane structures similar to gA, one of them significantly exceeds natural gA for potassium ion transport, while another one exhibits the same proton transport activity as natural gA under identical conditions. Moreover, we found that the positive charges near the entrance of the channel reduce the potassium transport rate of the channel but significantly promote proton transport. In addition, a molecular channel with terminal amine groups shows pH-regulated ion transport function. This is the first example of structural replication of natural gA, in which helically folded molecules with assembled dimeric structure yield fantastic ion transport properties.

## Abstract 2

This study represents structural mimicry of natural gramicidin A through helix-based dimeric assembly, featuring efficient ion transport, even superior K^+^ transport over gramicidin A, and pH responsiveness.

## Graphical abstract

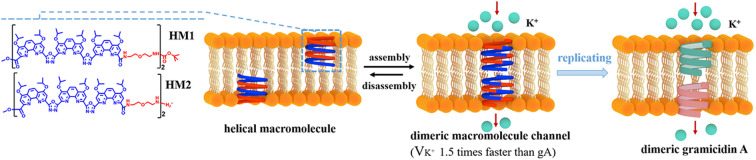

## Introduction

Natural ion channels regulate cellular ion permeation to maintain normal life activity. The dysfunctions of ion channels usually result in channelopathies.^1^ The function of compensation of mutated channel proteins has proven especially promising to treat ion blockade diseases,^2^ which opens up the possibility of using artificial channels with ion transport functions in the development of channelopathy drugs. Although some protein structures of ion channels have been elucidated by X-ray crystal diffraction or electron microscopy,^3^ the complexity of channel structures seriously restricts the design of chemical counterparts. Moreover, natural ion channels typically possess fascinating properties such as high ion selectivity, fast ion transport, and responsiveness to external stimuli (*e.g.*, voltage,^4^ binding ligands,^5^ temperature,^6^ light,^7^ pH,^8^ and mechanical stress^9^), making functional mimicry attractive but difficult. Notably, a relatively simple polypeptide, gramicidin A (gA) consisting of an alternating sequence of eight l- and seven d-amino acids,^10^ has become an achievable objective that can be simulated structurally at present. As is known, natural channel gA is capable of inserting into the hydrophobic region of the lipid membranes in a head-to-head manner and transporting various monovalent cations through a dimer-based channel.^11^ The polypeptide gA with a pore aperture of 4.0 Å shows high transport activity and ion selectivity with the order of H^+^ > NH_4_^+^ > Cs^+^ > Rb^+^ > K^+^ > Na^+^ > Li^+^.^12^ Owing to the unique structure and function of gA, it is important to chemically modify the structure of gA and even to functionally simulate gA.^13^ For example, semisynthetic gA derivatives had been reported *via* the decoration of charged groups into the C-terminus of gA, in which the resulting transmembrane channels exhibit rectified conductance behavior useful in biosensors.^14^ Hou and coworkers reported a type of unimolecular peptide channel designed by using the β-helical conformation of gA, and the channels can pump K^+^ across the lipid bilayer and cause a membrane potential.^15^ In addition, Li and coworkers developed a series of aromatic hydrazide helical oligomers and polymers, and these channels show significant ion selectivity and high transport efficiency.^16^ Barboiu *et al.* reported a self-assembled channel from a simple triazole to simulate the function of gA and thus represented an artificial primitive gA mimic.^17^ However, the need for stepwise organic synthesis makes accurate structural simulation of gA difficult to achieve.

**Fig. 1:**
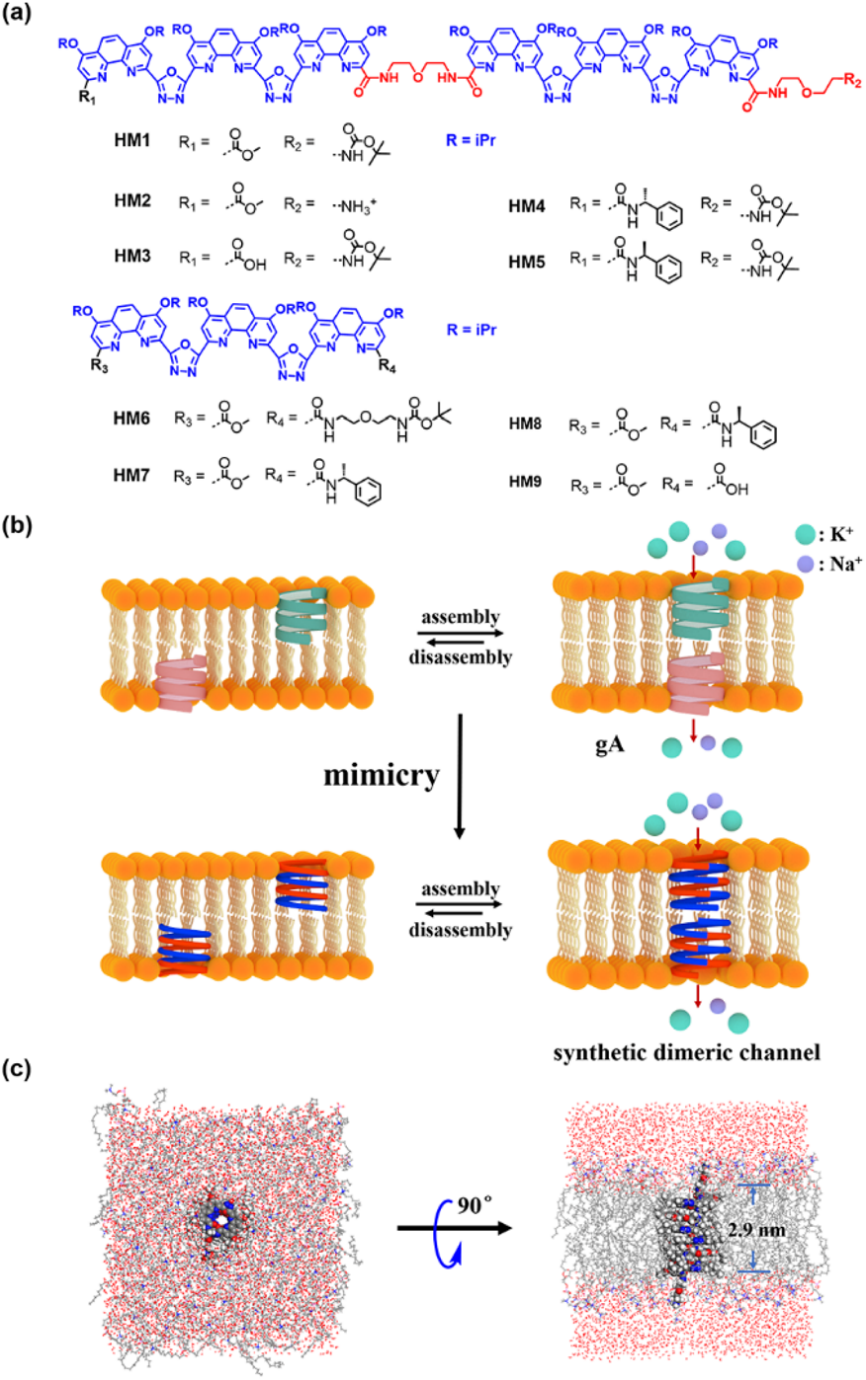
(a) Molecular formula of HM1-HM9. (b) Schematic representation of dimeric channels of gA and HM2 in the lipid membranes by means of structural mimicry. (c) The top and side views of the dimeric channel of HM2 in DOPC membranes in the all-atom MD simulation.

Currently, the state of the art of artificial ion channels thoroughly concentrates on simulating the properties and function of natural ion channels by using various structures, including multicomponent self-assembled and polymeric skeletons.^18^ However, research on replicating the structure of natural channels, even to mimic the simple channel gA, has not been achieved yet. In fact, the synthesis of helically folded porous helical macromolecules with precise chemical structures is very difficult, although several long helical polymers have been reported so far.^19^ Herein, we adopt a modular synthesis strategy to prepare precise hollow helical molecules for structural replication of natural gA. The helically folded molecules (HM1-HM5, Fig. 1a) with different end groups were thus designed and synthesized by using *o*-phenanthroline-oxadiazole-based pentamers as modular components bridged with oxybis(ethan-1-amine) through efficient stepwise preparation. MS simulation shows that the length of an individual gA-like helical molecule is 1.3∼1.4 nm. Additionally, two gA-like helical molecules can form a channel across the membrane by π–π interaction. Therefore, the height of dimerized gA-like helical molecules on phospholipid membranes is the addition of the heights of two gA-like molecules and the π–π interaction distance. These helically folded molecules can form supramolecular dimers with a length of 2.9 nm through intermolecular π–π interactions (Fig. 1b), which is almost consistent with the channel length of dimeric gA, and can effectively span the lipid membranes (Fig. 1c). Notably, HM1-HM5 are all capable of transporting monovalent cations (*e.g.*, H^+^, Na^+^, and K^+^) efficiently across the lipid membranes. Meanwhile, helical molecular channel HM1 exhibits extremely high transport activity for potassium ions, with a half-maximal effect concentration (EC_50_) of 28 nM. The Hill coefficient was found to be 0.6 (*n* < 1), suggesting that the gA-like HM1 already self-assembles into a supramolecular channel. To our surprise, the ability of helical molecular channel HM1 to transport potassium ions is 1.5 times stronger than that of natural gA, which should be attributed to precise structural simulation. Moreover, we found that positive charges near the channel entrance seriously hinder potassium ion transport but significantly promote proton transport, thereby achieving proton transport activity of helical molecular channel HM2 as high as that of natural gA. In addition, protonation and deprotonation of amine groups near the channel entrance allow for pH regulation of transport activity of helical molecular channels, thus for the first time constructing structurally precise and pH-adjusted artificial ion channels.

## Results and discussion

### Design and synthesis of molecular channels HM1-HM5

In the field of biomimetic ion channels, helical molecular channels with precise chemical structures have long been pursued as the required structural models.^19*d*,20^ However, the synthesis of helical molecular channels definitely faces difficulties, and currently there are only a few types of porous helical polymers prepared through polymerization methods.^1*b*,16,21^ In addition, several aromatic amide helical molecules with atom-precise structures prepared through rigorous stepwise synthesis have been reported recently,^19*a*,*b*,*d*^ but they do not have defined porous structures for transport function. To solve the synthetic difficulty, we utilize a modular synthesis strategy to prepare precise hollow helical molecules. In particular, the *o*-phenanthroline-oxadiazole-based pentamer, whose crystal structure has been well studied,^22^ was used as a modular component, which can be bridged with oxybis(ethan-1-amine) to synthesize the helical molecular channels HM1-HM5. The oxybis(ethan-1-amine)-bridged aromatic amide helices have been well investigated,^23^ which is helpful for the structural design of precise hollow helical molecules. Consequently, helical molecules with different end groups were synthesized and fully characterized (Fig. S1–S38†). Meanwhile, helical molecule HM2 has a positively charged amine group at the terminal position, and HM3 has a carboxyl group at the terminal position. In order to observe the helicity, HM4 and HM5 with opposite chiral groups have also been prepared. At the same time, several half-shortened helices, HM6-HM9, with similar structures were synthesized for comparison.

**Fig. 2:**
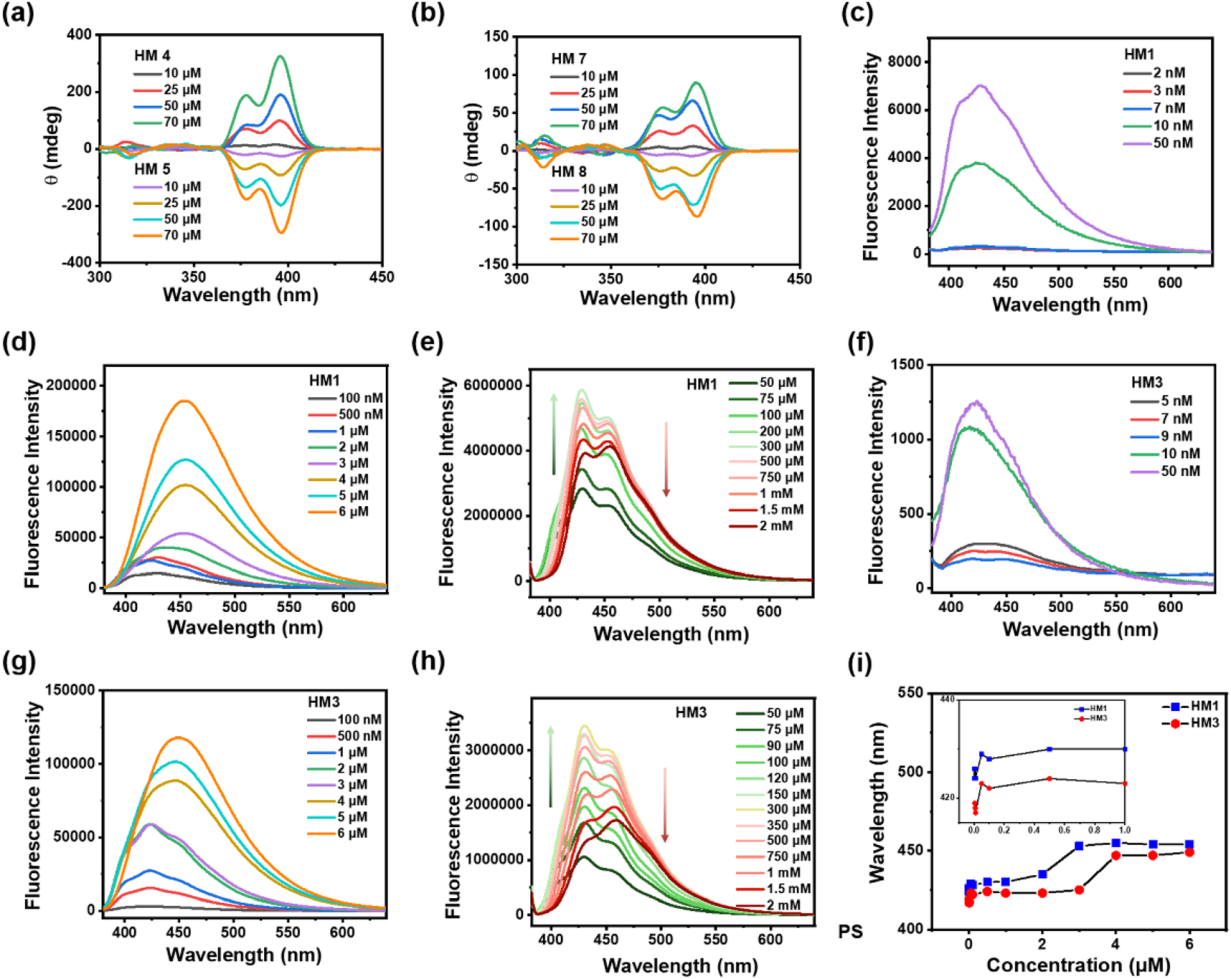
(a) The CD spectra of HM4 and HM5 at different concentrations in chloroform. (b) The CD spectra of HM7 and HM8 at different concentrations in chloroform. (c)–(e) Fluorescence titration of HM1 (excitation wavelength: 370 nm). (f)–(h) Fluorescence titration of HM3 (excitation wavelength: 370 nm). (i) Quantified analysis of the relationship between the maximum emission wavelength and the concentration of HM1 and HM3.

To confirm the helical conformation of molecular channels, chiral HM4 and HM5 with covalently modified (*S*)-1-phenylethylamine and (*R*)-1-phenylethylamine, respectively, were studied by circular dichroism (CD) spectroscopy. As expected, the racemic helices HM1 and HM9 did not show any CD signals (Fig. S39†). However, chirality-induced HM4 showed explicit positive CD signals at 395 nm with concentration-dependent optical activity, while HM5 exhibited completely mirrored optical activity (Fig. 2a). The short chiral HM7 and HM8 exhibited opposite CD signals in chloroform (Fig. 2b), and their CD characteristic peaks are similar to those of helical molecules HM4 and HM5. This is reasonable because helical molecule channels are composed of two short helices bridged with an oxybis(ethan-1-amine) linker. Unexpectedly, the CD intensities of helical molecules HM4 and HM5 at 395 nm were found to be at least 2.5-fold higher than those of half-shortened HM7 and HM8 at the same concentration (Fig. S40, S41, and Table S1†), suggesting that the intramolecular interactions between two helical components within the helical molecular structure enable the helicity amplification (1 + 1 > 2). These observations suggest that channels HM1-HM5 have complete helical structures in solution.

### Dimeric π-stacking assembly of HM1

In order to observe intramolecular and dimeric π-stacking assembly of helical molecular channels, concentration-dependent fluorescence spectroscopy of HM1 was carried out. When the concentration increases from 2 nM to 6 μM, the maximum emission wavelength (*λ*_em_) of HM1 shows a significant redshift from 425 nm to 453 nm in the fluorescence spectra (Fig. 2c and d), suggesting that the π-stacking assembly of HM1 takes place in solution. Importantly, we observed a dual stable platform through the correlation between concentration and the maximum emission wavelength (Fig. 2i). The first platform with the *λ*_em_ of 430 nm occurs at the concentration of as low as 10 nM (Fig. S42†), which corresponds to the intramolecular π-stacking between two aromatic helical components in HM1. The second platform with the *λ*_em_ of 453 nm (Fig. S42†) happens at a concentration of 2.2 μM, which points to the dimeric π-stacking of HM1. We also performed a concentration-dependent fluorescence titration of an *o*-phenanthroline-oxadiazole-based trimer. The spectrum of the *o*-phenanthroline-oxadiazole-based trimer did not show any redshift at a concentration below 100 μM. When the concentration of the trimer is 1 mM, however, the *λ*_em_ peak of the trimers redshifted from 390 nm to 417 nm (Fig. S43†). Hence, the redshift phenomenon on the **HM1** spectrum is its intramolecular and intermolecular fingerprint peaks. To clarify this phenomenon, HM3 with a carboxyl group instead of an ester group was studied. As seen in the fluorescence spectra (Fig. 2f and g), the *λ*_em_ of HM3 also shows remarkable redshift from 417 nm to 447 nm (Fig. S42†) with the concentration increasing from 2 nM to 6 μM. Similarly, a dual stable platform was also observed in HM3 (Fig. 2i), where the *λ*_em_ of HM3 becomes shorter than those of HM1. Importantly, the first platform with the *λ*_em_ of 425 nm begins at 10 nM concentration of HM3 (Fig. 2f), the same as that observed in HM1. This strongly supports the process of intramolecular π-stacking between two aromatic helical components in helical molecular channels. The second platform with the *λ*_em_ of 453 nm appears at the concentration of 3.5 μM (Fig. 2g), which is ascribed to the dimeric π-stacking of HM1. The slight concentration difference in dimeric π-stacking assembly between HM1 and HM3 is mainly due to the hydrogen bonding between carboxyl groups interfering with the π-stacking dimerization. At the same time, the dimeric π-stacking assembly of HM1 and HM3 was investigated by UV-vis spectroscopy. As seen in the UV spectra (Fig. S44†), the assembly concentrations of HM1 and HM3 were found to be 2.5 μM and 3.8 μM, respectively, which are well consistent with the results of fluorescence experiments. Moreover, as the concentration gradually increases, HM1 and HM3 will further self-assemble and generate a fluorescence quenching phenomenon. HM1 displays fluorescence quenching at the concentration of 500 μM (Fig. 2e), while HM3 shows fluorescence quenching at the concentration of 350 μM (Fig. 2h). In addition, atomic force microscopy (AFM) demonstrates that helical molecular channel HM1 is able to assemble into expected linear nanostructures (Fig. S46†). These above observations indicate that helical molecules HM1-HM3 have the potential to form membrane-spanning channels through dimeric π-stacking assembly.

### Ion transport properties of helical molecular channels

**Fig. 3:**
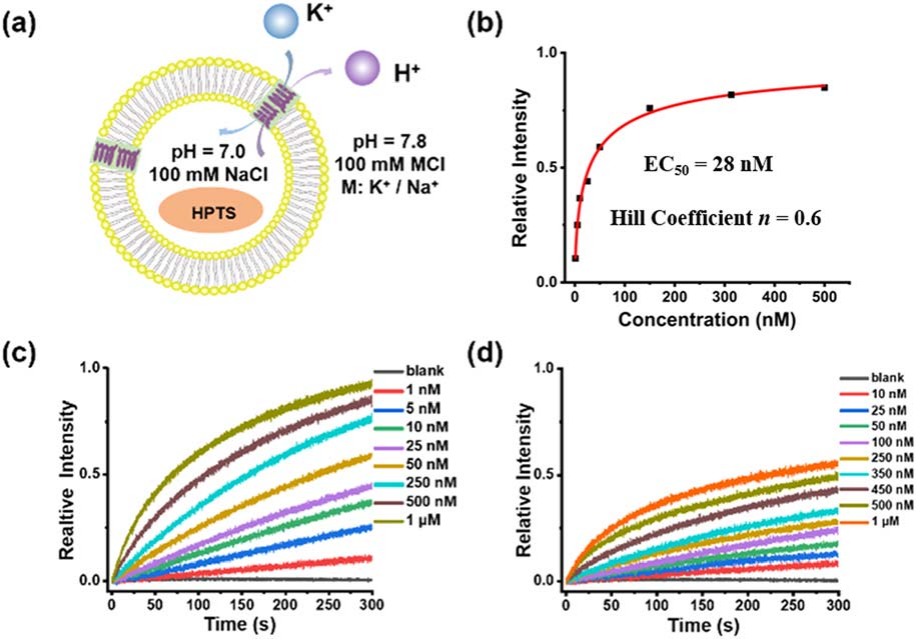
(a) pH-sensitive HPTS assay for ion transport experiment. (b) Hill plot for K^+^ transport by **HM1**. (c) The transport activity of HM1 for K^+^ at different concentrations. (d) The transport activity of HM1 for Na^+^ at different concentrations.

The ion transport properties of helical molecular channels were assessed by vesicle-based kinetic experiments.^24^ As shown in Fig. 3, helical molecular channel HM1 is able to transport potassium ions much faster than sodium ions. According to Hill's analysis of vesicle-based kinetic data (Fig. 3b), the EC_50_ value of HM1 for K^+^ is found as low as 28 nM. However, the EC_50_ value of HM1 for Na^+^ is unable to be calculated due to its low transport activity. To observe the ion binding of HM1, the fluorescence titrations (Fig. S47e†) showed that the binding ability of HM1 to potassium ions is stronger than to sodium ions. In contrast to HM1, the positive charge at the terminal position of HM2 does not completely prevent the transport of potassium ions, but the EC_50_ value of HM2 decreases threefold to 62 nM (Fig. S48c†). Unexpectedly, the presence of positive charges in helical molecular channels remarkably enhances sodium ion bindings, as evidenced by the observation that the binding ability of HM2 to sodium ions is very close to that to potassium ions in the fluorescence titration experiments (Fig. S47f†). The positive charge of HM2 is located at the distal end of the channel, which promotes the dehydration of potassium/sodium ions. Therefore, it is reasonable that HM2 has enhanced binding with Na^+^. Accordingly, HM2 transports sodium ions better than HM1, and its EC_50_ value for Na^+^ was calculated to be 147 nM (Fig. S48d†). This may be due to the influence of positive charges on the hydration structure of sodium ions; it can promote partial dehydration of hydrated sodium. The positive charge at the end of HM2 promotes partial dehydration of hydrated sodium, which makes it easier for free sodium ions to enter the hydrophobic lumen. The positive charge can repel sodium ions, thereby weakening the dipole interaction between sodium ions and water molecules (Na^+^–H_2_O), causing some water molecules to detach from the hydrogenation layer of sodium ions. This is consistent with the influence of the positive charge carried by Lys/Arg near the selective filter of the natural sodium channel on the transmembrane transmission for sodium ions.^25^

In addition, the half-shortened channel HM6 was also studied for comparison. As observed (Fig. S50†), HM6 transports potassium ions faster than sodium ions, illustrating a potassium-preferential ion selectivity. However, compared with helical molecular channel HM1, the transport activity of half-shortened HM6 for potassium ions is significantly reduced, with an EC_50_ value of about 2 μM (Fig. S50a†). The difference in transport activity between helical molecular channel HM1 and half-shortened HM6 is nearly two orders of magnitude, which confirms the importance of synthesizing precise hollow helical molecules to simulate the gA structure. The difference in their activity lies in the formation of ordered channels on the membrane, which is mainly achieved by overcoming entropy. Therefore, **HM1** demonstrates better transmission efficiency than **HM6**. In addition, we have explored the ion transport properties of the HM3 molecule (Fig. S49†). It was found that the transport activity of HM3 for K^+^ (EC_50_ = 142 nM) was reduced almost fivefold compared to **HM1** (EC_50_ = 28 nM), and a significant decrease in the ion selectivity was also observed. This may be due to the fact that hydrolysis of the terminal ester group of HM1 is detrimental for intermolecular π-stacking.

**Fig. 4:**
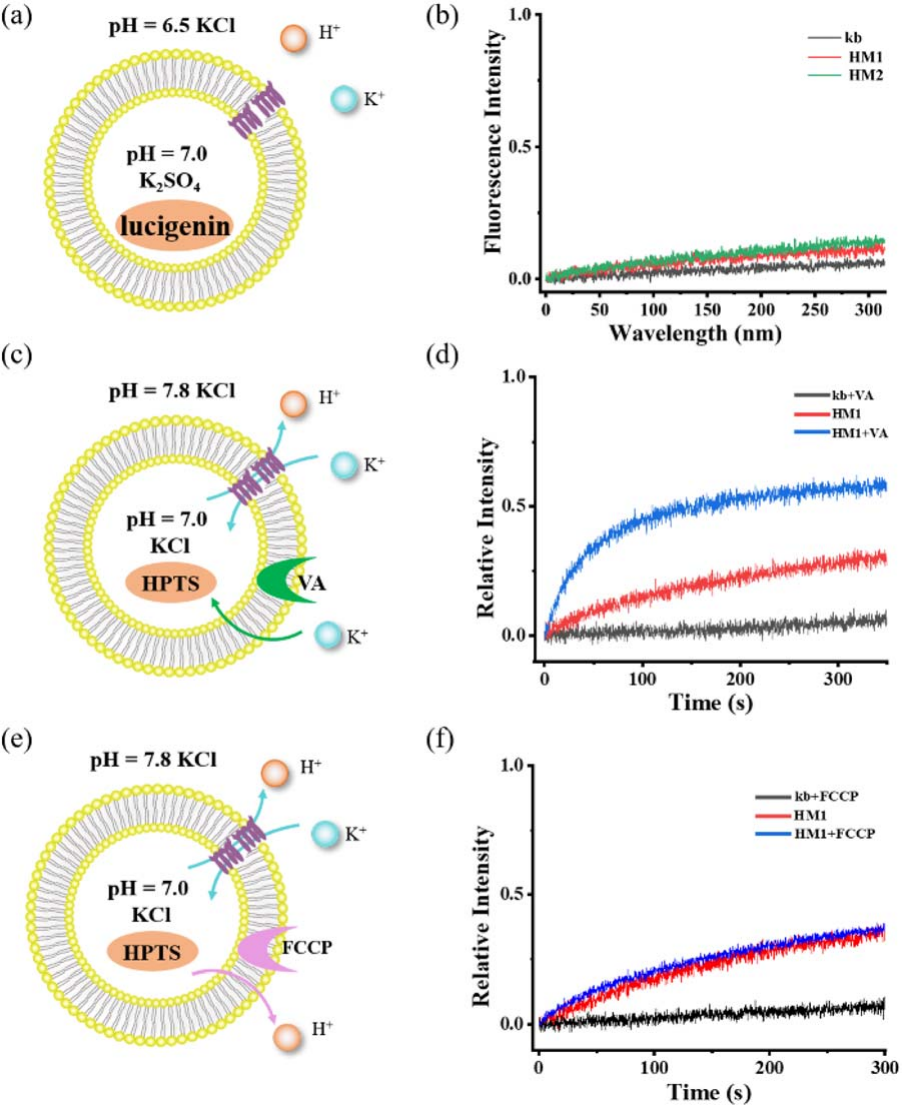
(a) and (b) Detection of chloride ion transport using chloride ion-sensitive lucigenin dye. (c) and (d) Potassium carrier-assisted liposome transport experiment by valinomycin. (e) and (f) Proton carrier-assisted liposome transport experiment by FCCP.

To rule out the ability of gA-like helical molecules to transport chloride across membranes, we designed a control experiment using the chloride-sensitive probe lucigenin dye (Fig. 4a and b). The interior of the vesicle contains pH = 7.0 K_2_SO_4_, while the exterior contains pH = 6.5 KCl buffer. There may be two transmembrane transmission mechanisms in this transport system (H^+^/Cl^−^ symport or K^+^/Cl^−^ antiport). Liposome transport experiments showed that lucigenin dye did not exhibit any obvious quenching phenomena, suggesting that gA-like helical molecules (HM1/HM2) are unable to mediate chloride. To further confirm its K^+^/H^+^ antiport mechanism, we tested the cooperativity in the presence of valinomycin (Fig. 4c and d) and FCCP (Fig. 4e and f). When FCCP was added to the transmission system, the transmission activity of **HM1** remained unaffected. In contrast, there was a significant increase in channel transmission activity after adding valinomycin. Based on these cooperativity experiments, we substantiated the antiport mechanism. Therefore, the gA-like foldamer scaffolds can mediate cation transmembrane transport.

### pH responsiveness of helical molecular channel HM2

**Fig. 5:**
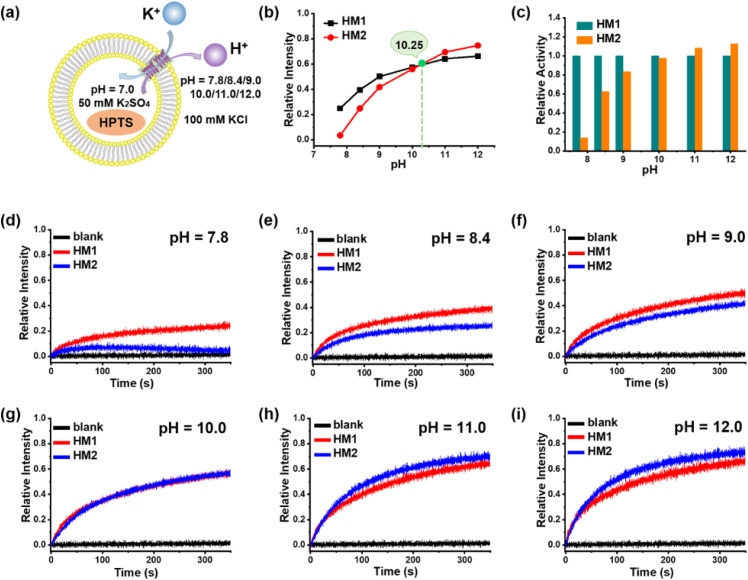
(a) Schematic representation of transmission experiment in liposome vesicle of HM2. (b) and (c) The normalized transport activities of HM1 and HM2 at the concentration of 1 μM with pH variation from 7.8 to 12.0. (d)–(i) Normalized K^+^ transport activities of HM1 and HM2 at the concentration of 1 μM at different pH.

Considering the terminal amine groups of HM2 with protonated and deprotonated states, we investigated the effect of pH on potassium ion transport by means of vesicle-based kinetic experiments. Correspondingly, the external KCl/HEPES buffers with different pH values of 7.8, 8.4, 9.0, 10.0, 11.0, and 12.0 were prepared for the KCl (external) and K_2_SO_4_ (internal, pH = 7.0) vesicle system (Fig. 5a). As the pH increases, the K^+^ transport activity of neutral channel HM1 significantly enhances (Fig. 5b), which is ascribed to the increase in the pH gradient inside and outside the membrane. It is noteworthy that the transport activity of HM2 enhances with the increase in external pH values and ultimately surpasses the transport activity of HM1 (Fig. 5b and c). At the external pH = 7.8, 8.4, and 9.0, respectively, due to the electrostatic repulsion of positive charges, the transport activity of HM2 towards potassium ions was lower than that of HM1 (Fig. 5d–f). To further prove the influence of positive charges on K^+^ transport, we conducted an inhibition experiment using positively charged lysine (Lys) to affect neutral HM1. As seen in Fig. S51,† the transport activity of HM1 for potassium ions significantly decreases after the addition of lysine, confirming that positively charged amino groups compete with potassium ions during transmembrane conduction. At the same time, we added 2-methylheptanoic acid into the transport system of HM1 as a control test, and the addition of 2-methylheptanoic acid was not able to interfere with the transportation of potassium ions of HM1. In addition, when the external pH value is close to the p*K*a (10.0–10.5) of the amine group, the transport ability of HM2 for potassium ions was very similar to that of HM1 (Fig. 5g). Surprisingly, the transport activity of HM2 towards potassium ions exceeded that of HM1 at the external pH = 11.0 and even 12.0 (Fig. 5h and i), suggesting that the bulky Boc groups near the channel entrance constrain ion transport. These observations demonstrate that helical molecular channel HM2 possesses pH-sensitive ion transport function by self-inhibition.

**Fig. 6:**
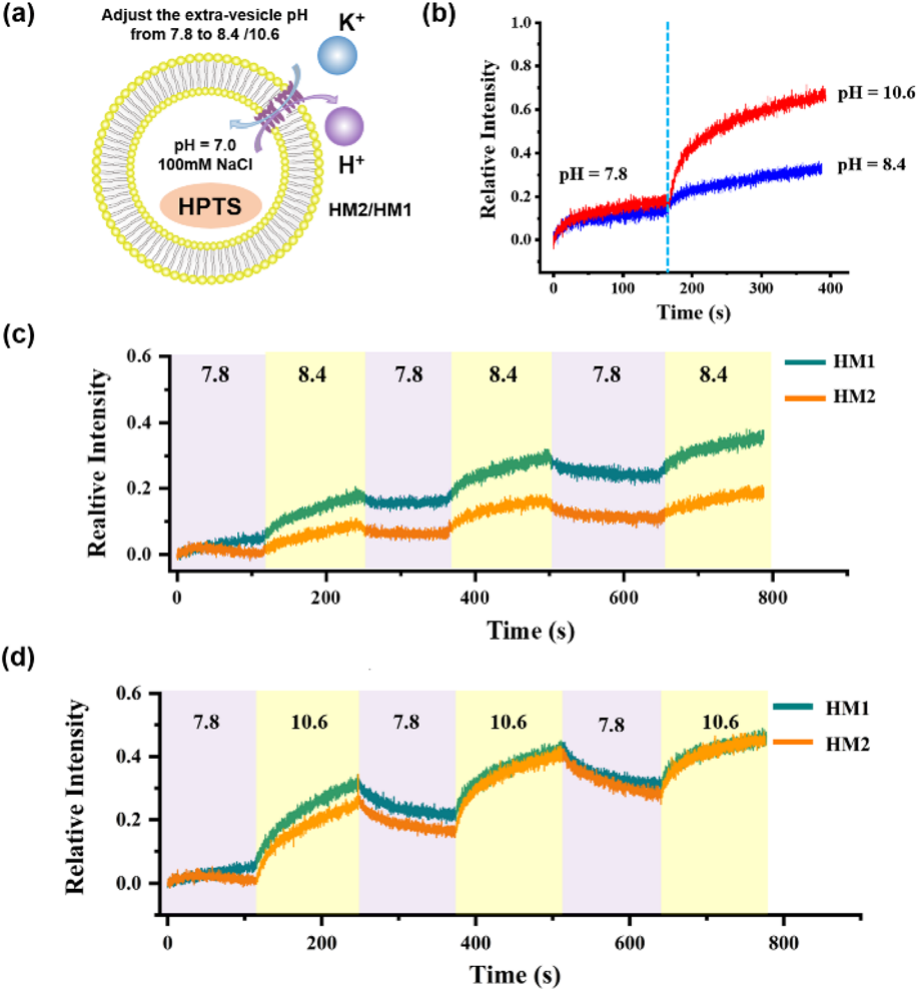
(a) Schematic representation of the transport activity in the liposome vesicle of HM2 and HM1. (b) The regulation of the K^+^ transport of HM2 by adjusting the pH of the external buffer solution (extra-vesicular pH = 8.4, 10.6) in one cycle. (c) The relative intensity of the K^+^ transport of HM2 to three cycling cycles while the extra-vesicular pH varies between 7.8 and 8.4. (d) The relative intensity of the K^+^ transport of HM2 to three cycling cycles while the extra-vesicular pH varies between 7.8 and 10.6.

In order to monitor the pH-responsive transport characteristics of HM2, we established the vesicle-based dynamics experiments with continuously regulated external pH values by adding 1 M KOH and 1 M HCl. In the beginning, the interior of the vesicles was encapsulated with NaCl (pH = 7.0), and the external buffer solution was KCl (pH = 7.8). After 150 s of HM2 addition, the external pH value was adjusted to 8.4 and 10.6, respectively, by adding specific amounts of KOH (1 M) into the vesicle-based system (Fig. S52†). We observed that the relative intensity gradually increases with the increase in pH, indicating that the transport activity of HM2 is gradually enhanced (Fig. 6b). When the external pH value switched between 7.8 and 8.4 or between 7.8 and 10.6, the fluorescence intensities (related to the transport activity of HM2) showed clear pH-responsive changes, and this process was verified for three cycles (Fig. 6c and d). These results demonstrate the pH responsiveness feature of HM2 during ion transmembrane conduction.

### Comparison of ion transport functions between helical molecular channels and gA

**Fig. 7:**
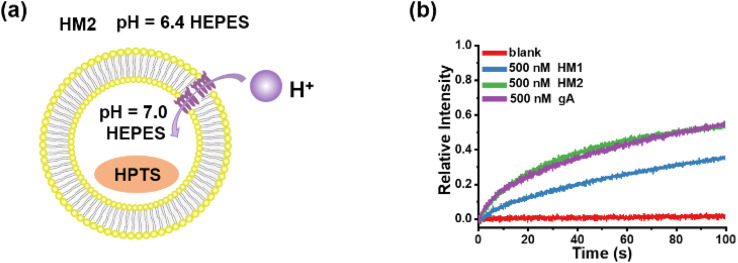
(a) The liposomal diagram for proton transport by HM2. (b) The proton transport activities of HM1, HM2, and gA at a concentration of 500 nM.

Owing to the similarity in structure between helical molecular channels and gA, their ion transport properties were accordingly compared. The proton transport of helical molecular channels was investigated by vesicle-based kinetic experiments, in which the vesicles are composed of egg yolk l-α-phosphatidylcholine (EYPC) (Fig. 7a). The proton gradient inside and outside the vesicles was established by setting the internal pH = 7.0 and the external pH = 6.4, and the proton transport activity can be recorded by the changes in fluorescence intensity within 100 s. As seen in Fig. 7b, gA shows efficient proton transport function at the concentration of 500 nM, and its transport activity can reach 55% in fluorescence intensity. The helical molecular channel HM1 exhibits significant proton transport function, with a 36% increase in fluorescence intensity, but its transport activity is lower than that of gA (Fig. 7b). Very strikingly, molecular channel HM2 exhibits the same proton transport activity as gA under the identical conditions, as evidenced by the increase of 55% in fluorescence intensity (Fig. 7b). Compared to HM1, positively charged HM2 displays higher proton transport activity, indicating that positive charges at the channel entrance can effectively promote proton transport. Therefore, artificial proton channel HM2, constructed through precise structural replication, can compete with gA, presenting a very rare gA mimic. We simultaneously tested the proton transport activity of HM1, HM2, and gA in a pH = 11.0 external buffer solution (Fig. S53†). We found that the proton transport activity of HM2 under deprotonated conditions was comparable to that of HM1, which was much lower than natural gA. We attribute this phenomenon to the positive charge of the protonated amino group at the end of HM2. These results suggested that positive charges near the channel entrance seriously hinder potassium ion transport but significantly promote proton transport, thereby achieving proton transport activity of helical molecular channel HM2 as high as that of natural gA. In addition, protonation and deprotonation of amine groups near the channel entrance allow for pH regulation of transport activity of helical molecular channels, thus the construction of structurally precise and pH-adjusted artificial ion channels for the first time.

### Single-channel conductance behaviour of gA-like molecules

**Fig. 8:**
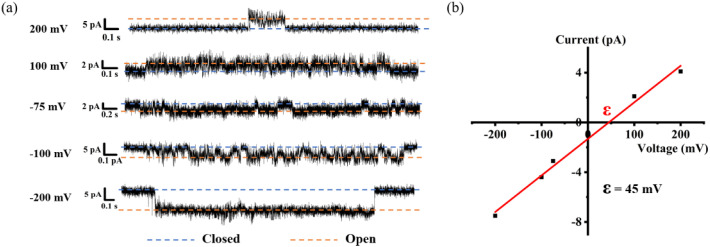
(a) Single-channel electrophysiological recordings from asymmetric BLM experiments of **HM1** (*trans* chamber = 1 M NaCl, *cis* chamber = 1 M KCl). (b) Current–voltage relationship from asymmetric BLM experiments of **HM1** (*ε*_rev_ = 45 mV; *S*_K/Na_ = 4.7).

**Fig. 9:**
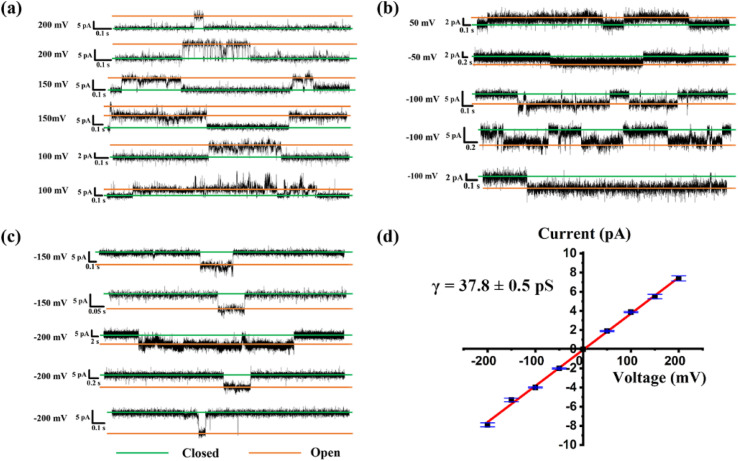
(a)–(c) Single-channel current traces recorded for K^+^ of HM1 at different voltage; the green line shows that the channel is closed and the orange line means that the channel is open; (d) Current–voltage relationship from symmetric BLM experiments for K^+^ of HM1.

**Fig. 10:**
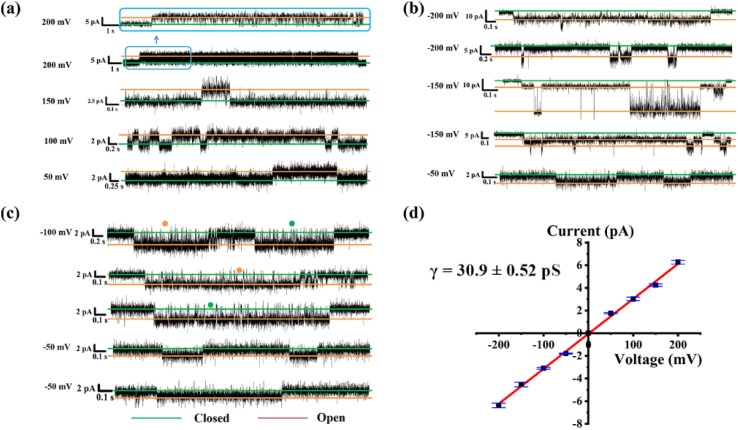
(a)–(c) The single-channel current traces recorded for K^+^ of HM2 at different voltages; the green line shows that the channel is closed and the orange line means that the channel is open. (d) Current–voltage relationship from symmetric BLM experiments for K^+^ of HM2.

**Fig. 11:**
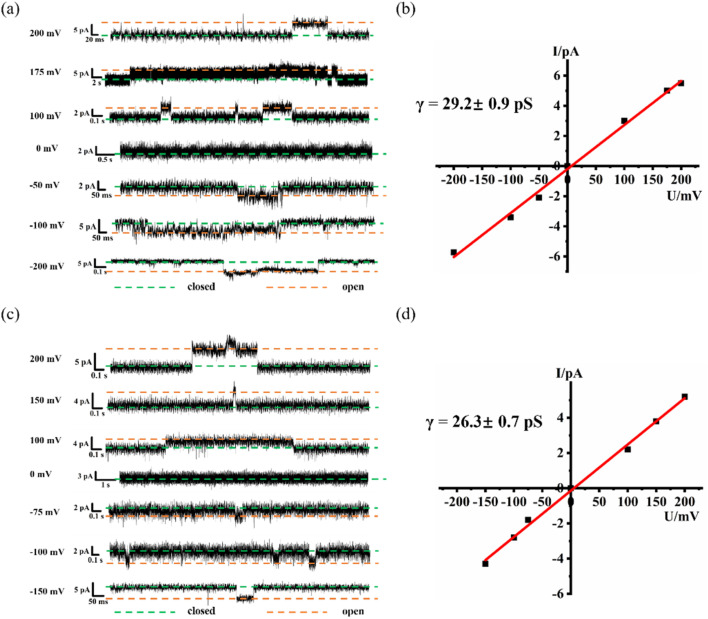
(a) The single-channel current traces recorded for NH_4_^+^ of HM1 at different voltages; the green line shows that the channel is closed, and the orange line means that the channel is open. (b) Current–voltage relationship from symmetric BLM experiments for NH_4_^+^ of HM1. (c) The single-channel current traces recorded for NH_4_^+^ of HM2 at different voltages; the green line shows that the channel is closed, and the orange line means that the channel is open. (d) Current–voltage relationship from symmetric BLM experiments for NH_4_^+^ of HM2.

In addition to proton transport properties, we also tested the potassium ion transport functions of helical molecular channels through single-channel electrophysiological experiments. The channel current traces for HM1 and HM2 using a symmetrical bath (*cis* chamber = *trans* chamber = 1 M KCl, pH = 7.0) were recorded. Notably, the current traces of channels HM1 and HM2 in alkaline conditions (pH = 9.4, 10.6, and 11.0, KCl/HEPES) failed to measure due to the poor stability of the planar phospholipid membrane. At pH = 7.0, regular square signals were observed in the current traces after the addition of helical molecular channels (Fig. 9a and b), demonstrating that helical molecular channels conduct ions through channel mode rather than as ion carriers. Thus, the conductance of HM1 and HM2 for transporting potassium ions was calculated to be 37.8 ± 0.58 pS and 30.9 ± 0.52 pS, respectively (Fig. 9 and 10). Other than this, we were very fortunate to capture some longer-duration open signals. As seen in Fig. S56,†HM1 has an open time of up to 38 s, and longer ones up to 70 s. In Fig. S57,† the open time of HM2 can research 50 s. These data of HM1 and HM2 indicate stable single-channel behavior. The difference in conductance between HM1 and HM2 further indicates that positive charge reduces potassium ion transport rate by about 20%. The potassium transport rate of HM1 reaches 2.3 × 10^7^ ions/s at 100 mV, while the potassium transport rate of gA (the conductance of 26.0 pS)^26^ is calculated to be 1.5 × 10^7^ ions/s under identical conditions, indicating that the conductance of HM1 for transporting potassium ions is almost 1.5 times higher than that of natural gA. This is an important finding because synthetic helical molecular channels significantly exceed natural gA during potassium transmembrane conduction. The natural gA is able to transport NH_4_^+^, so we also investigated the transport conductance of HM1 and HM2 for NH_4_^+^ (*trans* chamber = *cis* chamber = 1 M NH_4_Cl). The transport conductances of HM1 and HM2 molecules for ammonium ions are 29.2 ± 0.9 pS and 26.3 ± 0.7 pS (Fig. 11), respectively. Their transport rates for NH_4_^+^ are almost at the same level as that of natural gA. In addition, we measured *S*_K/Na_ to be 4.7 (Fig. 8b) under asymmetric salt solution (*trans* chamber = 1 M KCl; *cis* chamber = 1 M NaCl).

## Conclusion

In summary, we prepared a type of precise hollow helical molecular channel that structurally resembles natural gA by means of a modular synthesis strategy. Helical molecules are able to form membrane-spanning channels through dimeric π-stacking assembly and show highly efficient ion transport functions. Interestingly, helical molecular channel HM2 with terminal amine groups exhibits pH-regulated ion transport properties. At the same time, we found that the positive charges at the entrance reduce the potassium transport rate of the channel but promote proton transport of the channel. Importantly, the structurally resembling channel HM2 exhibits the same proton transport activity as gA under identical conditions. More importantly, during potassium transmembrane conduction, the synthetically helical molecular channel HM1 significantly surpasses natural gA. At the same time, the transport rate for ammonium ions of HM1 is almost comparable to that of natural gA. This study introduces the first example of structural replication of gA, in which helically folded molecule channels with precisely replicating transmembrane structure yield fantastic ion transport properties, thus presenting structural importance in functional explorations of artificial ion channels.

## Author contributions

J. T. performed synthetic experiments and analyzed the data. Z. D. led the project and designed the methodology. J. T., L. Z., and Z. L. measured the channel properties and titration experiments. J. T., S. M., and Z. D. wrote and revised the manuscript. All authors contributed to discussions and approved the final version.

## Conflicts of interest

The authors declare no conflicts of interest.

## Supplementary Material

SC-016-D5SC01362C-s001

## Data Availability

The data supporting this article have been included as part of the ESI.†
