# Supplementary material for: Helical foldamers replicating membrane-spanning gramicidin a with pH responsiveness and ultrafast potassium permeability
Source: Chem Sci. 2025 Jul 22;16(33):15111–20. doi: 10.1039/d5sc01362c (PMC12282476; doi:10.1039/d5sc01362c)
Supplement: SC-016-D5SC01362C-s001 [file SC-016-D5SC01362C-s001.pdf]

Supplementary Information (SI) for Chemical Science.
This journal is © The Royal Society of Chemistry 2025

Supporting information

Helical foldamer replicating membrane-spanning gramicidin A with pH responsiveness and ultrafast potassium permeability

JunTian,[a, b] Lei Zhang,[a, b] Ze Lin,[a, b] Shizhong Mao,[a] Zeyuan Dong[a, b, *]

[a] State Key Laboratory of Supramolecular Structure and Materials, College of Chemistry, Jilin University, Changchun 130012, China; [b] Center for Supramolecular Chemical Biology, Jilin University, Changchun 130012, China.
* Corresponding author: zdong@jlu.edu.cn

Contents

Supporting information 1
1. General remarks 2
2. Synthesis and characterization of HM1-HM9 2
3. Characteristic spectra of compounds 10
4. The CD spectra of HM1 / HM4 / HM5 / HM7 / HM8 / HM9 63
5. Fluorescence spectra of HM1 / HM3 65
6. The UV spectra of HM1 / HM3 / HM4 66
7. The assembly of HM1 / HM2 on the silicon wafer surface 67
8. Fluorescence titration experiments of ion-bounding 68
9. Ion transport experiments using HPTS assay 69
10. The inhibition for K^+ transport by Lys of HM1 71
11. Continuous regulation of the pH-responsive behaviour for potassium of HM2 72
12. Proton transfer experiment 72
13. The selectivity ($S_{K/Na}$) of HM1 / HM2 / HM6 73
14. Planar lipid bilayer conductance experiment 74
15. Supplementary image 76

1. General remarks

All reagents are provided by the commercial supplier. Aqueous solution was prepared from MilliQ water, N, N-dimethylformamide (DMF), triethylamine (TEA), chloroform ($CHCl_3$) were dried with CaH_2 for ten days then it was distilled and stored hermetically in saddle bottles for use. Aqueous solution was prepared from MilliQ water. All the reactions were monitored through thin layer chromatography (TLC) and observed with Portable UV lamps (UV), while column chromatography purifications were carried out via silica gel. ^{1}H NMR and ^{13}C NMR spectra were recorded on the WNMR-I 400 and Bruker AVANCEⅢ 500. The solvents signals of $CDCl_3$ ^{1}H NMR spectrum were referenced at δ = 7.26, respectively. ^{1}H NMR data are recorded in the order: chemical shift (ppm), multiplicity (s = singlet, d = doublet, t = triplet, m = multiplet, br = broad), the number of protons. The solvents signals of $CDCl_3$ for ^{13}C NMR spectrum were referenced at δ = 77.16 and 39.52 ppm, respectively. The solvent signals of CD_3CN (Acetonitrile-d3) for ^{1}H NMR and ^{13}C NMR were referenced at δ = 1.94 ppm and 1.32, 118.26 ppm, respectively. The mass spectra were obtained on an HP1100EMD (electrospray ionization mass spectrometry, ESI MS).

2. Synthesis and characterization of HM1-HM9

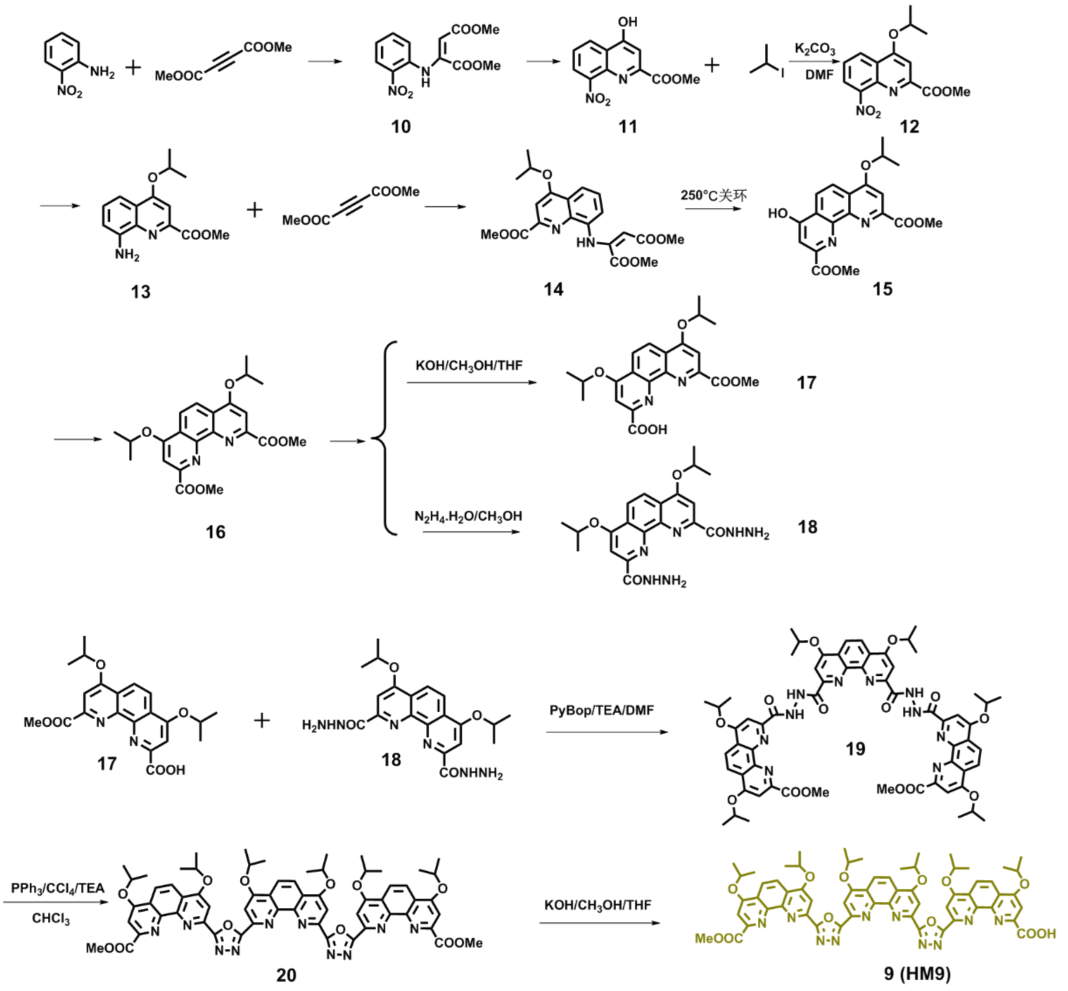

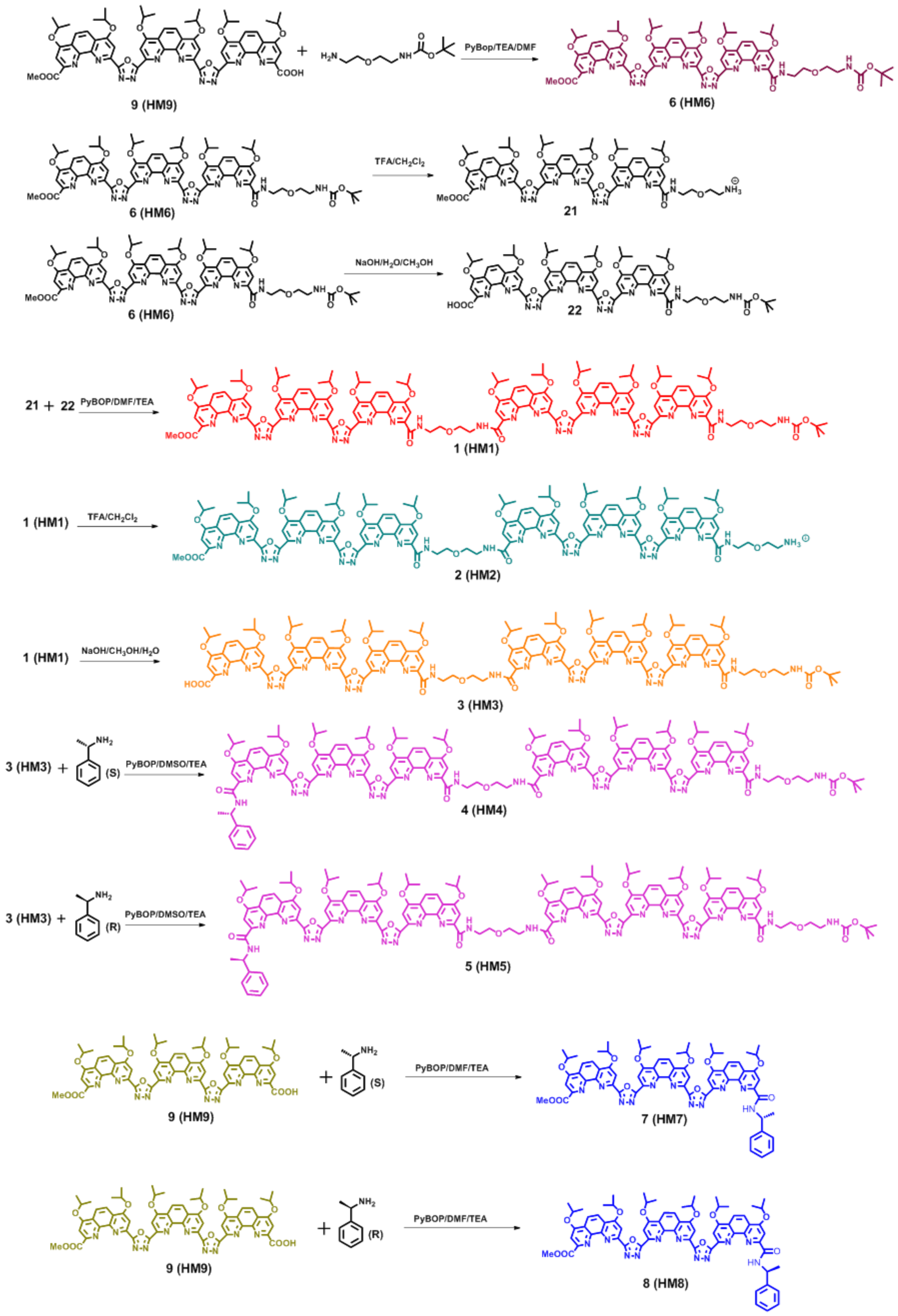

Scheme S1. Synthesis route of compounds 1-22.

Compounds 10-18 were synthesized according to previously reported procedures.[S1]

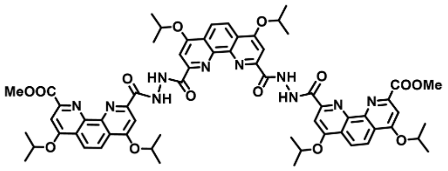

19-Chemical Formula: $C_{62}H_{64}N_{10}O_{14}$

Compound 19. Compound 17 (4.05 g, 10.17 mmol) and compound 18 (2.0 g, 4.85 mmol) was dissolved in dry DMF (50 mL), then PyBop (6.05 g, 11.63 mmol), TEA (7 mL, 50.87 mmol) was added in the solution. The resulting mixture was reacted under inert N_2 atmosphere and stirred at 45℃ for 24 hours, after which volatiles were removed in vacuum. The crude product was purified by silica gel column chromatography using dichloromethane and methanol (40:1, v/v) for two times to yield 11 as light yellow powder (3.8g, 63%). ^{1}HNMR(400 MHz, $CDCl_3$/Trifluoroacetic acid-D 50:1) δ 8.52 (d, J = 12.0 Hz, 4H), 8.32 (d, J = 9.3 Hz, 2H), 8.19 (s, 2H), 8.01 (d, J = 17.5 Hz, 4H), 5.36 (d, J = 10.8, 4.7 Hz, 5H), 5.11 (s, 2H), 4.18 (d, J = 12.5 Hz, 7H), 1.70 (d, J = 12.1, 6.0 Hz, 31H), 1.57 (d, J = 6.0 Hz, 14H). ^{13}C NMR (126 MHz, $CDCl_3$) δ 169.77, 162.88, 150.49, 150.17, 141.76, 137.98, 137.57, 137.07, 124.79, 124.01, 123.78, 122.98, 122.34, 122.14, 121.71, 73.81, 54.83, 54.52, 46.84, 21.19. ESI m/z: calculated for $[M+H]^+$ $C_{62}H_{65}N_{10}O_{14}$ 1273.5; Found 1273.5.

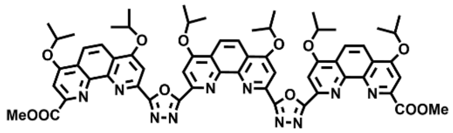

20-Chemical Formula: $C_{62}H_{60}N_{10}O_{12}$

Compound 20. A solution of 19 (2 g, 1.77 mmol) in dry $CHCl_3$ (15 mL) was added a solution of PPh_3 (1.34 g, 5.12 mmol), CCl_4 (1.75 mL, 17.1 mmol) and TEA (2.46 mL, 17.1 mmol), and the resulting solution was stirred at 75℃ for two days. The volatiles were removed under reduced pressure to give a solid then dried in dried in vacuum oven after which the crude product was purified by silica gel column chromatography with eluent dichloromethane and methanol (50:1, v/v) to obtain the powder of compound 20 (1.24 g, 75.6%). ^{1}H NMR (400 MHz, $CDCl_3$/Trifluoroacetic acid-D 50:1) δ 8.48 (s, 2H), 8.29 (s, 2H), 8.01 (s, 2H), 7.93 (d, J = 9.3 Hz, 2H), 7.85 (d, J = 9.3 Hz, 2H), 7.07 (s, 2H), 5.23 (d, J = 3.8 Hz, 4H), 4.91 – 4.79 (m, 2H), 3.77 (s, 6H), 1.86 – 1.56 (m, 42H), 1.45 (d, J = 5.8 Hz, 7H). ^{13}C NMR (126 MHz, $CDCl_3$) δ 169.77, 162.88, 150.49, 150.17, 141.76, 137.98, 137.57, 137.07, 124.79, 124.01, 123.78, 122.98, 122.34, 122.14, 121.71, 73.81, 54.83, 54.52, 46.84, 21.19. ESI m/z: calculated for $[M+H]^+$ $C_{62}H_{61}N_{10}O_{12}$ 1237.4; Found1137.4; calculated for $[M+K]^+$ $C_{62}H_{60}N_{10}O_{12}K$ 1175.4; Found 1175.4

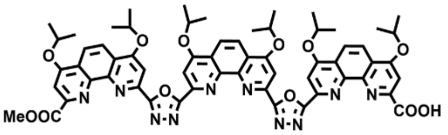

9-Chemical Formula: $C_{61}H_{58}N_{10}O_{12}$

Compound 9. A solution of KOH (12.7 mg, 0.23 mmol) in methanol was added to the solution of compound 20 (235 mg, 0.21 mmol) dissolved in acetonitrile(5 mL) at 0℃,what is more, 30 minutes later, 200 μL water was added to the reaction mixture then the resulting solution was stirred at room temperature for 24 hours. The reaction mixture was quenched with 0.1 M HCl. The organic solvents were removed by rotary evaporation to get the crude product meanwhile the crude product was extracted with dichloromethane (30 mL) and water (20 mL). The organic solvents were removed by rotary evaporation to obtain the final product (185 mg, 80%). ^{1}HNMR (400 MHz, $CDCl_3$/ Trifluoroacetic acid-D 50:1) δ 8.65 – 8.54 (m, 4H), 8.45 – 8.31 (m, 5H), 8.17 (s, 1H), 8.06 (d, J = 11.7 Hz, 2H), 5.66 – 5.15 (m, 7H), 4.25 (s, 3H), 1.85 – 1.62 (m, 42H). ^{13}C NMR (126 MHz, $CDCl_3$) δ 169.77, 162.88, 150.49, 150.17, 141.76, 137.98, 137.57, 137.07, 124.79, 124.01, 123.78, 122.98, 122.34, 122.14, 121.71, 73.81, 54.83, 54.52, 46.84, 21.19. ESI m/z: calculated for $[M+H]^+$ $C_{61}H_{59}N_{10}O_{12}$ 1123.4; Found 1123.4; calculated for $[M+K]^+$ $C_{62}H_{58}N_{10}O_{12}K$ 1161.4; Found 1161.4.

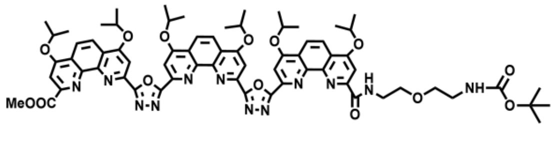

6-Chemical Formula: $C_{70}H_{76}N_{12}O_{14}$

Compound 6. Dry TEA (0.325 mL, 4.5 mmol) was added to a solution of compound 9 (506 mg, 0.45 mmol), tert-butyl [2-(2-aminoethoxy) ethyl] carbamate (184 mg, 0.9 mmol) and PyBop (586 mg, 1.12 mmol) in dry DMF (2 mL) under inert N_2 atmosphere. The DMF was evaporated under vacuum while the mixture was stirred 12 hours at 35℃. Then the residue was purified through silica gel column chromatography with the eluent of dichloromethane and methanol to obtain light yellow solid (500 mg, 84%). ^{1}H NMR (400 MHz, $CDCl_3$/ Trifluoroacetic acid-D 50:1) δ 8.62 – 8.09 (m, 12H), 5.64 – 5.13 (m, 6H), 4.20 (s, 3H), 3.84 – 3.52 (m, 7H), 3.32 (t, J = 5.2 Hz, 3H), 1.80 – 1.55 (m, 44H). ^{13}C NMR (126 MHz, $CDCl_3$) δ 169.77, 162.88, 150.49, 150.17, 141.76, 137.98, 137.57, 137.07, 124.79, 124.01, 123.78, 122.98, 122.34, 122.14, 121.71, 73.81, 54.83, 54.52, 46.84, 21.19. ESI m/z: calculated for $[M+H]^+$ $C_{70}H_{77}N_{12}O_{14}$ 1310.4; Found 1310.1; calculated for $[M+K]^+$ $C_{70}H_{76}N_{12}O_{14}K$ 1348.4; Found 1348.1; calculated for $[M+Na]^+$ $C_{70}H_{76}N_{12}O_{14}Na$ 1332.4; Found 1332.1

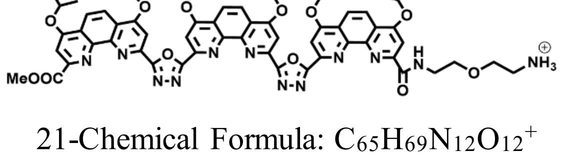

21-Chemical Formula: $C_{65}H_{69}N_{12}O_{12}^+$

Compound 21. Trifluoroacetic acid (1.5 mL) was added dropwise to a solution of compound 6 (423 mg, 0.32 mmol) in dichloromethane (4 mL) under nitrogen at 0°C and stirred at room temperature for 2 days. The organic solvents was removed under reduced pressure after that the crude product was dispersed in Ethyl ether. After

filtration, the precipitate was washed with ether. ^{1}HNMR (500 MHz, $CDCl_3$/ Trifluoroacetic acid-D 50:1) δ 8.60 – 7.99 (m, 12H), 5.61 – 5.34 (m, 5H), 5.19 (d, J = 12.4, 6.1 Hz, 1H), 4.21 (d, J = 8.9 Hz, 3H), 3.79 (d, J = 8.0 Hz, 6H), 3.42 (s, 2H), 1.91 – 1.49 (m, 30H). ^{13}C NMR (126 MHz, $CDCl_3$) δ 169.77, 162.88, 150.49, 150.17, 141.76, 137.98, 137.57, 137.07, 124.79, 124.01, 123.78, 122.98, 122.34, 122.14, 121.71, 73.81, 54.83, 54.52, 46.84, 21.19. ESI m/z: calculated for $[M+H]^+$ $C_{65}H_{69}N_{12}O_{12}$ 1209.5; Found 1209.5.

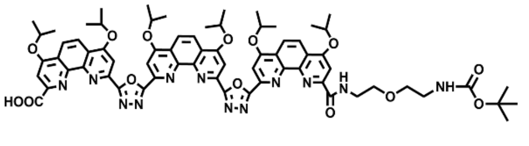

22-Chemical Formula: $C_{69}H_{74}N_{12}O_{14}$

Compound 22. A solution of NaOH (37 mg, 0.9 mmol) in water was added to the solution of compound 6 (489 mg, 0.37 mmol) dissolved in methanol (5 mL) at room temperature. After 2 days, the volatiles were removed under reduced pressure which can got the crude product. The crude product was dispersed in water then the suspension was neutralized with 0.1 M HCl and filtered with purified water. The filter residue was totally dried on vacuum at 50℃ for 12 hours to get 0.9 g a yellowish solid. ^{1}HNMR (500 MHz, $CDCl_3$/ Trifluoroacetic acid-D 50:1) δ 8.58 – 7.97 (m, 12H), 5.50 – 5.16 (m, 7H), 3.84 – 3.55 (m, 7H), 3.37 – 3.30 (m, 2H), 3.20 (d, J = 8.6, 4.7 Hz, 1H), 1.96 – 1.45 (m, 36H). ^{13}C NMR (126 MHz, $CDCl_3$) δ 169.77, 162.88, 150.49, 150.17, 141.76, 137.98, 137.57, 137.07, 124.79, 124.01, 123.78, 122.98, 122.34, 122.14, 121.71, 73.81, 54.83, 54.52, 46.84, 21.19. ESI m/z: calculated for $[M+H]^+$ $C_{69}H_{75}N_{12}O_{14}$ 1295.5; Found 1295.6.

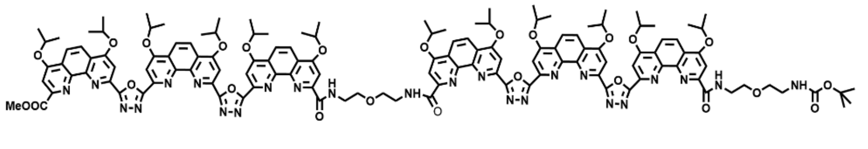

1-Chemical Formula: $C_{134}H_{140}N_{24}O_{25}$

Compound 1. Compound 21 (340 mg, 0.28 mmol), compound 22 (371 mg, 0.29 mmol), were dissolved in dry DMF (5 mL), then dry TEA (0.203 ml, 1.4 mmol, 5 eq) was added to the reaction mixture. After the compound 21 and compound 22 was totally dissolution in the organic solvent, PyBop (439 mg, 0.84 mmol, 3 eq) was added under inert atmosphere of N_2 at room temperature. The reaction mixture was stirred at room temperature for 1 hours after which the mixture was stirred at 45℃ for 3 days. The volatiles were removed in vacuum and the residue was sufficient dried in vacuum at 50℃， then the crude product was purified through silica gel column chromatography by using the eluent of dichloromethane and methanol (40:1,v/v). Ultimately, the pure compound 1 (350 mg, 51.2%) was got. ^{1}HNMR (500 MHz, $CDCl_3$/ Trifluoroacetic acid-D 50:1) δ 8.66 – 8.03 (m, 24H), 5.33 (d, J = 25.4, 18.6 Hz, 12H), 4.21 (s, 3H), 3.74 (d, J = 60.1, 22.0 Hz, 13H), 3.34 (s, 3H), 2.08 – 1.13 (m, 87H). ^{13}C NMR (126 MHz, $CDCl_3$) δ 169.77, 162.88, 150.49, 150.17, 141.76, 137.98, 137.57, 137.07, 124.79,

124.01, 123.78, 122.98, 122.34, 122.14, 121.71, 73.81, 54.83, 54.52, 46.84, 21.19. ESI m/z: calculated for [M+H] $^{+}$ $C_{134}H_{140}N_{24}O_{25}$ 2486.0; Found 2486.0.

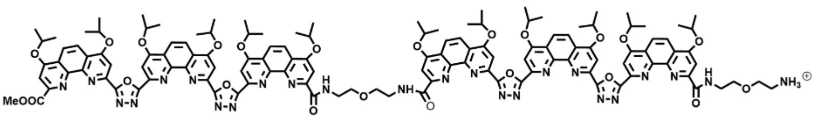

2-Chemical Formula: $C_{129}H_{133}N_{24}O_{23}$

Compound 2. The compound 1 (380 mg, 0.15 mmol) was dissolved in DCM (4 mL), TFA (2 mL) was added into the solution in two parts at ice bath and stirred at room temperature for 2 days. The organic solvents were removed by rotary evaporation after that the crude product was totally dispersed in Ethyl ether and adequately ultrasonic 10 minutes. The suspension was filtered to get compound 2. ^{1}HNMR (500 MHz, $CDCl_3$/ Trifluoroacetic acid-D 50:1) δ 8.69 – 7.98 (m, 24H), 5.50 – 5.10 (m, 12H), 4.19 (d, J = 14.6, 7.5 Hz, 3H), 3.70 (d, J = 55.9 Hz, 15H), 3.35 (s, 3H), 2.06 – 1.38 (m, 70H). ^{13}C NMR (126 MHz, $CDCl_3$) δ 169.77, 162.88, 150.49, 150.17, 141.76, 137.98, 137.57, 137.07, 124.79, 124.01, 123.78, 122.98, 122.34, 122.14, 121.71, 73.81, 54.83, 54.52, 46.84, 21.19. ESI m/z: calculated for [M+H] $^{+}$$C_{129}H_{134}N_{24}O_{23}$ 2386.9; Found 2386.9.

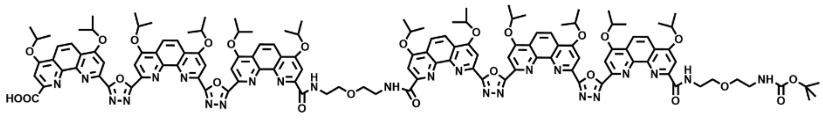

3-Chemical Formula: $C_{133}H_{138}N_{24}O_{25}$

Compound 3. The compound 1 (150 mg, 0.061mmol) was dissolved in CH_3OH (4 mL), NaOH (20mg, 0.5 mmol) and H_2O (1 mL) was added into the solution stirred at room temperature for 2 days. The organic solvents were removed by rotary evaporation after that the crude product was totally dispersed in water (20 mL) then the suspension was neutralized with 0.1 M HCl and filtered with purified water. The filter residue was totally dried on vacuum at 50℃ for 12 hours to get 140 mg product. ^{1}H NMR (500 MHz, $CDCl_3$) δ 8.63 – 8.01 (m, 24H), 5.39 (s, 8H), 5.20 (d, *J* = 54.5 Hz, 5H), 3.82 (d, *J* = 16.8 Hz, 11H), 3.67 (d, *J* = 5.4 Hz, 3H), 3.37 (dd, *J* = 15.8, 10.5 Hz, 3H), 3.22 (dd, *J* = 8.6, 4.6 Hz, 3H), 1.84 – 1.20 (m, 75H). ^{13}C NMR (126 MHz, $CDCl_3$) δ 169.77, 162.88, 150.49, 150.17, 141.76, 137.98, 137.57, 137.07, 124.79, 124.01, 123.78, 122.98, 122.34, 122.14, 121.71, 73.81, 54.83, 54.52, 46.84, 21.19. ESI m/z: calculated for [M+H] $^{+}$ $C_{133}H_{139}N_{24}O_{25}$ 2472.0; Found 2472.0.

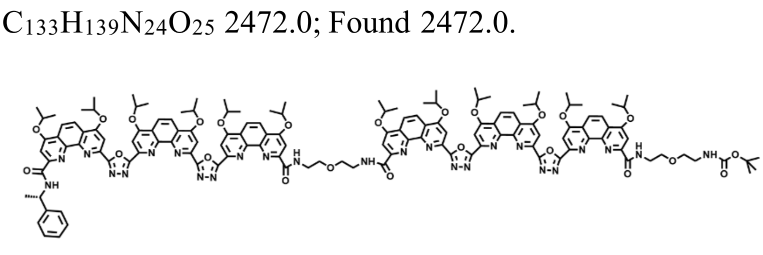

4-Chemical Formula: $C_{141}H_{147}N_{25}O_{24}$

Compound 4. Compound 3 (20 mg, 0.0081 mmol), (*S*)-1-phenylethanamine (100 μL, 0.77 mmol), were dissolved in dry DMSO (0.5 mL), then dry TEA (0.5 ml, 3.58 mmol) was added to the reaction mixture. After the compound 3 and N-methylbenzylamine

was totally dissolution in the organic solvent, PyBop (50 mg, 0.096 mmol) was added under inert atmosphere of N_2 at room temperature. The reaction mixture was stirred at room temperature for 12 hours. The volatiles were removed in vacuum and the crude product was dispersed into ultrapure water and was fully sonicated then filtration to obtain pure product. Ultimately, the pure compound 4 (15 mg, 72.1%) was got. ^{1}H NMR (400 MHz, $CDCl_3$) δ 8.66 – 8.10 (m, 24H), 7.48 – 7.36 (m, 9H), 5.38 (s, 12H), 3.67 (s, 15H), 3.43 (s, 8H), 2.13 (s, 183H). ^{13}C NMR (126 MHz, $CDCl_3$) δ 169.77, 162.88, 150.49, 150.17, 141.76, 137.98, 137.57, 137.07, 124.79, 124.01, 123.78, 122.98, 122.34, 122.14, 121.71, 73.81, 54.83, 54.52, 46.84, 21.19. ESI m/z: calculated for $[M+H]^+$ $C_{141}H_{147}N_{25}O_{24}$ 2575.0; Found 2575.0.

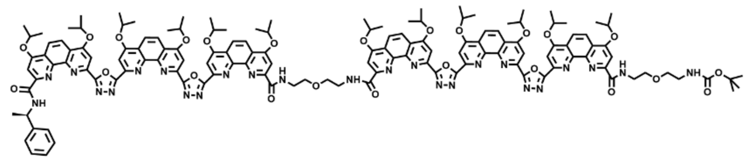

5-Chemical Formula: $C_{141}H_{147}N_{25}O_{24}$

Compound 5. Compound 3 (15 mg, 0.0061 mmol), (*R*)-1-phenylethanamine (100 μL, 0.77 mmol), were dissolved in dry DMSO (0.5 mL), then dry TEA (0.5 ml, 3.58 mmol) was added to the reaction mixture. After the compound 3 and N-Methylbenzylamine was totally dissolution in the organic solvent, PyBop (50 mg, 0.096 mmol) was added under inert atmosphere of N_2 at room temperature. The reaction mixture was stirred at room temperature for 12 hours. The volatiles were removed in vacuum and the crude product was dispersed into ultrapure water and was fully sonicated then filtration to obtain pure product. Ultimately, the pure compound 5 (10.6 mg, 70.1%) was got. ^{1}H NMR (400 MHz, $CDCl_3$) δ 8.66 – 8.10 (m, 24H), 7.48 – 7.36 (m, 9H), 5.38 (s, 12H), 3.67 (s, 15H), 3.43 (s, 8H), 2.13 (s, 183H). ^{13}C NMR (126 MHz, $CDCl_3$) δ 169.77, 162.88, 150.49, 150.17, 141.76, 137.98, 137.57, 137.07, 124.79, 124.01, 123.78, 122.98, 122.34, 122.14, 121.71, 73.81, 54.83, 54.52, 46.84, 21.19. ESI m/z: calculated for $[M+H]^+$ $C_{141}H_{147}N_{25}O_{24}$ 2575.0; Found 2575.0.

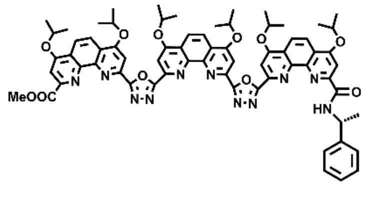

7- Chemical Formula: $C_{69}H_{67}N_{11}O_{11}$

Compound 7. Compound 9 (20 mg, 0.018mmol), (*S*)-1-phenylethanamine (100 μL, 0.77 mmol), were dissolved in dry DMSO (0.5 mL), then dry TEA (0.5 ml, 3.58 mmol) was added to the reaction mixture. After the compound 9 and N-Methylbenzylamine was totally dissolution in the organic solvent, PyBop (18.5 mg, 0.036 mmol) was added under inert atmosphere of N_2 at room temperature. The reaction mixture was stirred at room temperature for 12 hours. The volatiles were removed in vacuum and the crude product was dispersed into ultrapure water and was fully sonicated then filtration to

obtain pure product. Finally, the pure compound 7 (17 mg, 76.6%) was got. ^{1}H NMR (500 MHz, $CDCl_3$) δ 8.56 (dd, *J* = 12.9, 9.3 Hz, 1H), 8.47 – 8.22 (m, 2H), 8.06 (s, 1H), 7.49 – 7.33 (m, 1H), 5.67 – 5.10 (m, 2H), 4.21 (d, *J* = 29.2 Hz, 1H), 2.13 – 1.44 (m, 11H). ^{13}C NMR (126 MHz, $CDCl_3$) δ 169.77, 162.88, 150.49, 150.17, 141.76, 137.98, 137.57, 137.07, 124.79, 124.01, 123.78, 122.98, 122.34, 122.14, 121.71, 73.81, 54.83, 54.52, 46.84, 21.19. ESI m/z: calculated for $[M+H]^{+}$ $C_{141}H_{147}N_{25}O_{24}$ 1226.5; Found 1226.5.

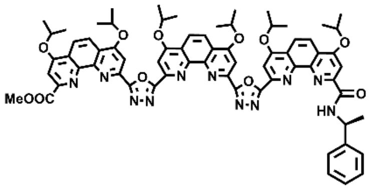

8-Chemical Formula: $C_{69}H_{67}N_{11}O_{11}$

Compound 8. Compound 9 (20 mg, 0.018 mmol), (*R*)-1-phenylethanamine (100 µL, 0.77 mmol), were dissolved in dry DMSO (0.5 mL), then dry TEA (0.5 ml, 3.58 mmol) was added to the reaction mixture. After the compound 9 and N-Methylbenzylamine was totally dissolution in the organic solvent, PyBop (18.5 mg, 0.036 mmol) was added under inert atmosphere of N_2 at room temperature. The reaction mixture was stirred at room temperature for 12 hours. The volatiles were removed in vacuum and the crude product was dispersed into ultrapure water and was fully sonicated then filtration to obtain pure product. In the end the pure compound 8 (15.8 mg, 71.1%) was got. ^{1}H NMR (500 MHz, $CDCl_3$) δ 8.56 (dd, *J* = 12.9, 9.3 Hz, 1H), 8.47 – 8.22 (m, 2H), 8.06 (s, 1H), 7.49 – 7.33 (m, 1H), 5.67 – 5.10 (m, 2H), 4.21 (d, *J* = 29.2 Hz, 1H), 2.13 – 1.44 (m, 11H). ^{13}C NMR (126 MHz, $CDCl_3$) δ 169.77, 162.88, 150.49, 150.17, 141.76, 137.98, 137.57, 137.07, 124.79, 124.01, 123.78, 122.98, 122.34, 122.14, 121.71, 73.81, 54.83, 54.52, 46.84, 21.19. ESI m/z: calculated for $[M+H]^{+}$ $C_{141}H_{147}N_{25}O_{24}$ 1226.5; Found 1226.5.

3. Characteristic spectra of compounds

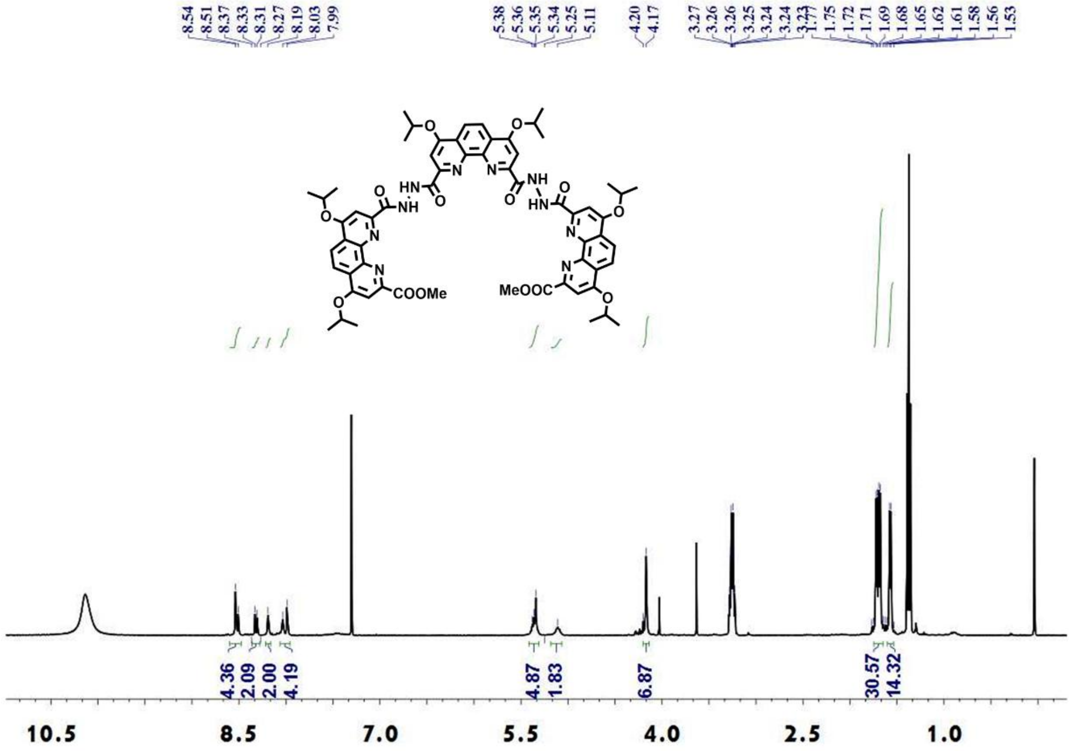

Figure S1. ^{1}H NMR spectrum of compound 19 in $CDCl_3$ ($CDCl_3$/ Trifluoroacetic acid-D 50:1).

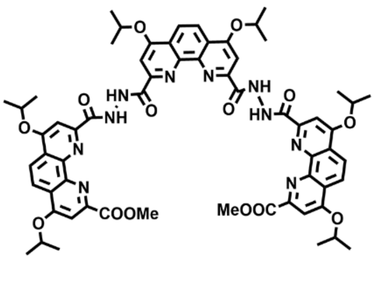

M_{14}=1172.4

ESI-full spectrum

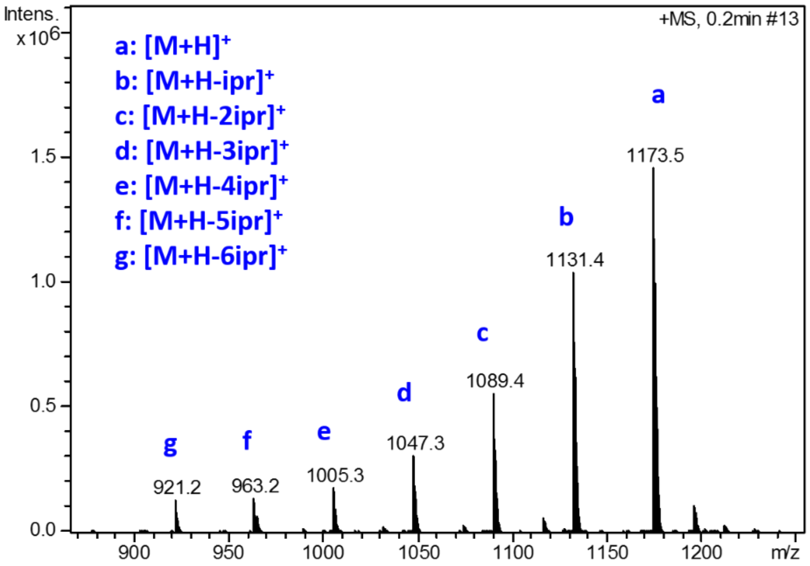

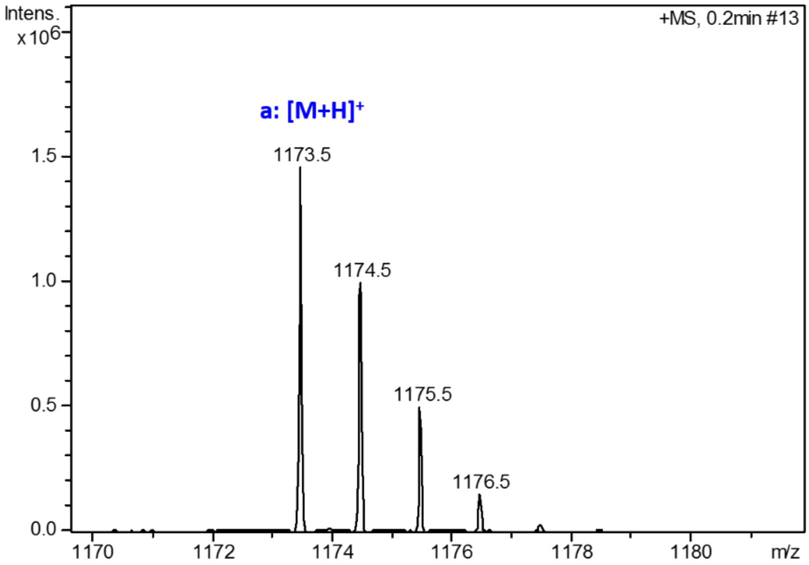

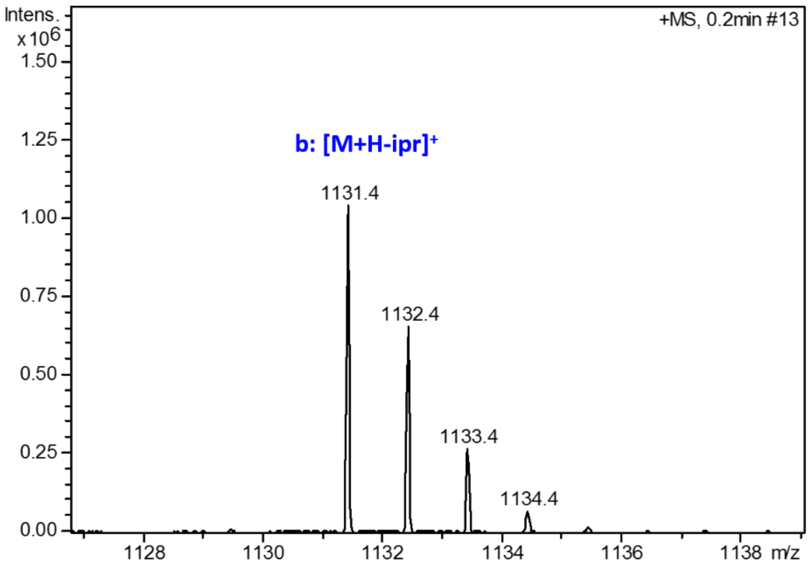

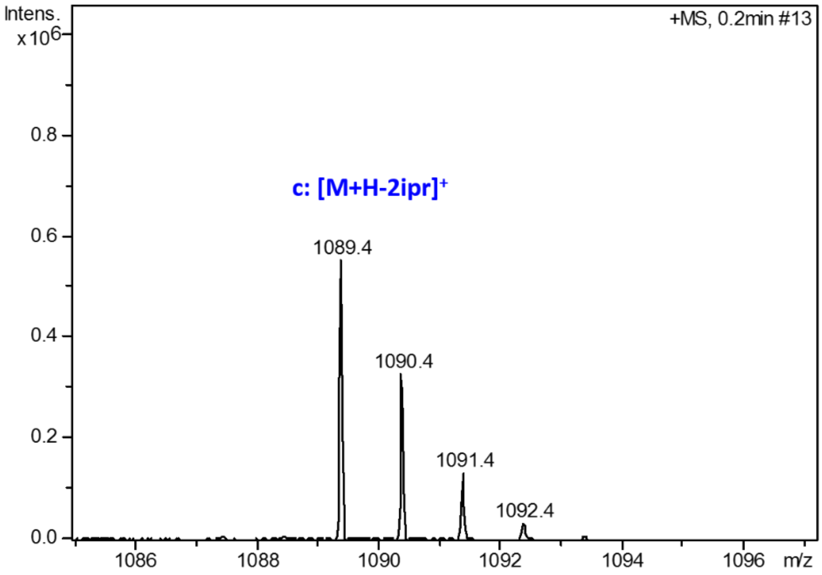

Intens.
x10^6
+MS, 0.2min #13
c: [M+H-2ipr]+
1089.4
1090.4
1091.4
1092.4
m/z

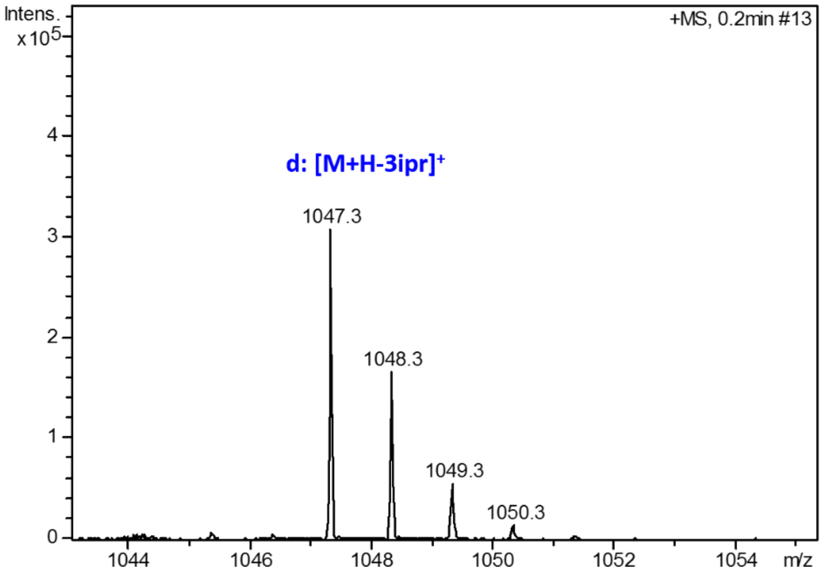

Intens.
x10^5
+MS, 0.2min #13
d: [M+H-3ipr]+
1047.3
1048.3
1049.3
1050.3
m/z

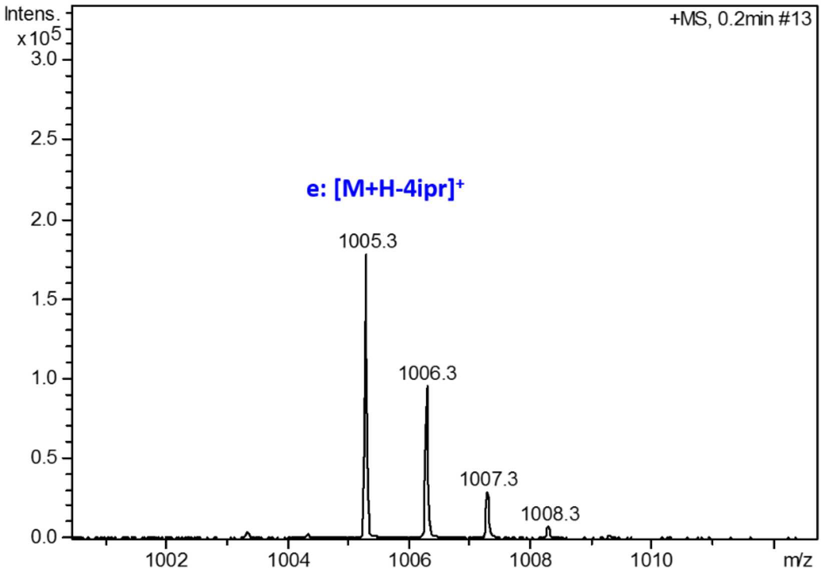

Intens.
x10^5
+MS, 0.2min #13
e: [M+H-4ipr]+
1005.3
1006.3
1007.3
1008.3
m/z

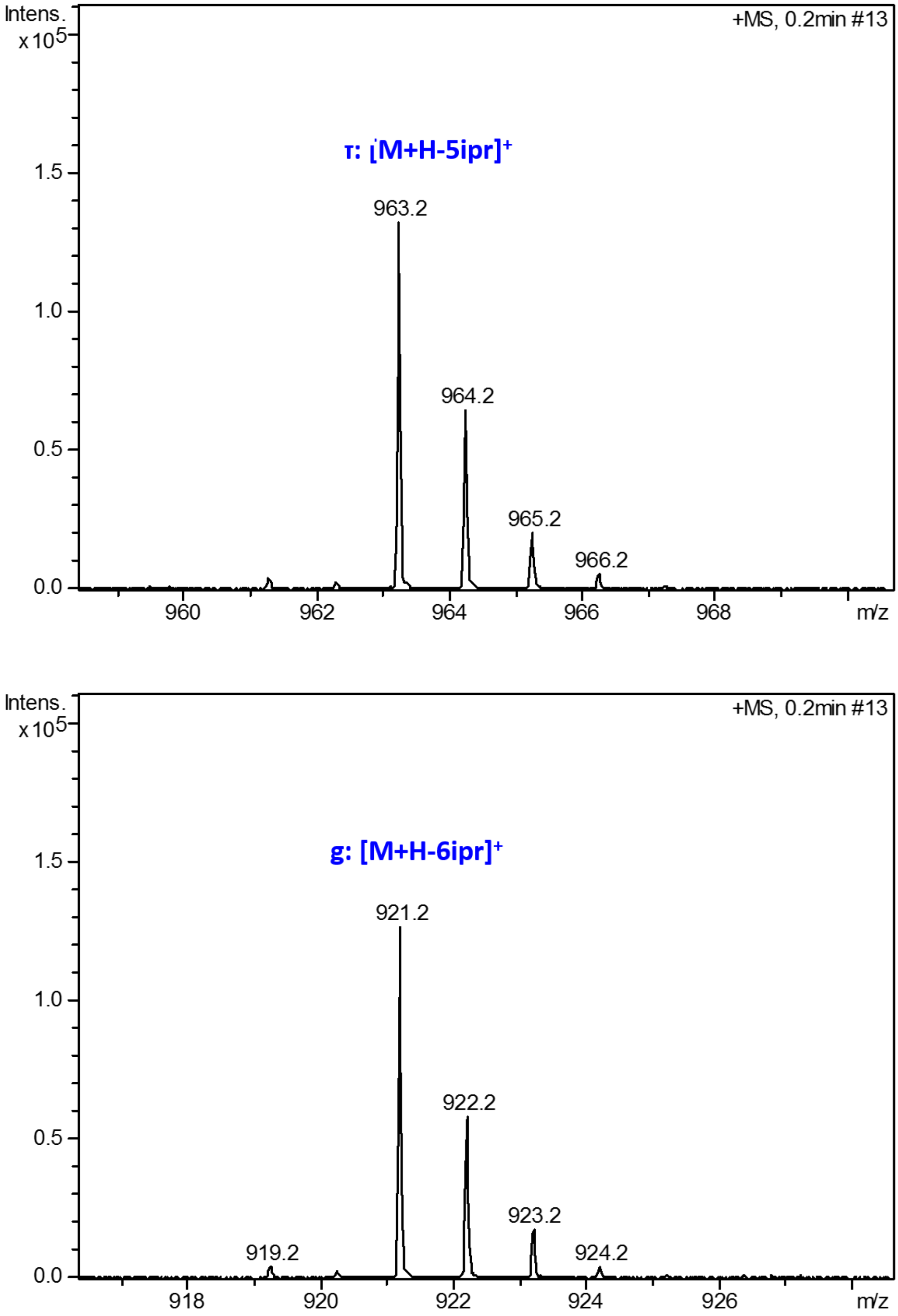

Figure S2. ESI MS spectrum of compound 19.

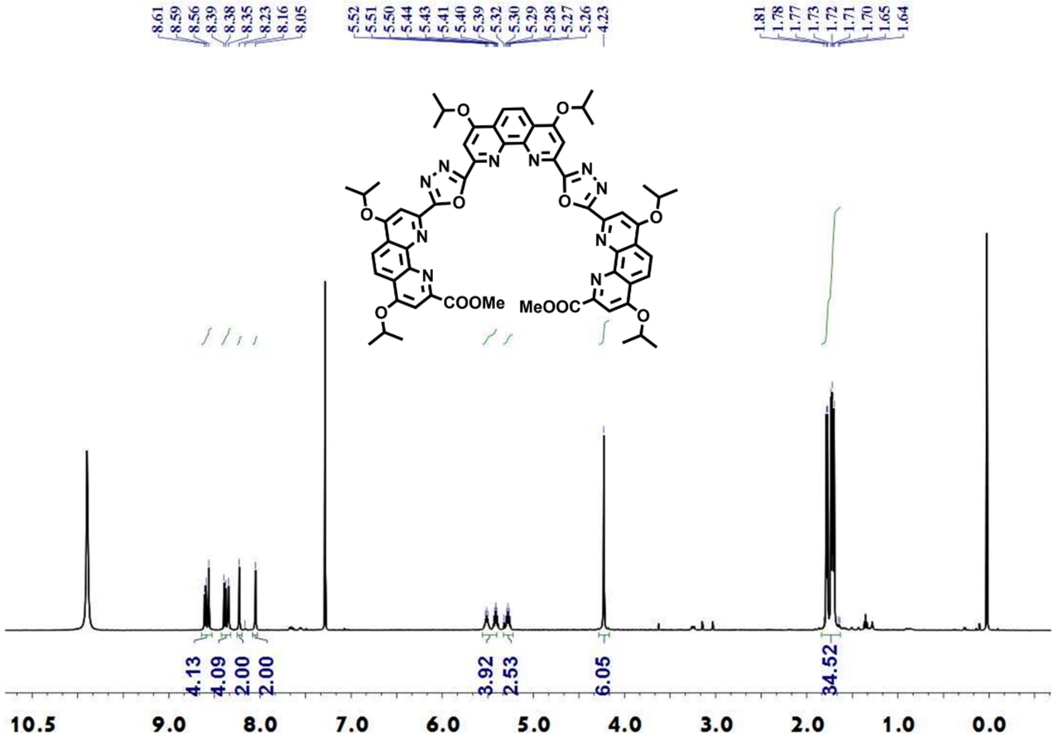

Figure S3. ^{1}H NMR spectrum of compound 20 in $CDCl_3$ ($CDCl_3$/ Trifluoroacetic acid-D 50:1).

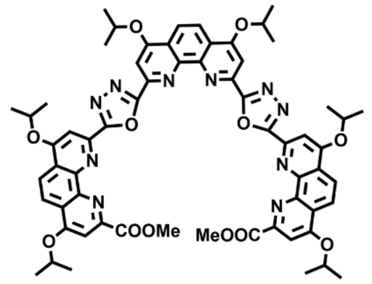

M_{15}=1136.4

ESI-full spectrum

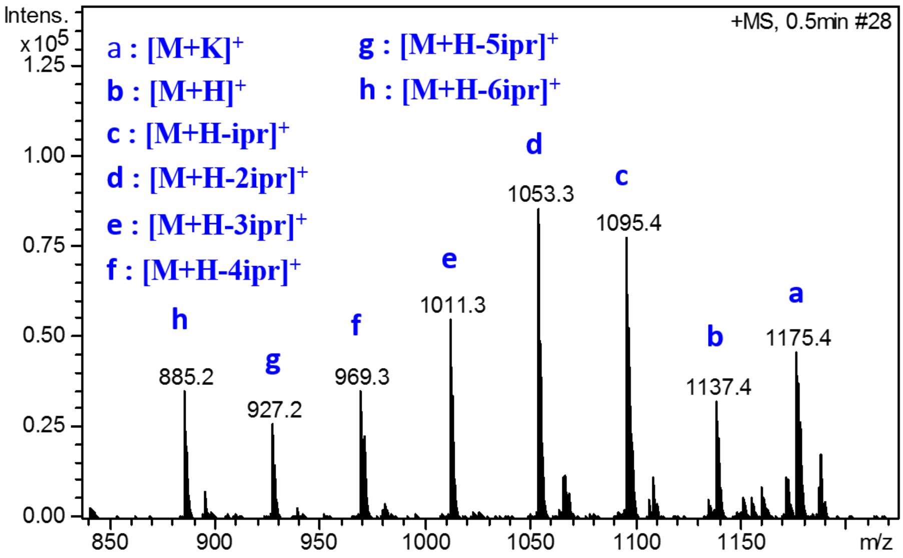

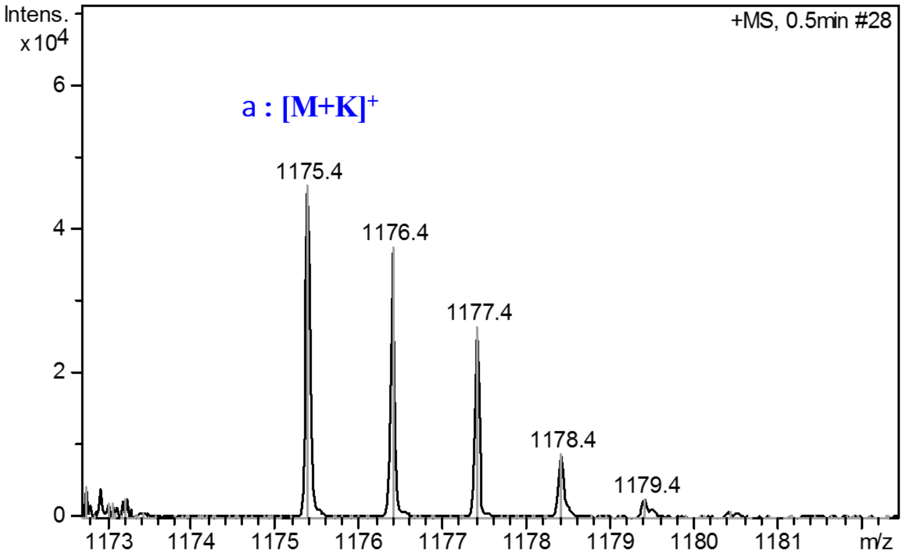

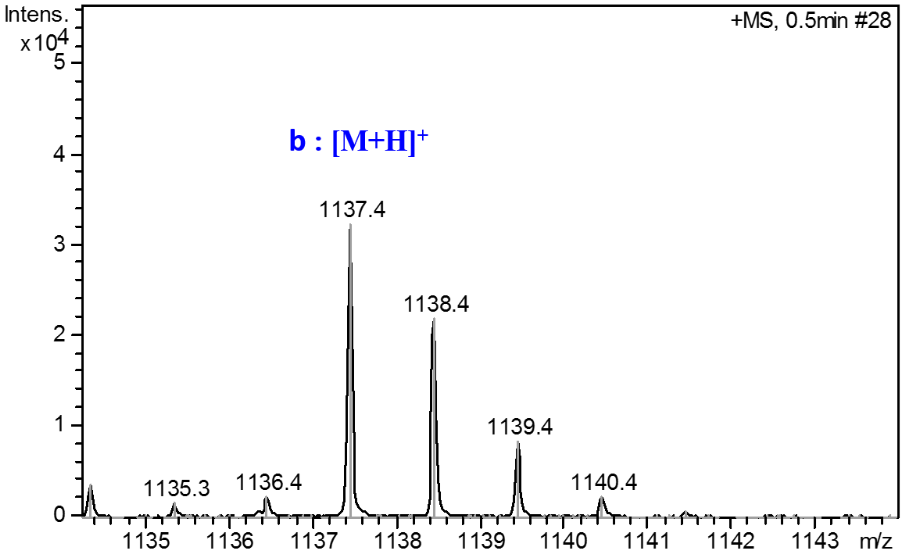

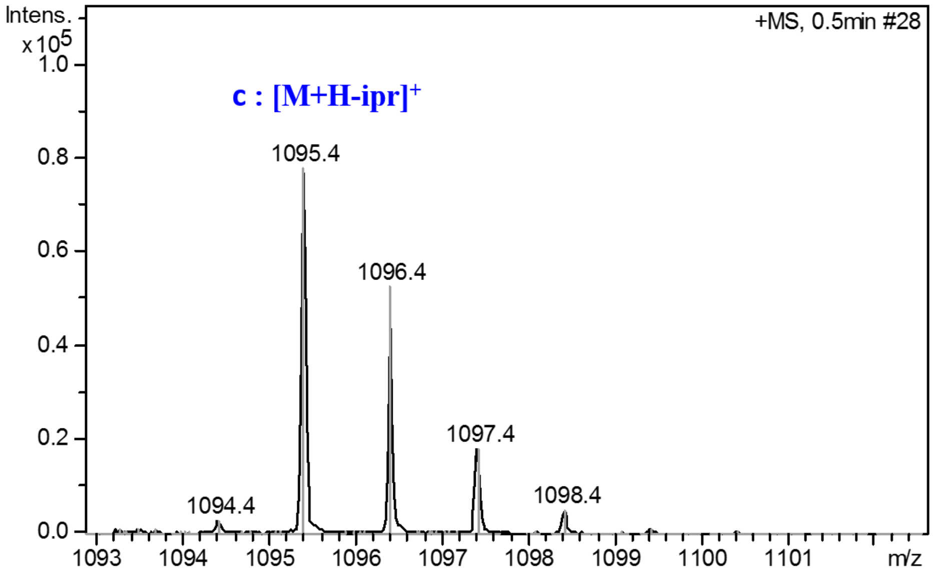
Intens.
x10^5
+MS, 0.5min #28
c : [M+H-ipr]+
1095.4
1096.4
1097.4
1094.4
1098.4
m/z

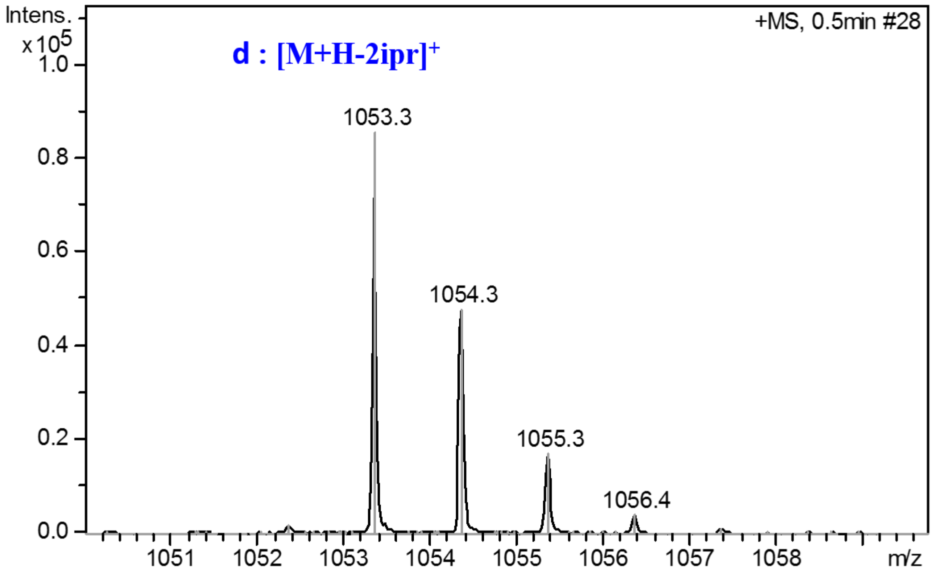
Intens.
x10^5
+MS, 0.5min #28
d : [M+H-2ipr]+
1053.3
1054.3
1055.3
1056.4
m/z

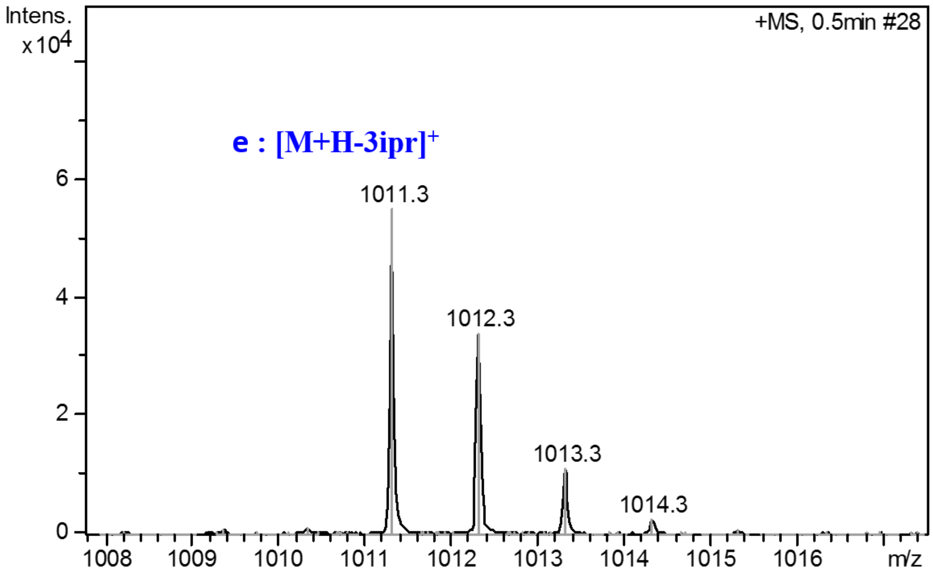
Intens.
x10^4
+MS, 0.5min #28
e : [M+H-3ipr]+
1011.3
1012.3
1013.3
1014.3
m/z

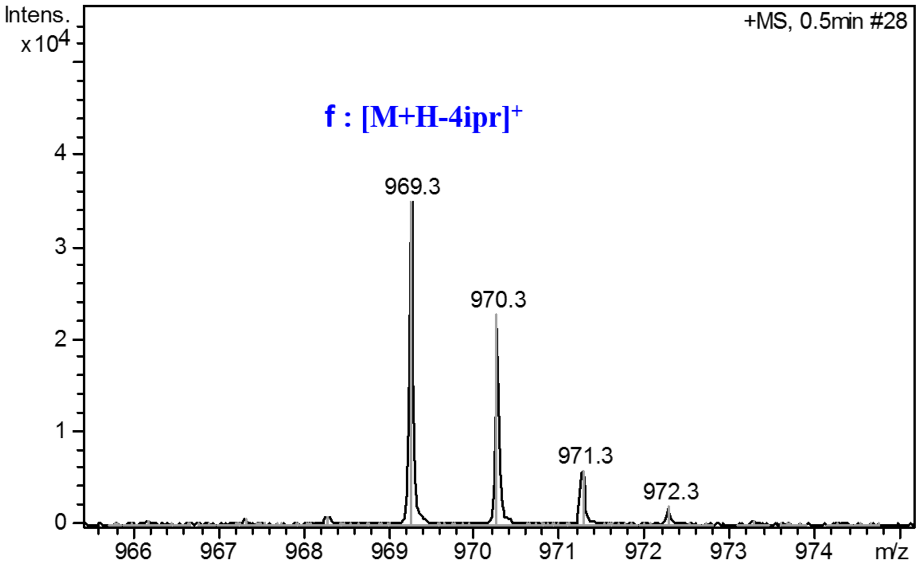

Intens.
x10^4
+MS, 0.5min #28
f : [M+H-4ipr]+
969.3
970.3
971.3
972.3
m/z

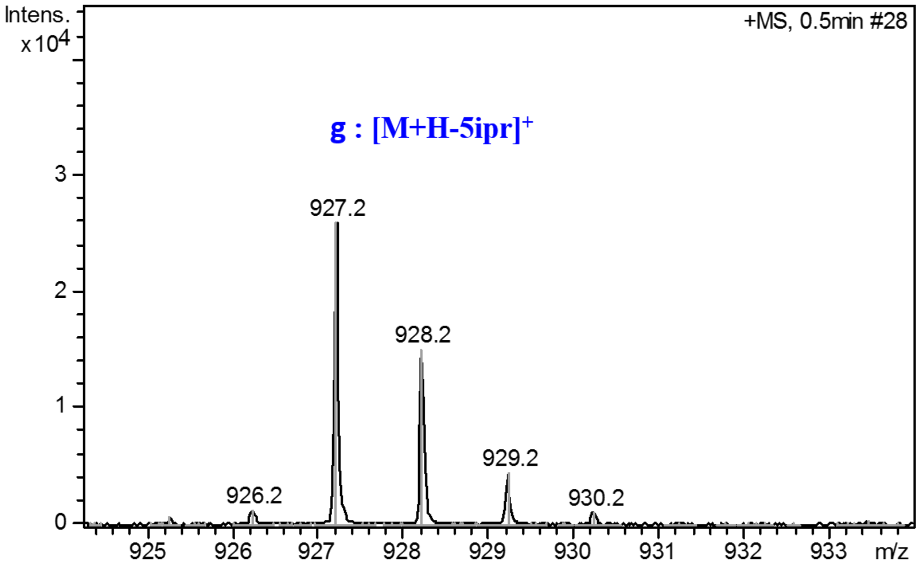

Intens.
x10^4
+MS, 0.5min #28
g : [M+H-5ipr]+
927.2
928.2
929.2
926.2
930.2
m/z

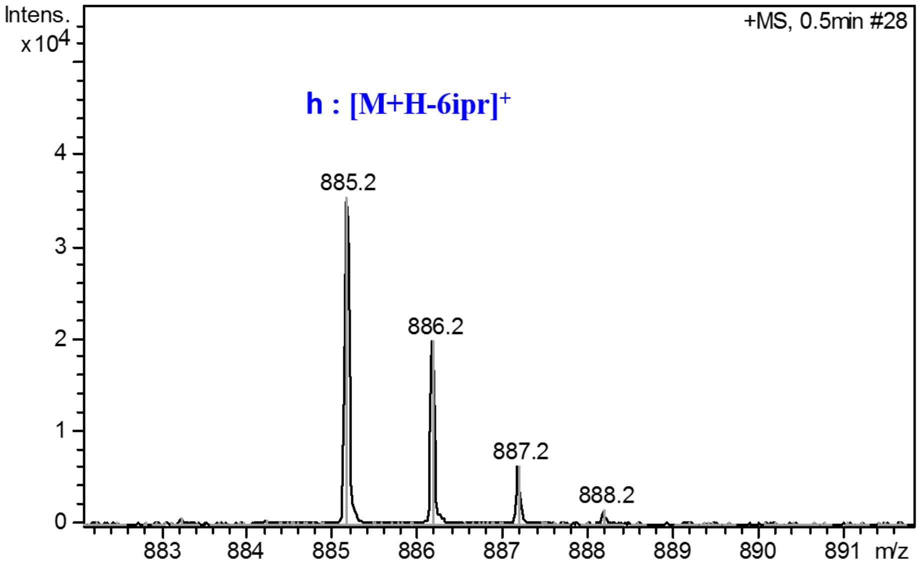

Intens.
x10^4
+MS, 0.5min #28
h : [M+H-6ipr]+
885.2
886.2
887.2
888.2
m/z

Figure S4. ESI MS spectrum of compound 20.

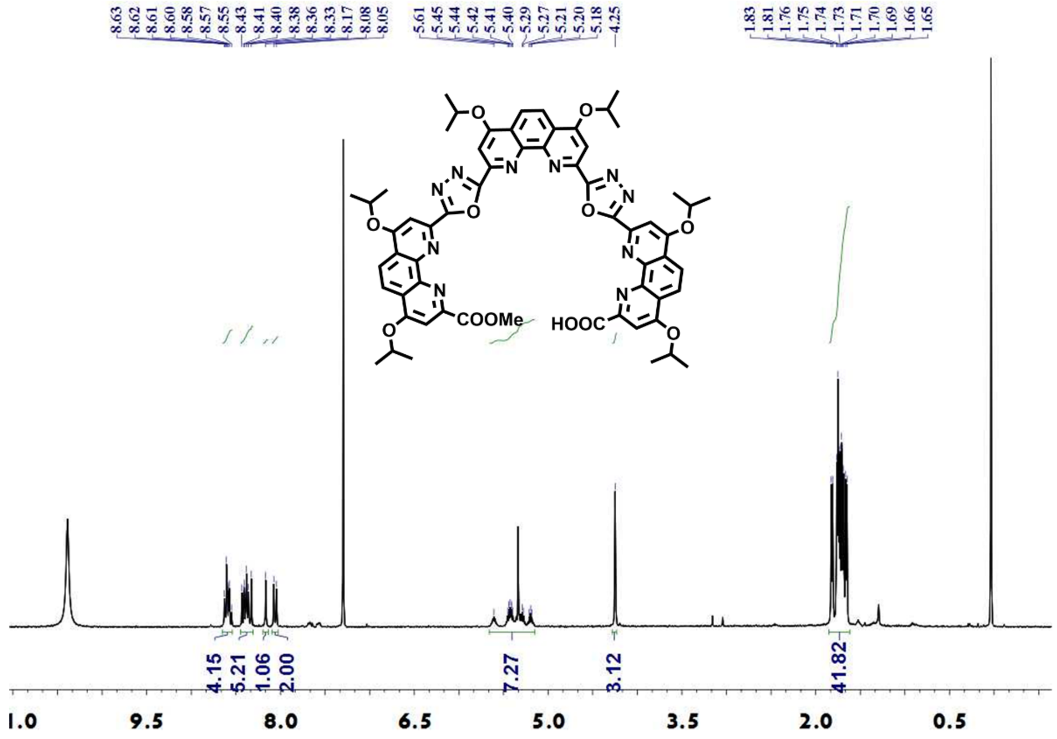

Figure S5. ^{1}H NMR spectrum of compound 9 in $CDCl_3$ ($CDCl_3$/ Trifluoroacetic acid-D 50:1).

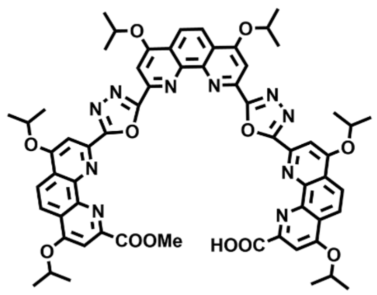

M_{16}=1122.4

ESI-full spectrum

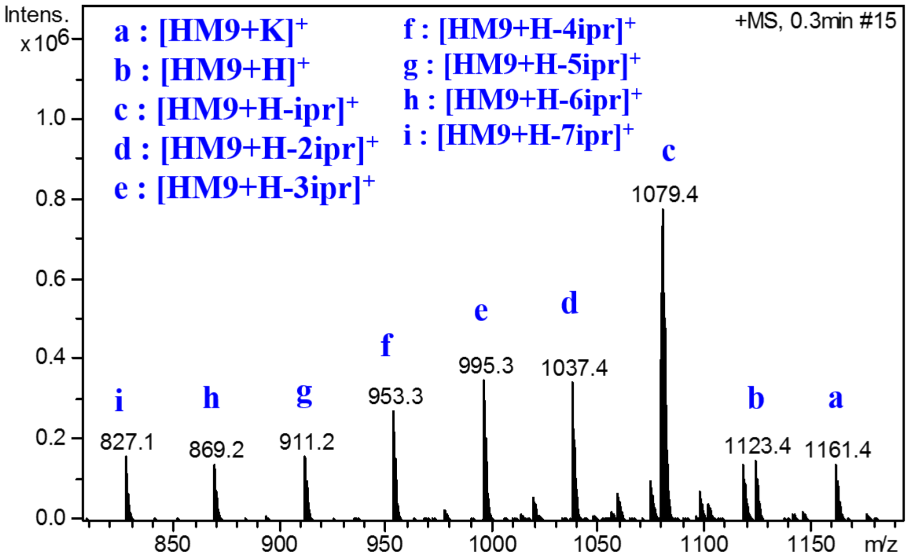

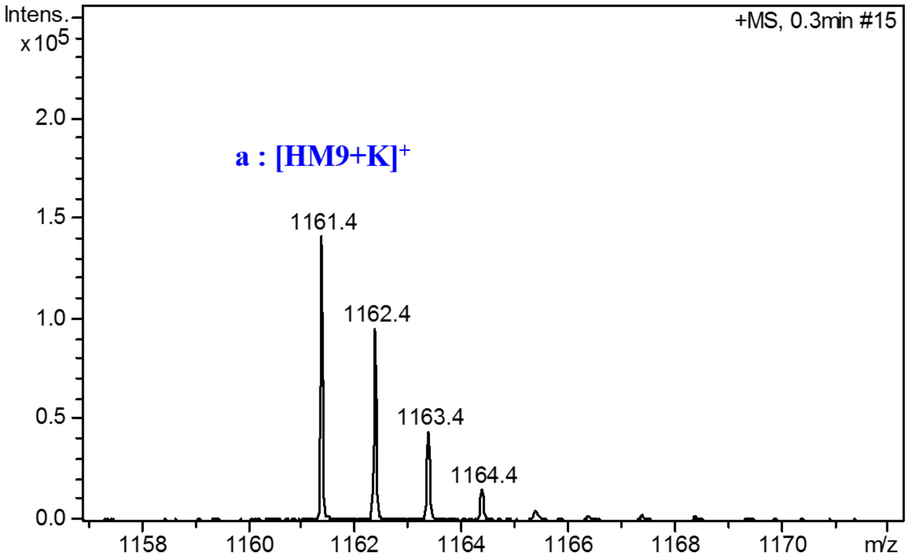

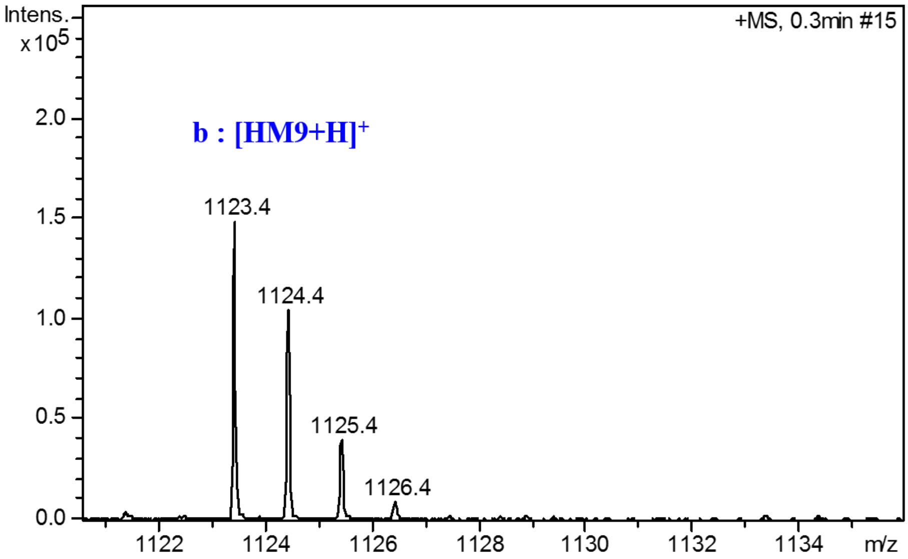

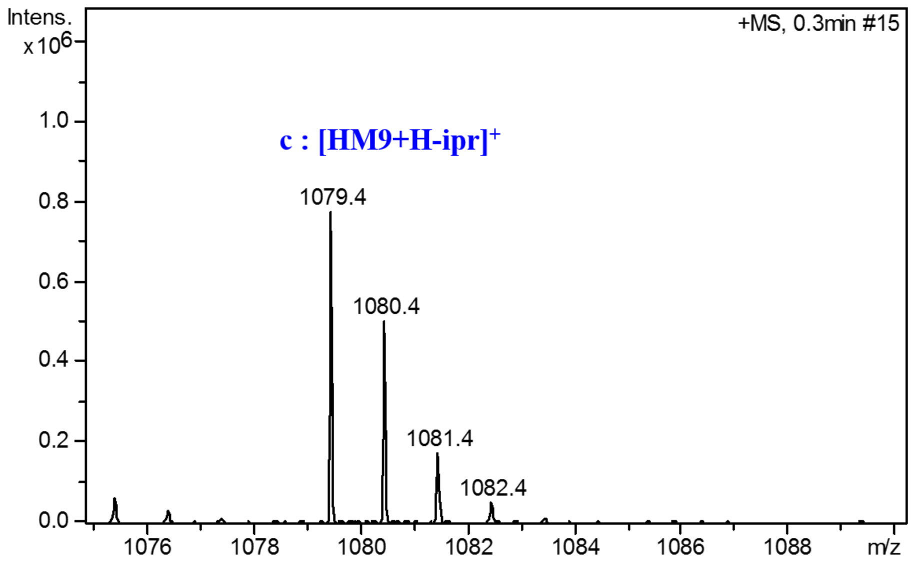
Intens.
x10^6
+MS, 0.3min #15
c : [HM9+H-ipr]+
1079.4
1080.4
1081.4
1082.4
m/z

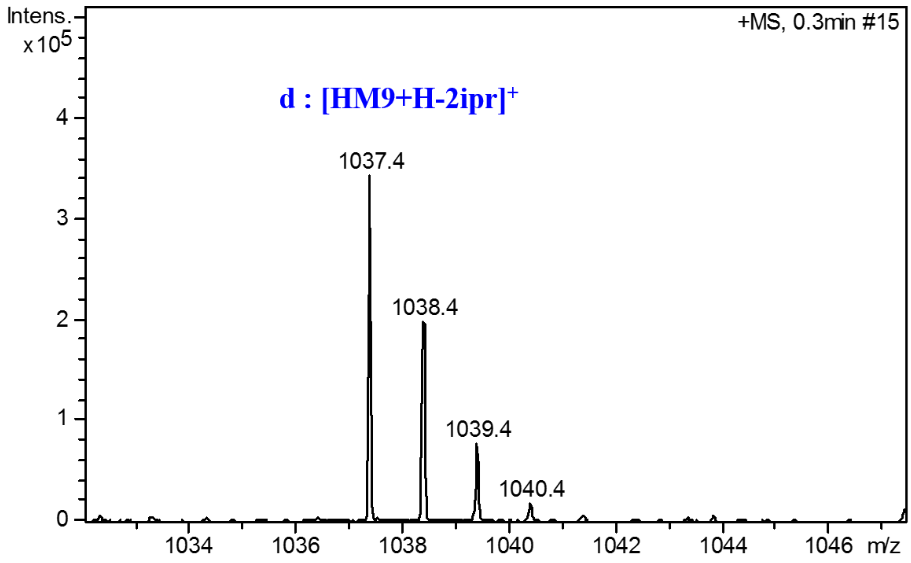
Intens.
x10^5
+MS, 0.3min #15
d : [HM9+H-2ipr]+
1037.4
1038.4
1039.4
1040.4
m/z

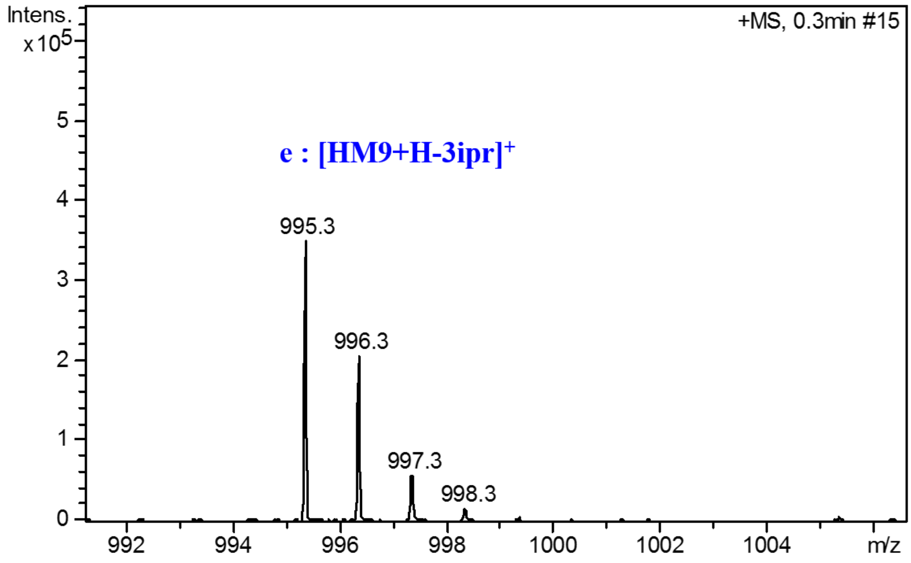
Intens.
x10^5
+MS, 0.3min #15
e : [HM9+H-3ipr]+
995.3
996.3
997.3
998.3
m/z

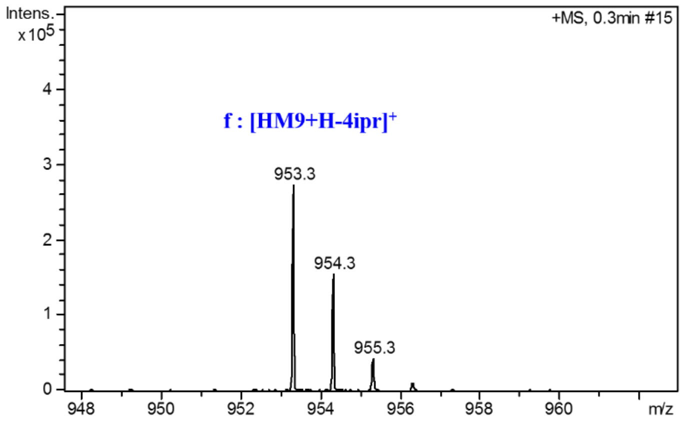
Intens.
x10^5
+MS, 0.3min #15
f : [HM9+H-4ipr]+
953.3
954.3
955.3
m/z

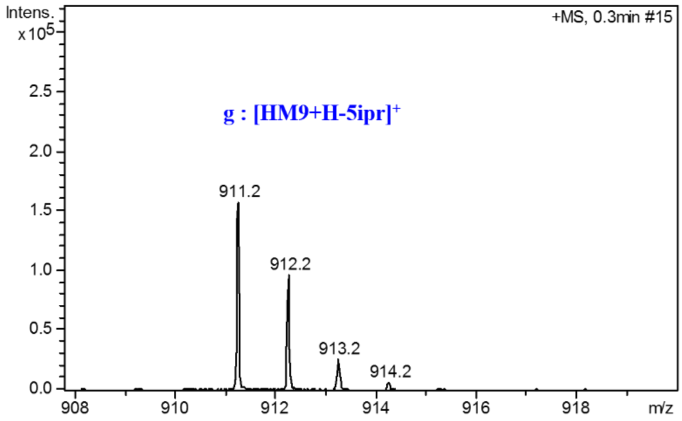
Intens.
x10^5
+MS, 0.3min #15
g : [HM9+H-5ipr]+
911.2
912.2
913.2
914.2
m/z

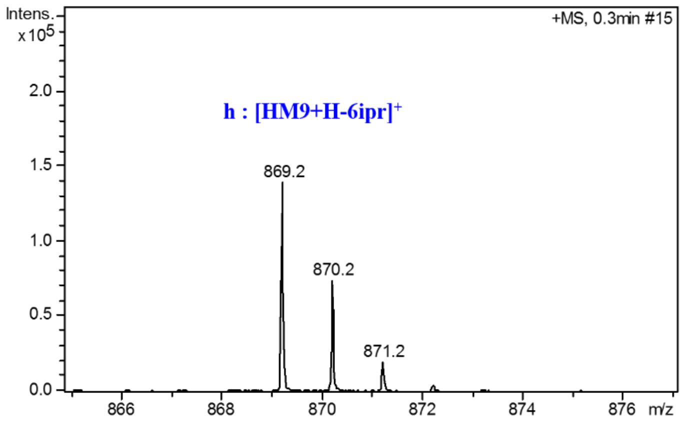
Intens.
x10^5
+MS, 0.3min #15
h : [HM9+H-6ipr]+
869.2
870.2
871.2
m/z

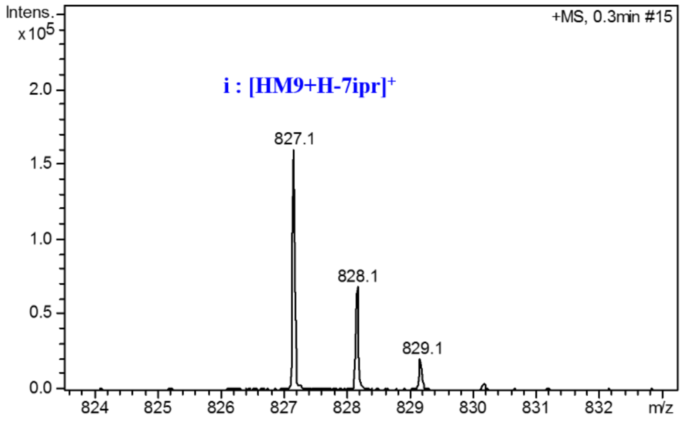
Intens.
x10^5
+MS, 0.3min #15
i : [HM9+H-7ipr]+
827.1
828.1
829.1
m/z

Figure S6. ESI MS spectrum of compound 9.

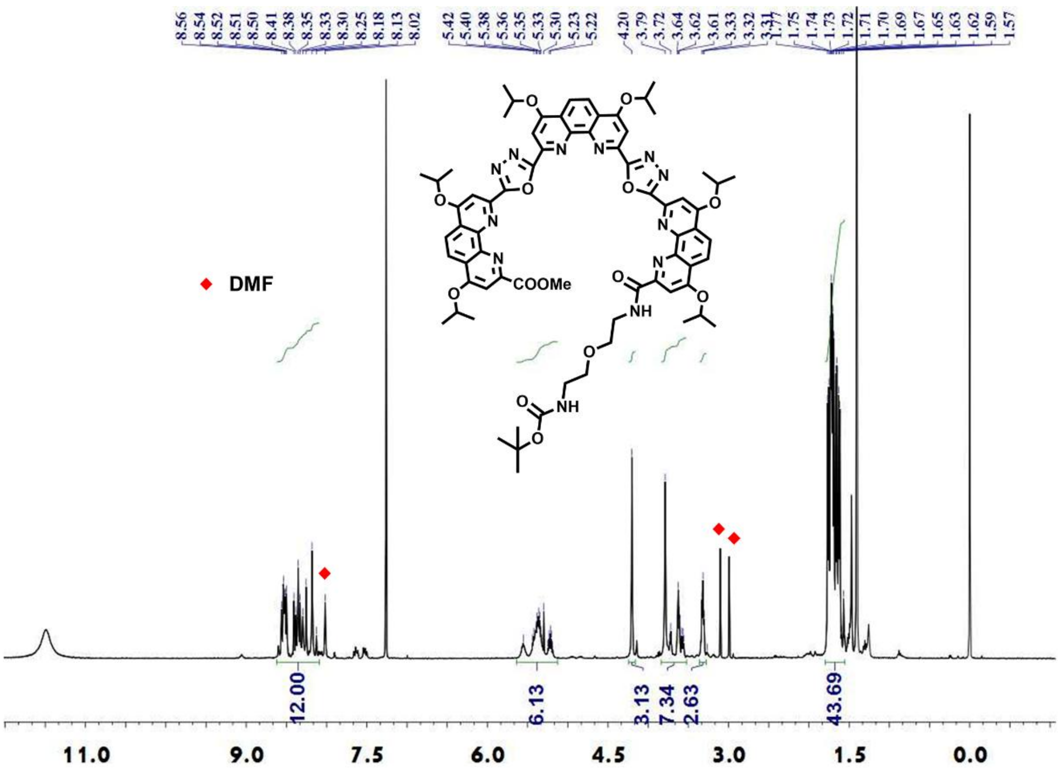

Figure S7. ^{1}H NMR spectrum of compound 6 in $CDCl_3$ ($CDCl_3$/ Trifluoroacetic acid-D 50:1).

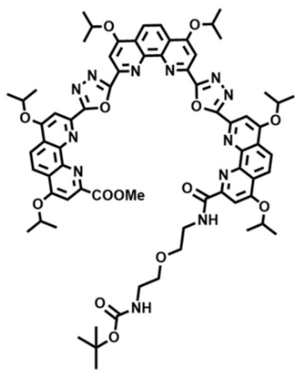

M_{18}=1308.5

ESI-full spectrum

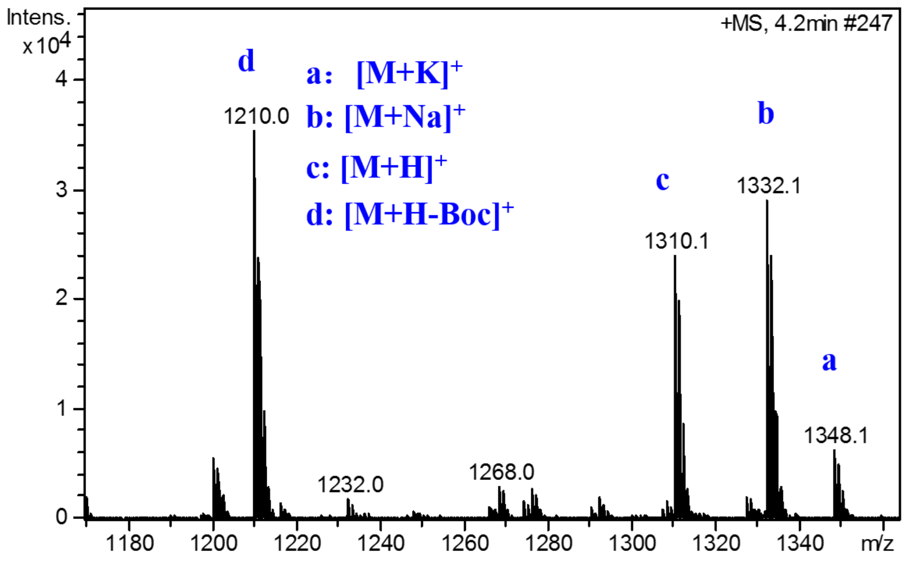

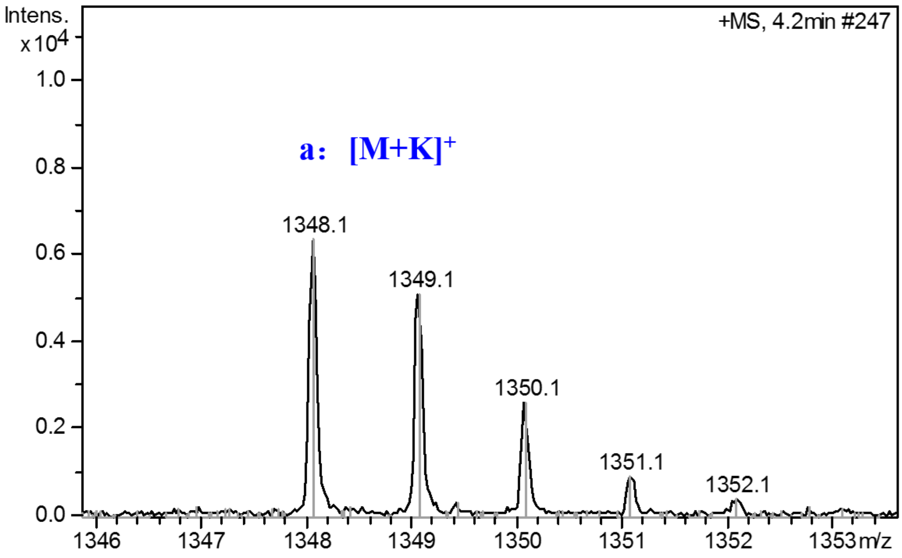

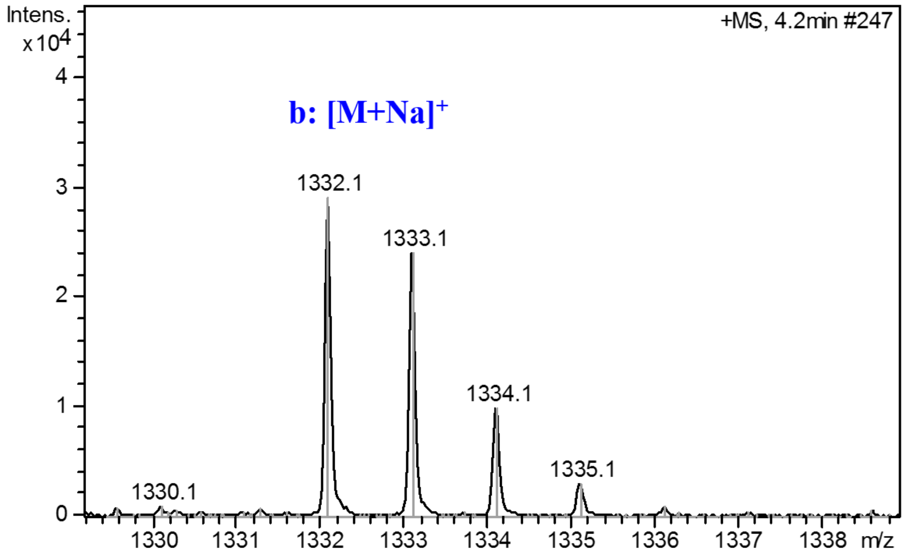

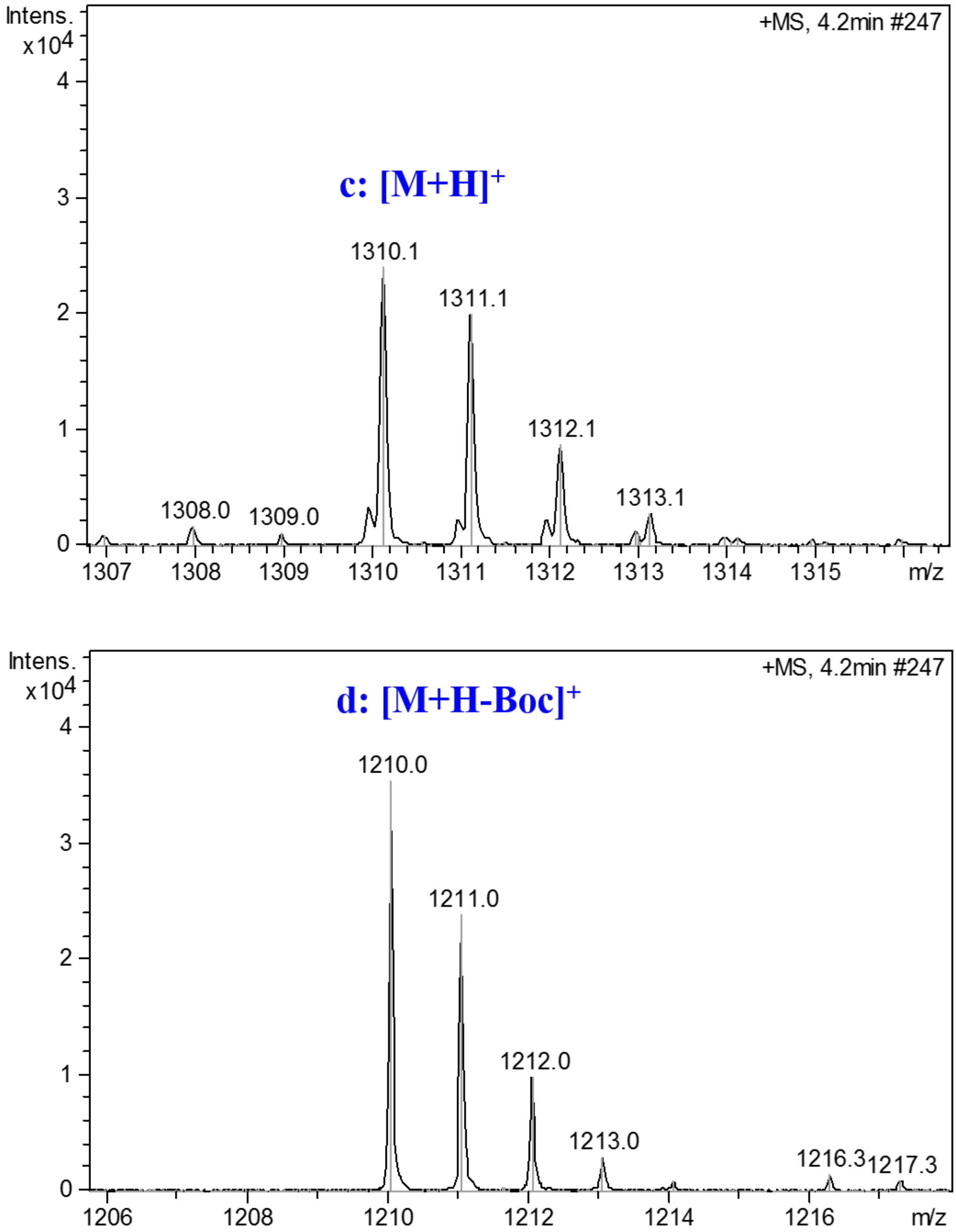

Figure S8. ESI MS spectrum of compound 6.

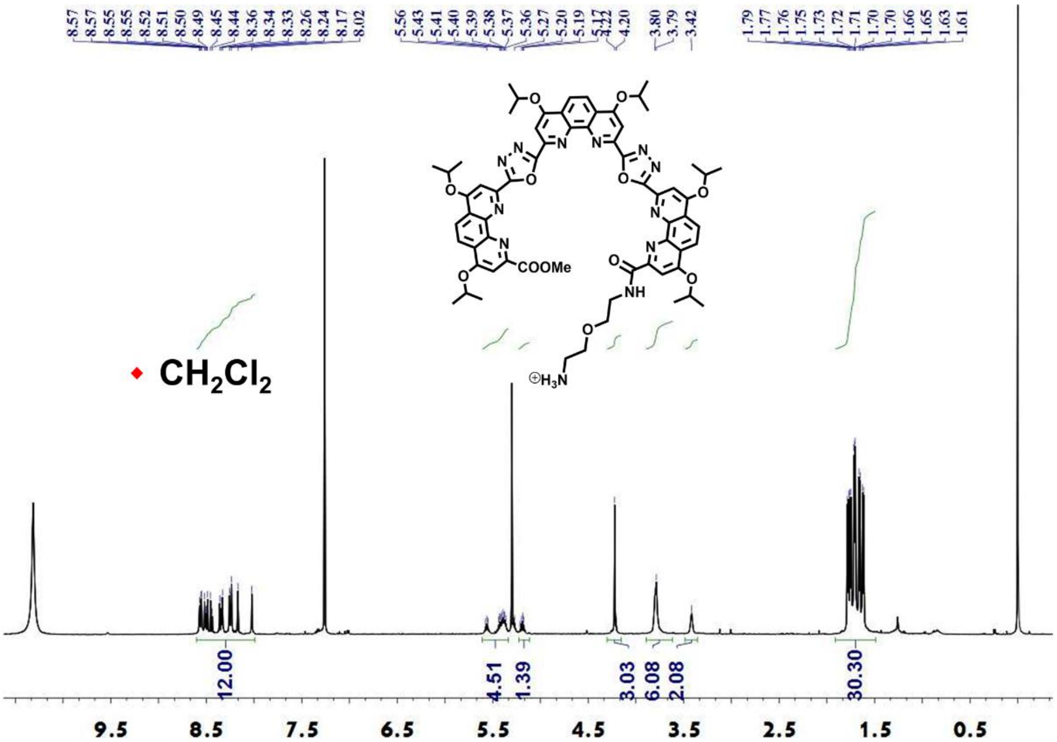

Figure S9. ^{1}H NMR spectrum of compound 21 in $CDCl_3$ ($CDCl_3$/ Trifluoroacetic acid-D 50:1).

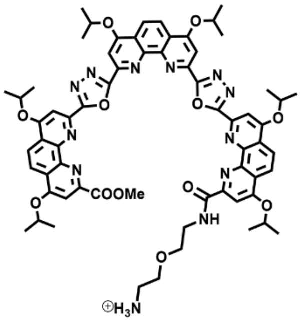

M_{19}=1208.5

ESI-full spectrum

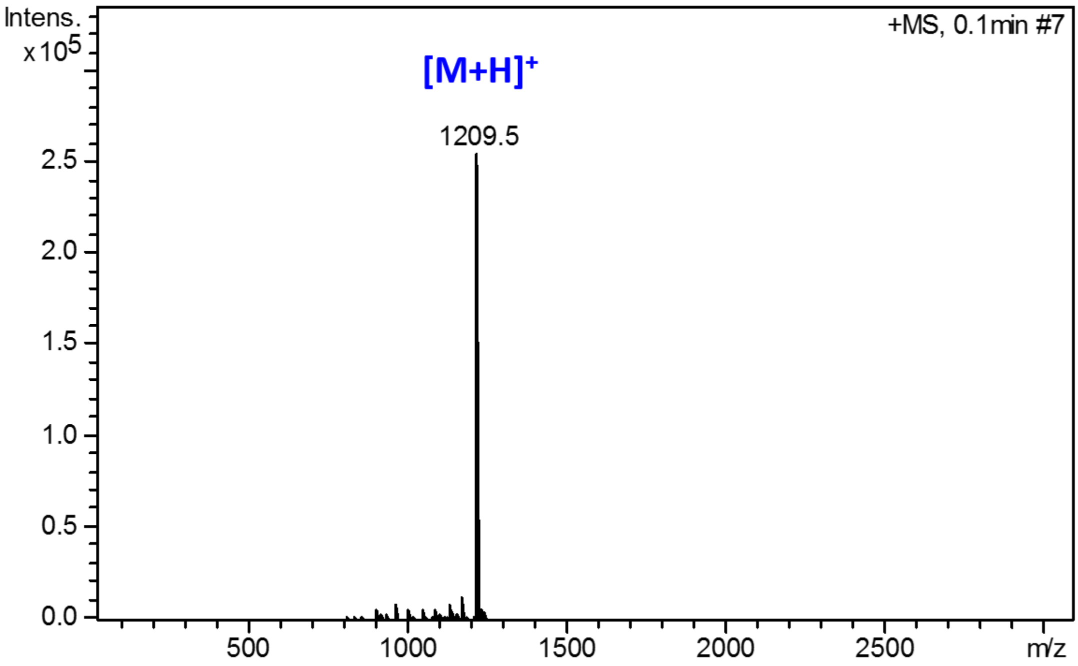

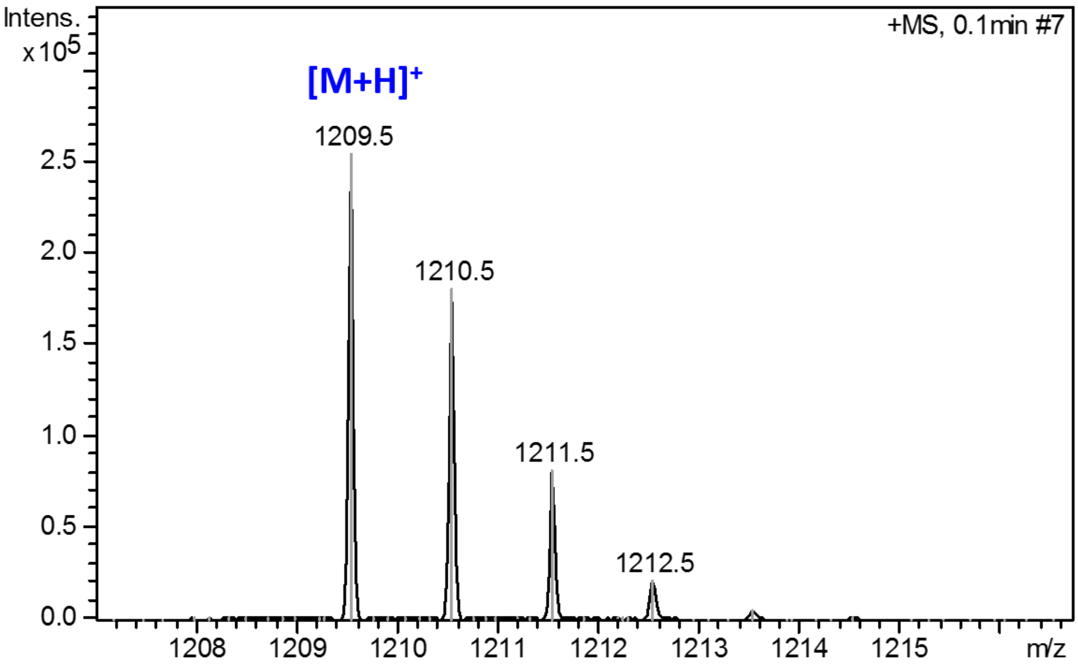

Figure S10. ESI MS spectrum of compound 21.

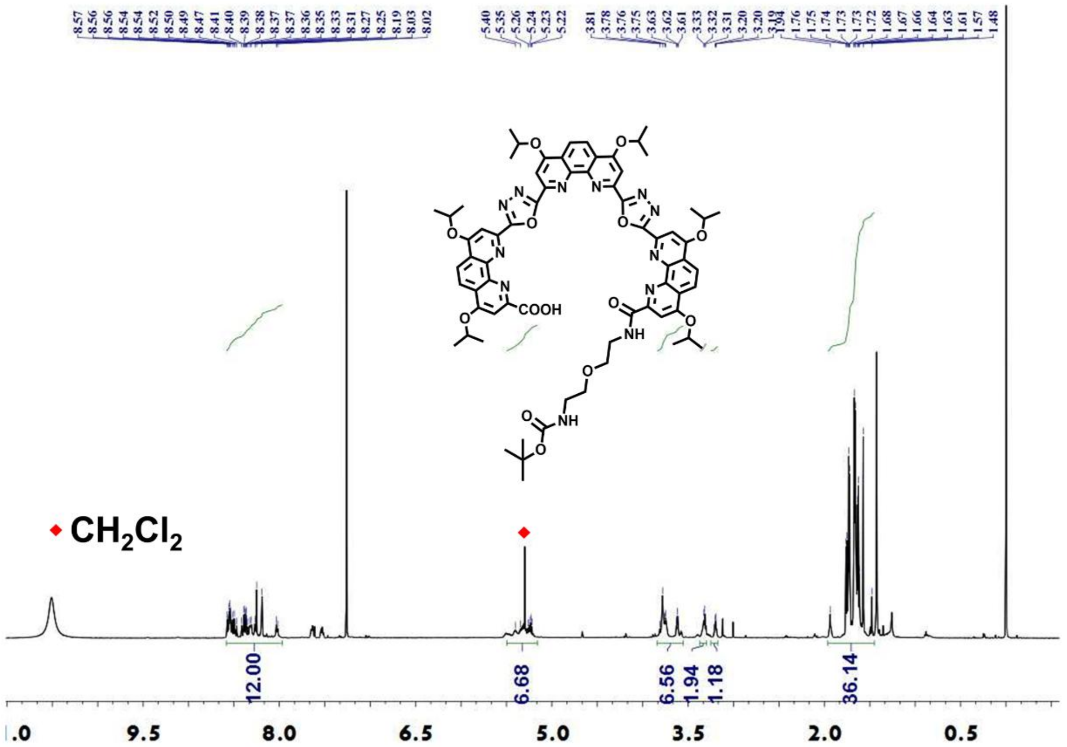

Figure S11. ^{1}H NMR spectrum of compound 22 in $CDCl_3$ ($CDCl_3$/ Trifluoroacetic acid-D 50:1).

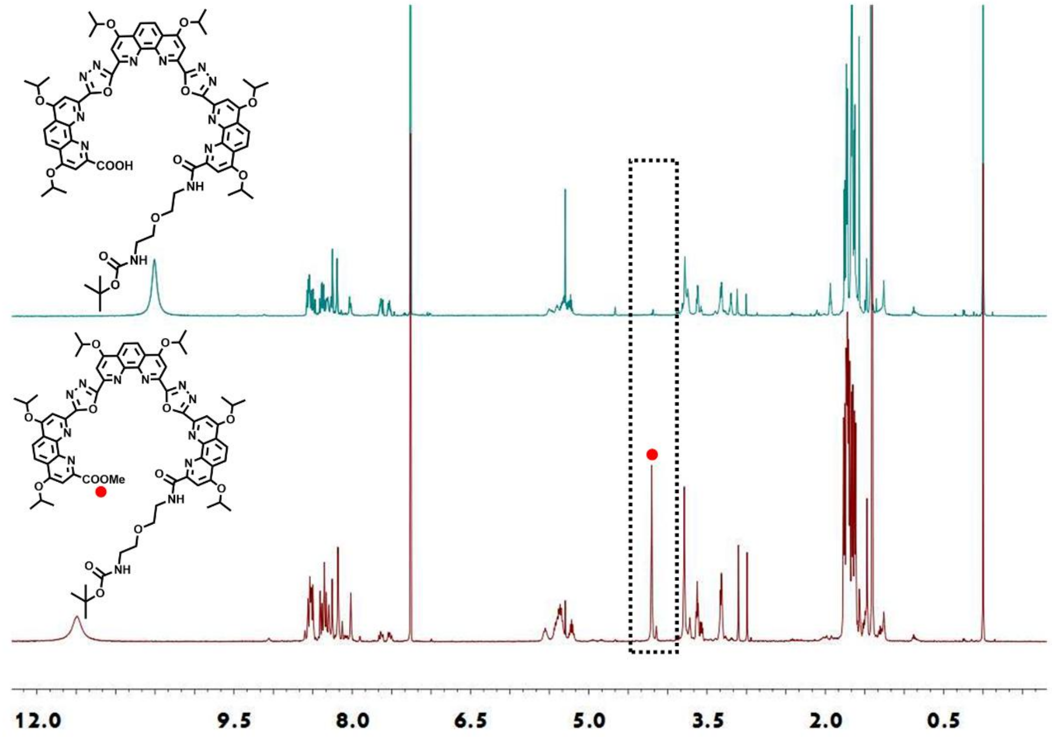

Figure S12. ^{1}H NMR spectrum of compound 6 and compound 22 in $CDCl_3$ ($CDCl_3$/ Trifluoroacetic acid-D 50:1).

ESI-full spectrum

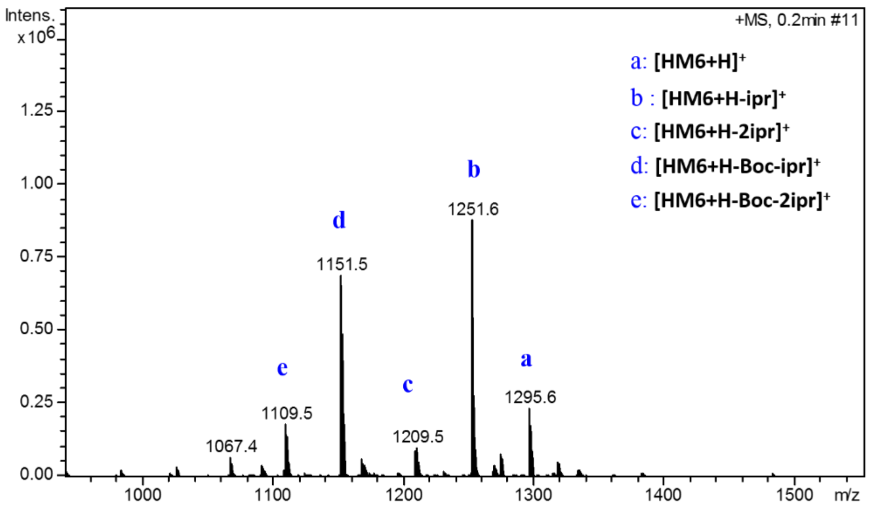

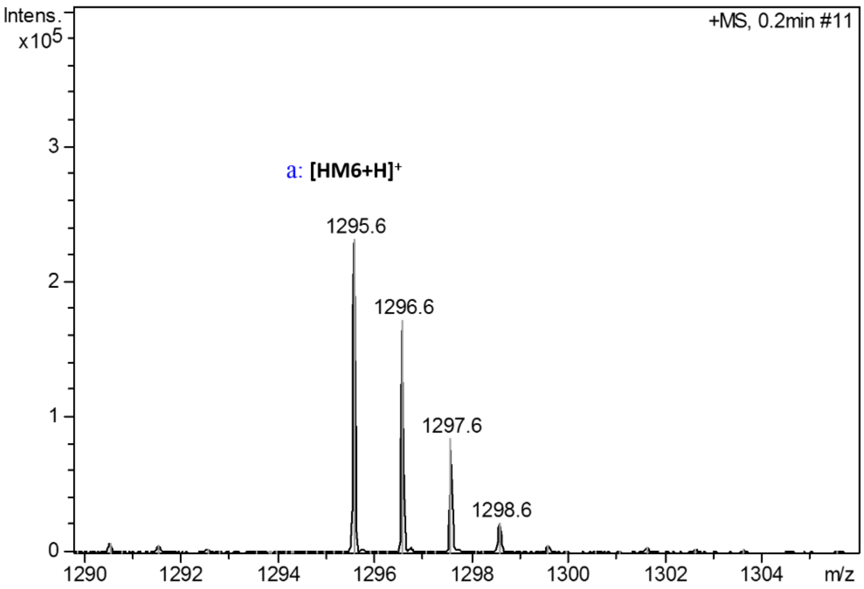

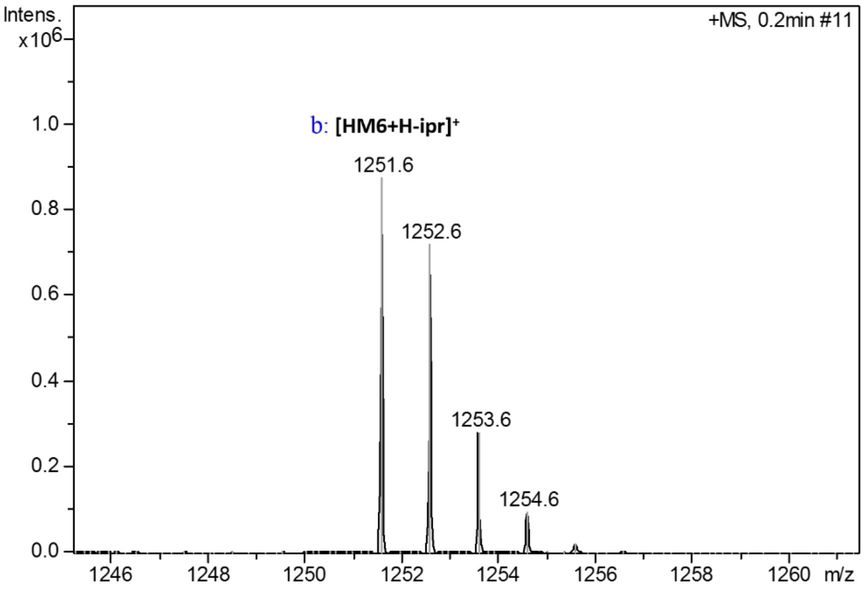

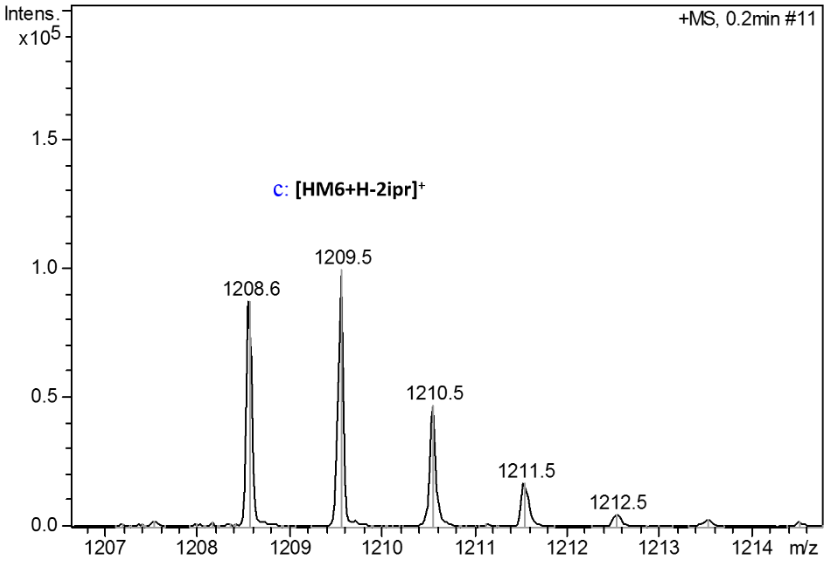

Intens.
x10^5
+MS, 0.2min #11
c: [HM6+H-2ipr]+
1208.6
1209.5
1210.5
1211.5
1212.5
m/z

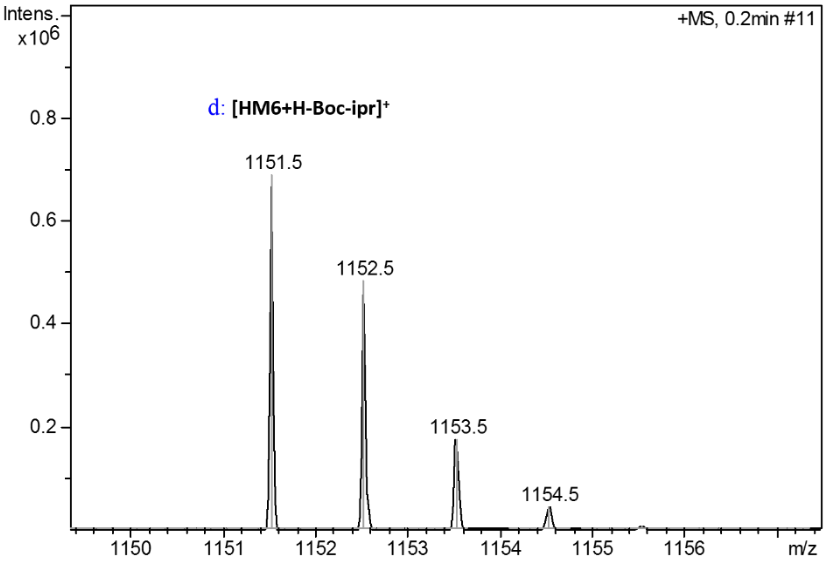

Intens.
x10^6
+MS, 0.2min #11
d: [HM6+H-Boc-ipr]+
1151.5
1152.5
1153.5
1154.5
m/z

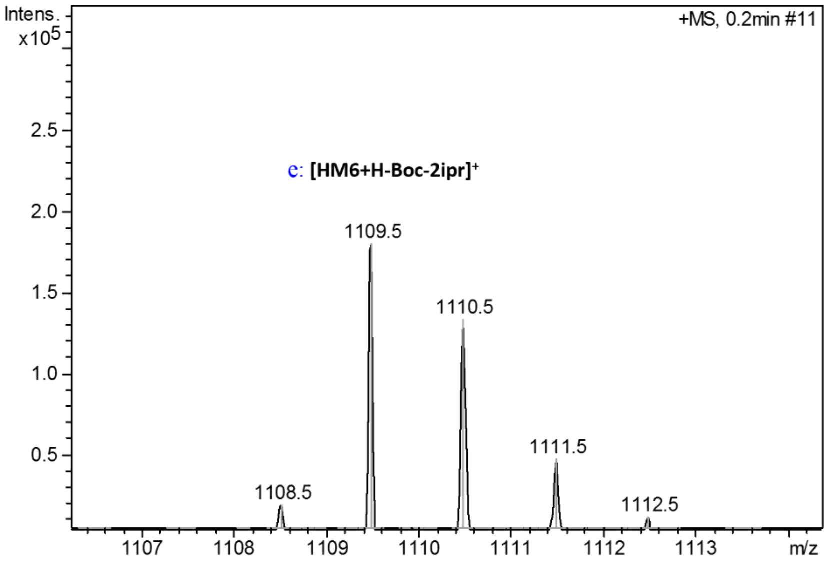

Intens.
x10^5
+MS, 0.2min #11
e: [HM6+H-Boc-2ipr]+
1108.5
1109.5
1110.5
1111.5
1112.5
m/z

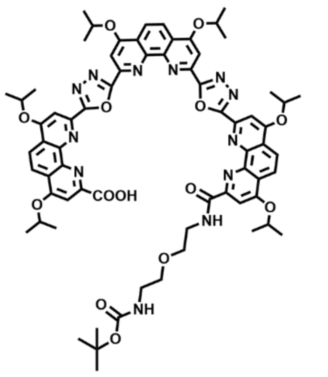

M20=1294.5

Figure S13. ESI MS spectrum of compound 22.

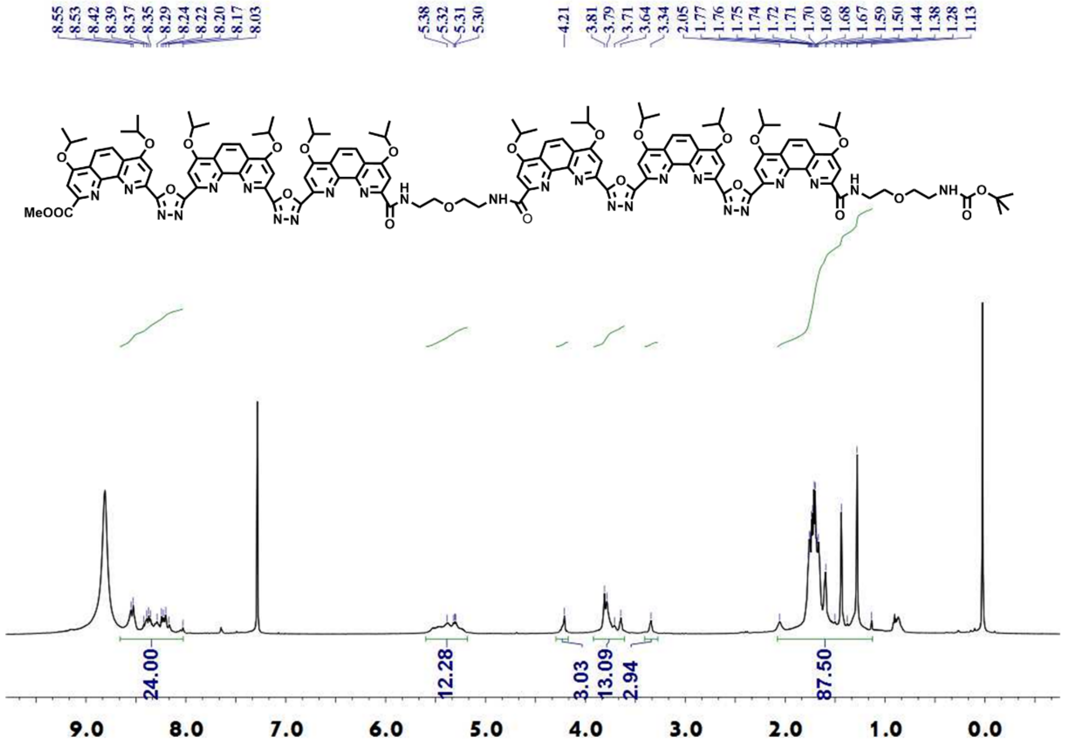

Figure S14. ^{1}H NMR spectrum of **HM1** in $CDCl_3$ ($CDCl_3$/ Trifluoroacetic acid-D 50:1).

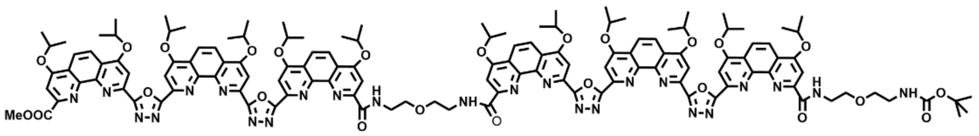

M1=2485.04

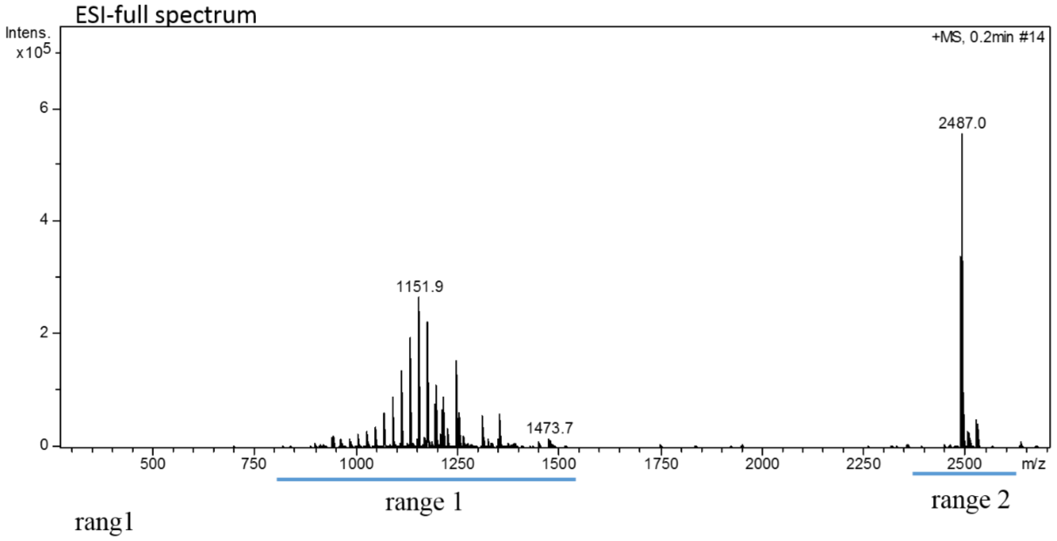

rang1

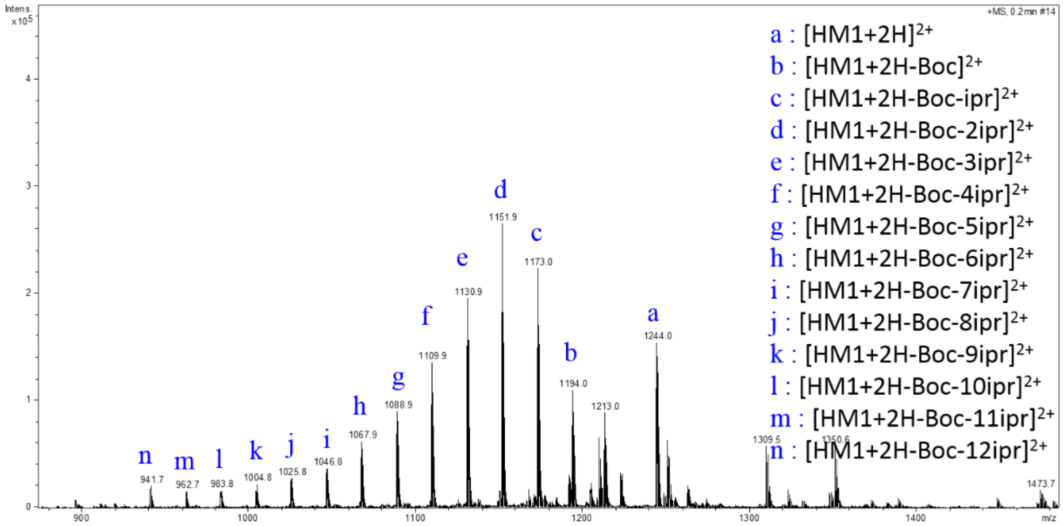

Range 2

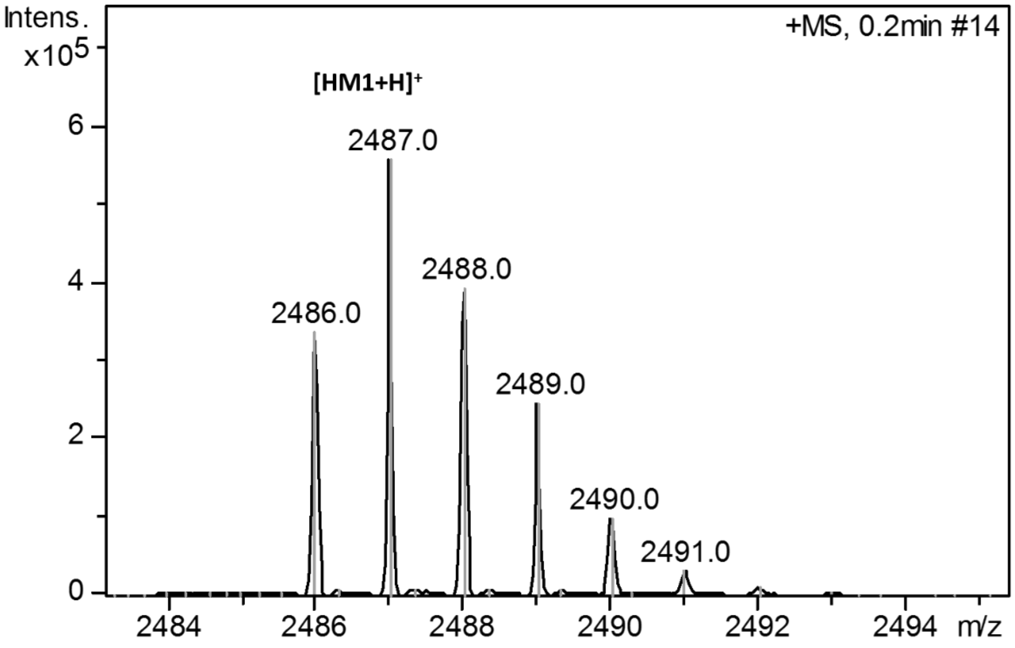

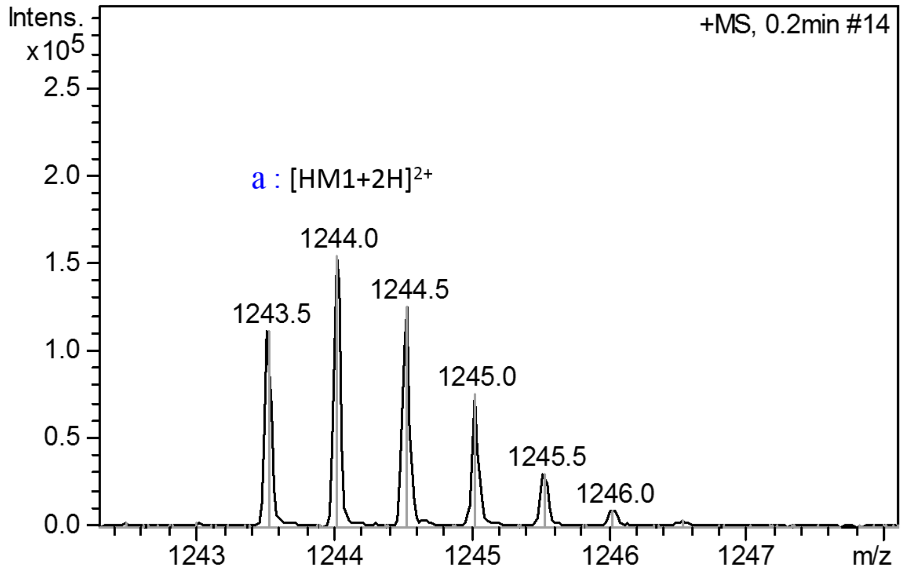
Intens.
x10^5
+MS, 0.2min #14
a : [HM1+2H]2+
1243.5
1244.0
1244.5
1245.0
1245.5
1246.0
m/z

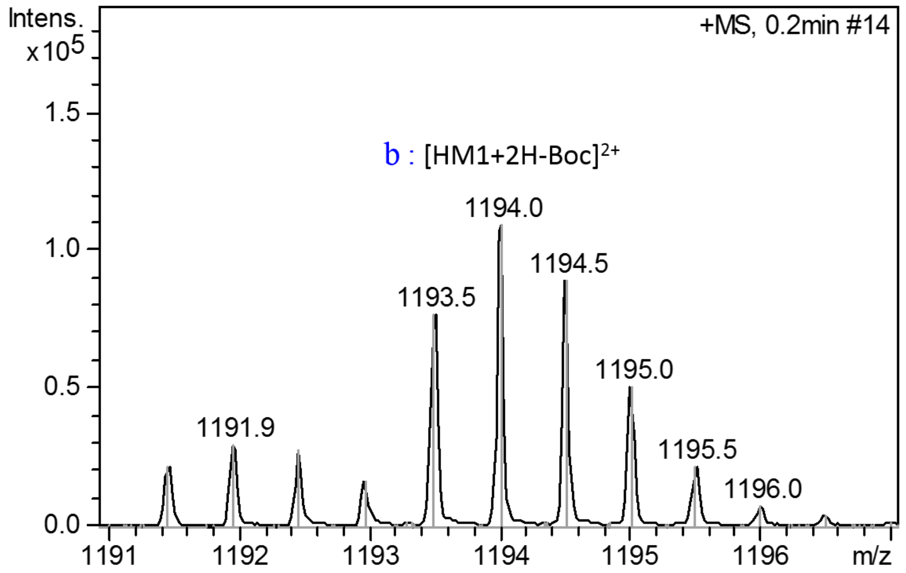
Intens.
x10^5
+MS, 0.2min #14
b : [HM1+2H-Boc]2+
1191.9
1193.5
1194.0
1194.5
1195.0
1195.5
1196.0
m/z

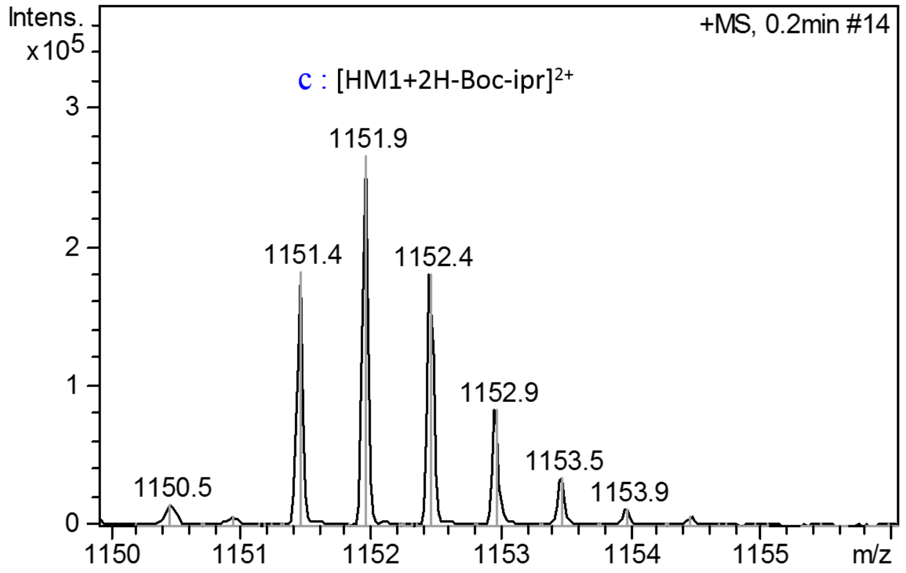
Intens.
x10^5
+MS, 0.2min #14
c : [HM1+2H-Boc-ipr]2+
1150.5
1151.4
1151.9
1152.4
1152.9
1153.5
1153.9
m/z

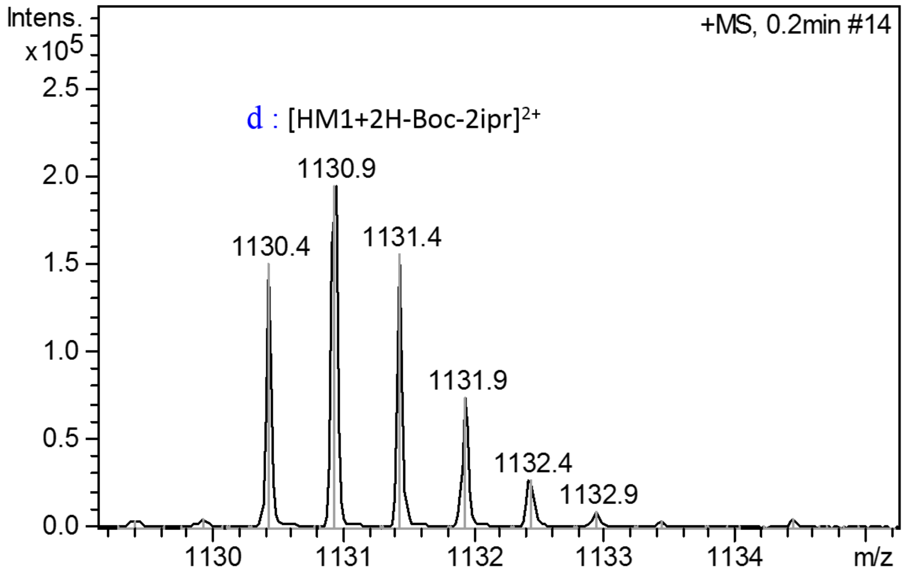
Intens.
x10^5
+MS, 0.2min #14
d : [HM1+2H-Boc-2ipr]2+
1130.4
1130.9
1131.4
1131.9
1132.4
1132.9
2.5
2.0
1.5
1.0
0.5
0.0
1130
1131
1132
1133
1134
m/z

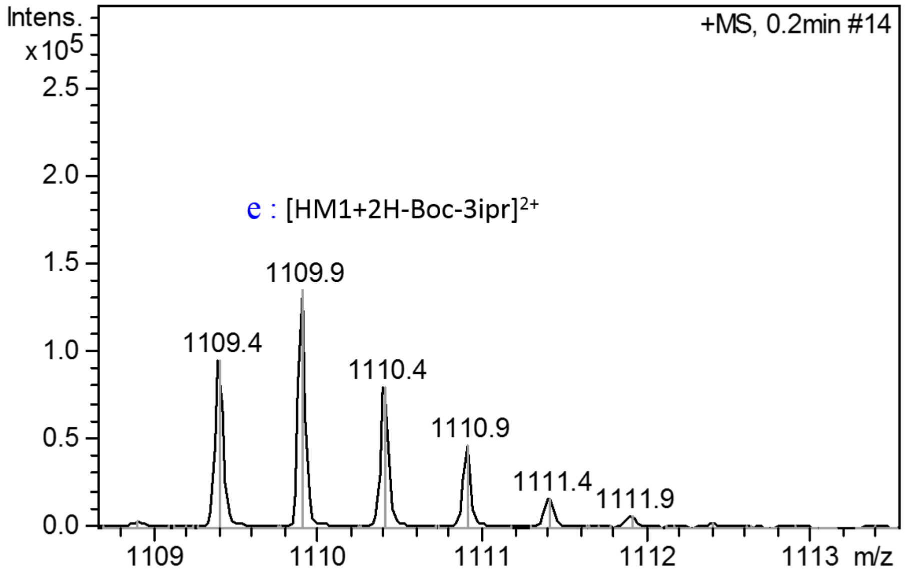
Intens.
x10^5
+MS, 0.2min #14
e : [HM1+2H-Boc-3ipr]2+
1109.4
1109.9
1110.4
1110.9
1111.4
1111.9
2.5
2.0
1.5
1.0
0.5
0.0
1109
1110
1111
1112
1113
m/z

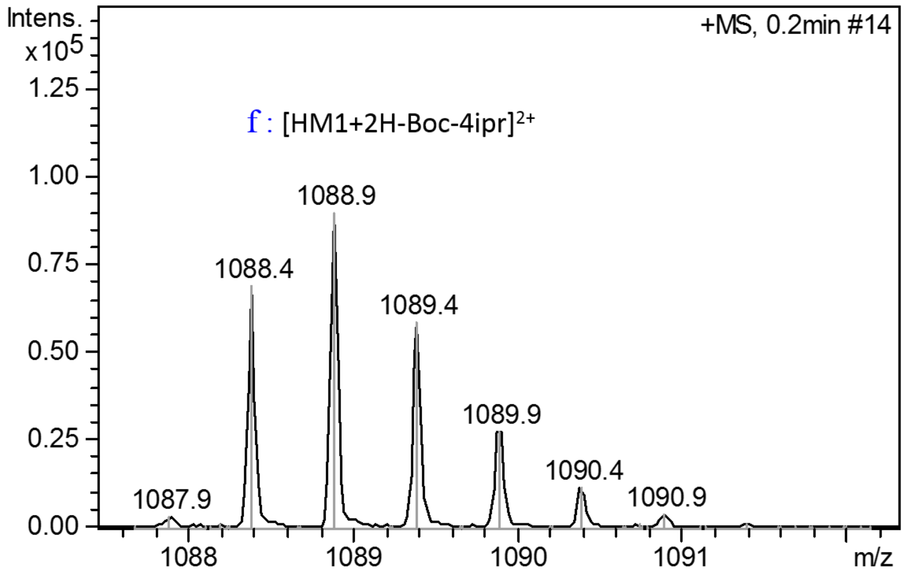
Intens.
x10^5
+MS, 0.2min #14
f : [HM1+2H-Boc-4ipr]2+
1087.9
1088.4
1088.9
1089.4
1089.9
1090.4
1090.9
1.25
1.00
0.75
0.50
0.25
0.00
1088
1089
1090
1091
m/z

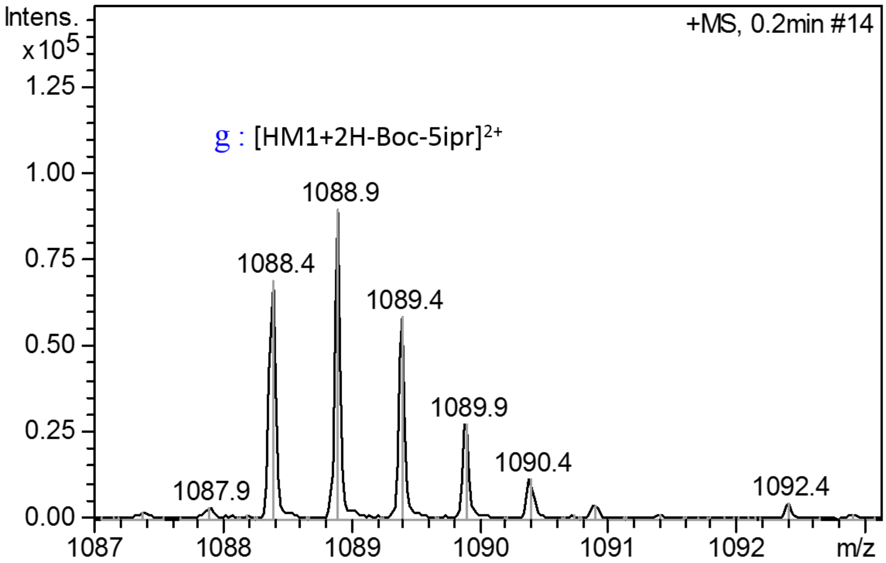
Intens.
x10^5
+MS, 0.2min #14
g : [HM1+2H-Boc-5ipr]2+
1087.9
1088.4
1088.9
1089.4
1089.9
1090.4
1092.4
m/z

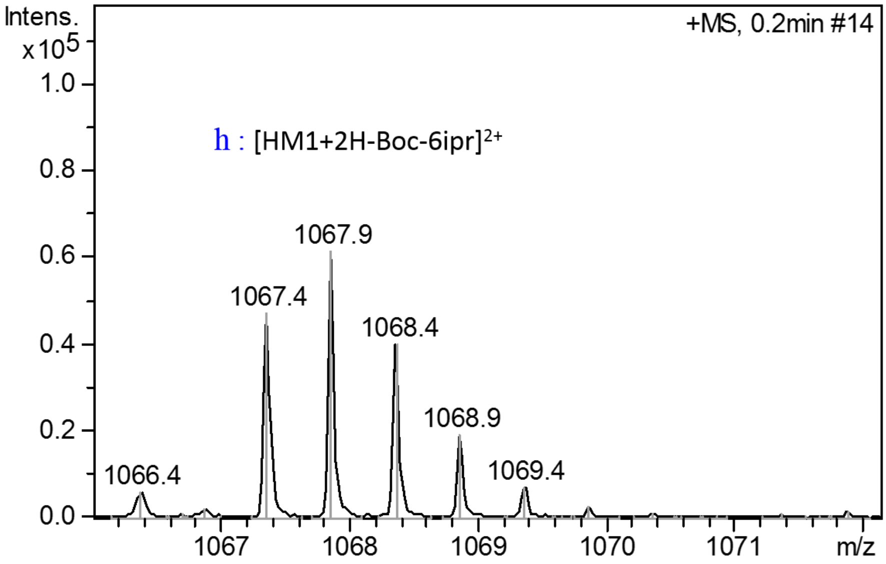
Intens.
x10^5
+MS, 0.2min #14
h : [HM1+2H-Boc-6ipr]2+
1066.4
1067.4
1067.9
1068.4
1068.9
1069.4
m/z

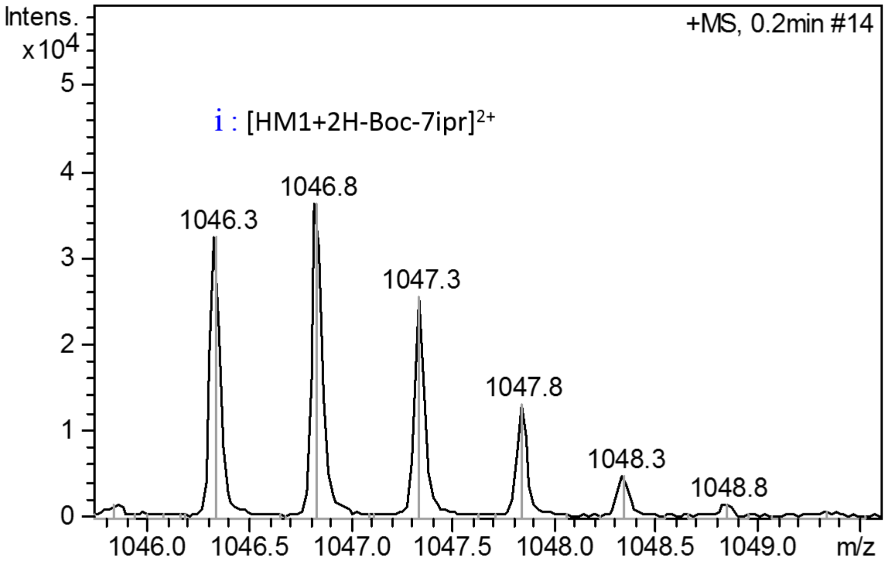
Intens.
x10^4
+MS, 0.2min #14
i : [HM1+2H-Boc-7ipr]2+
1046.3
1046.8
1047.3
1047.8
1048.3
1048.8
m/z

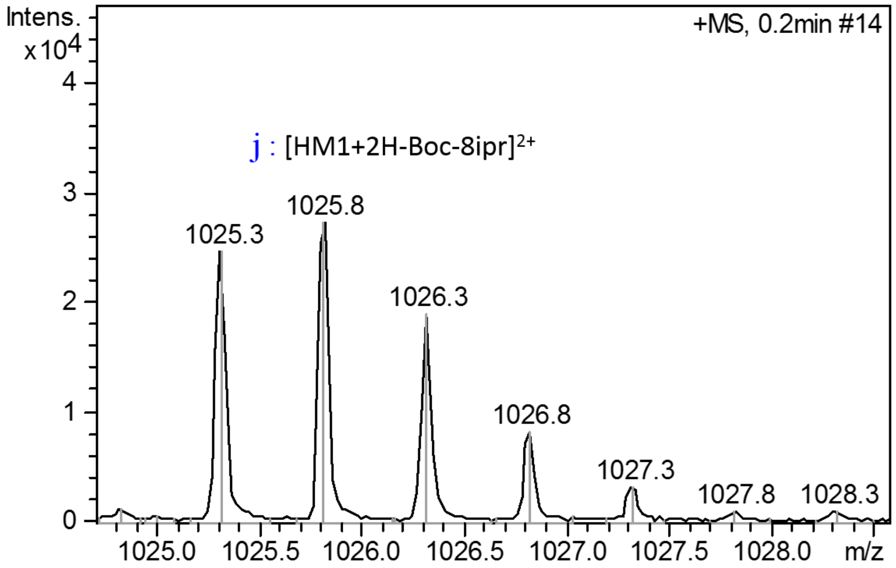
Intens.
x10^4
+MS, 0.2min #14
j : [HM1+2H-Boc-8ipr]2+
1025.3
1025.8
1026.3
1026.8
1027.3
1027.8
1028.3
m/z

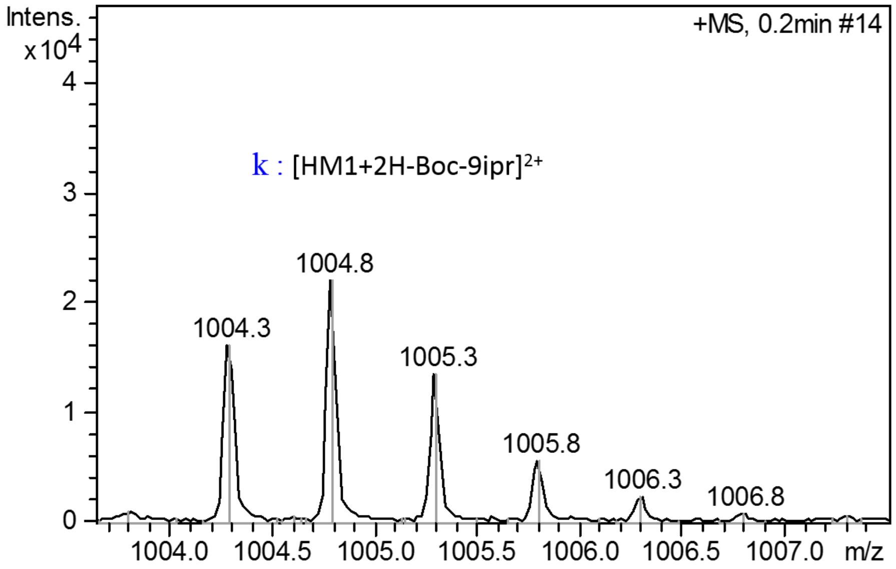
Intens.
x10^4
+MS, 0.2min #14
k : [HM1+2H-Boc-9ipr]2+
1004.3
1004.8
1005.3
1005.8
1006.3
1006.8
m/z

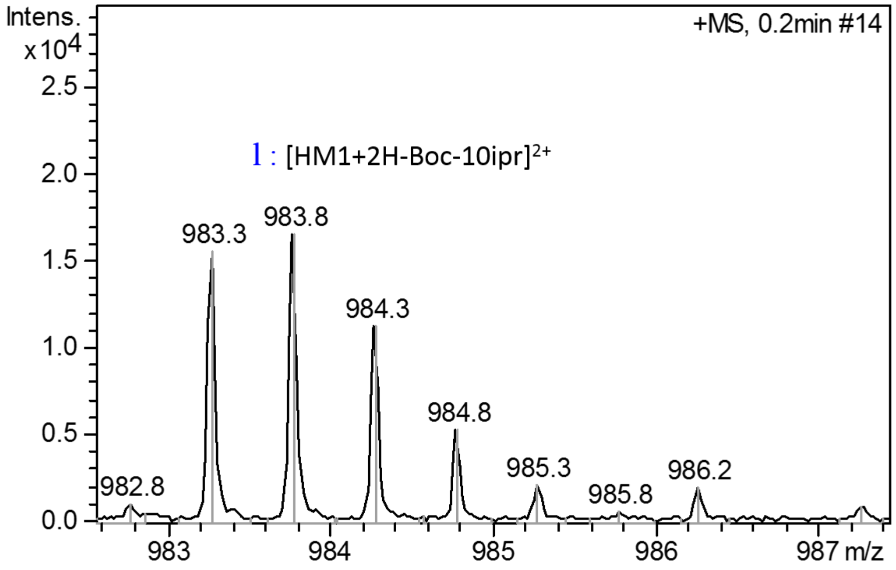
Intens.
x10^4
+MS, 0.2min #14
l : [HM1+2H-Boc-10ipr]2+
982.8
983.3
983.8
984.3
984.8
985.3
985.8
986.2
m/z

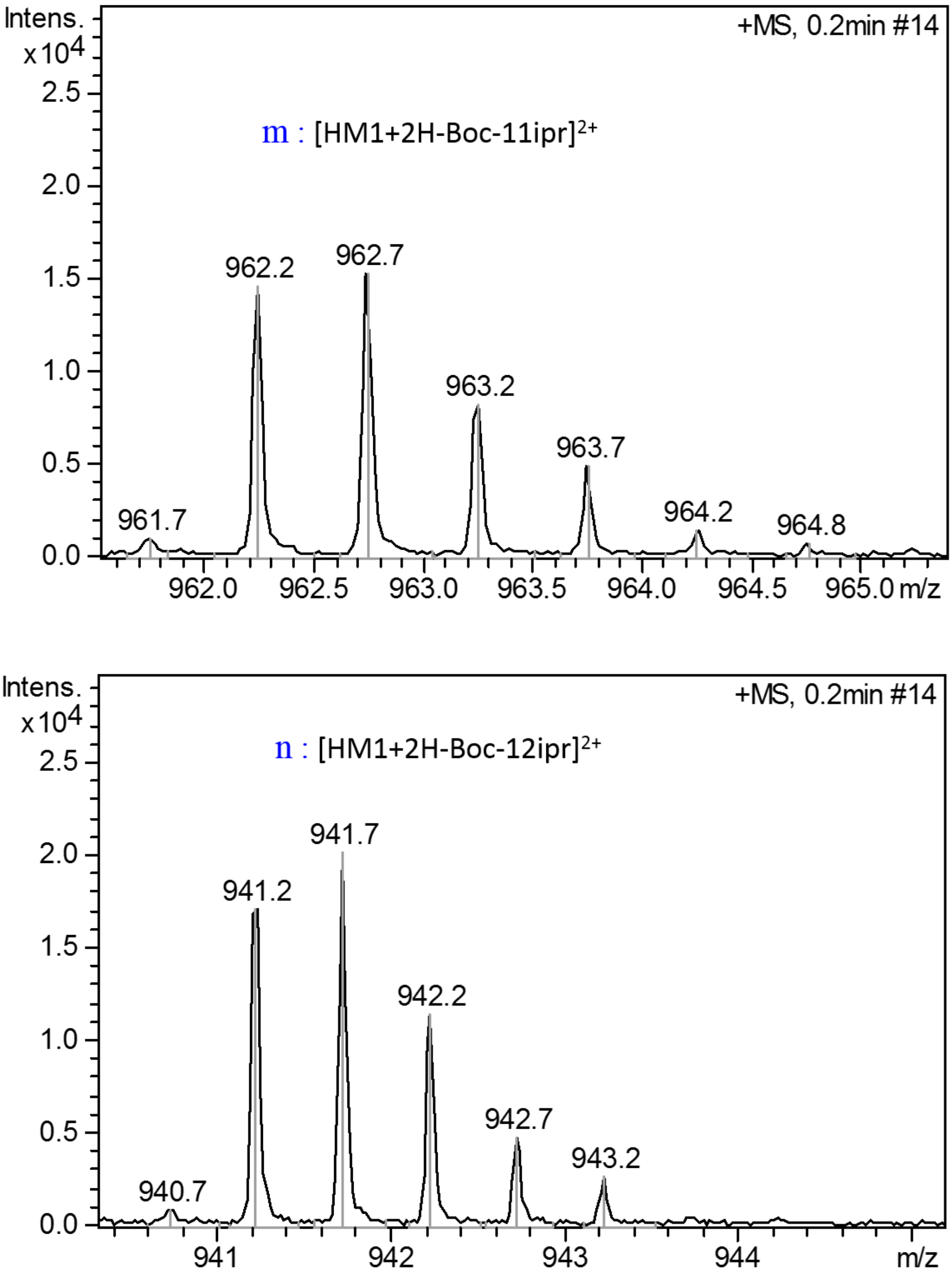

Figure S15. ESI MS spectrum of **HM1**.

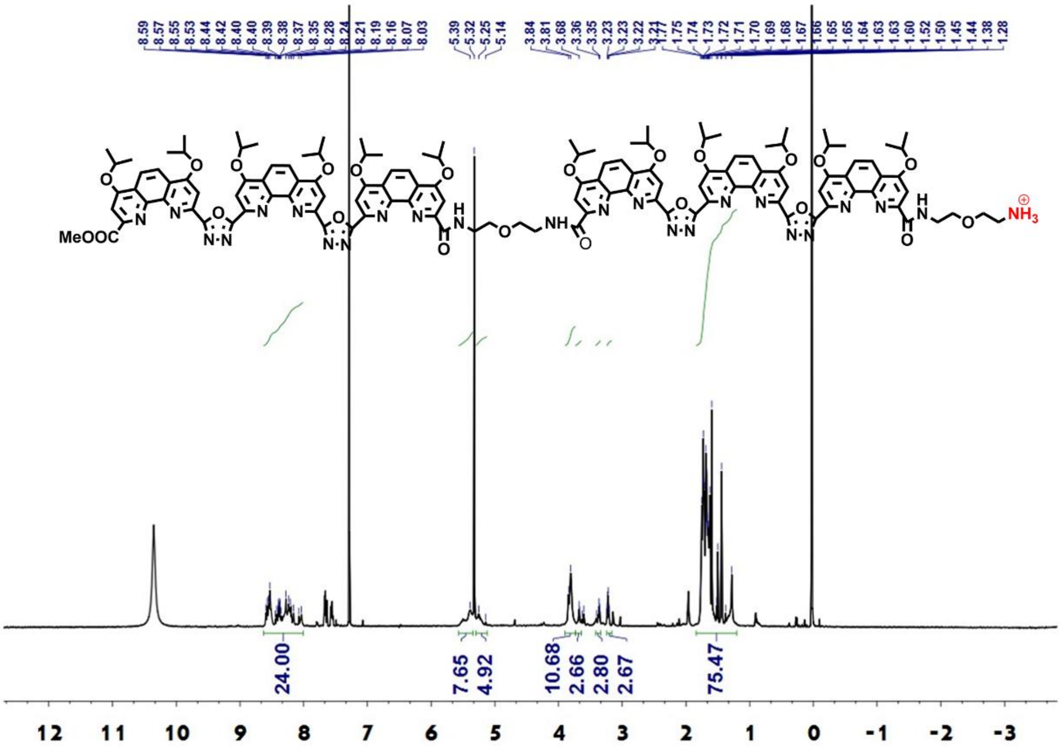

Figure S16. ^{1}H NMR spectrum of **HM2** in $CDCl_3$ ($CDCl_3$/ Trifluoroacetic acid-D 50:1).

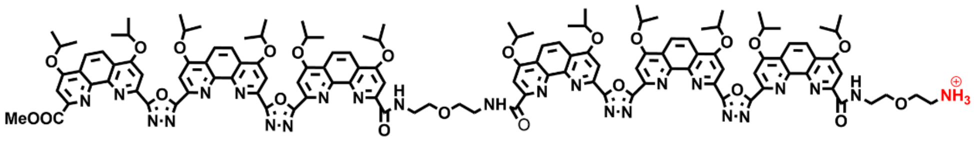

M$_2$=2385.9

ESI-full spectrum

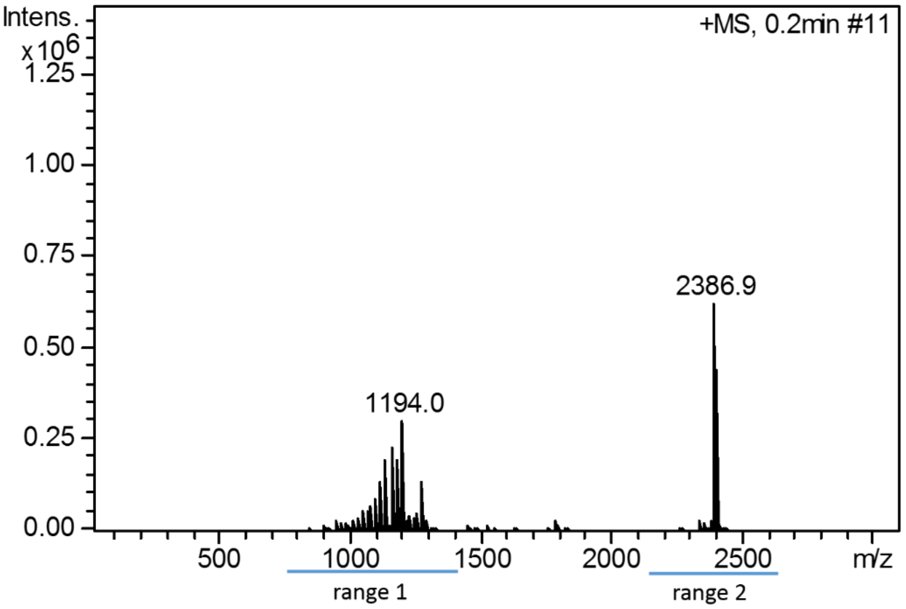

range 1

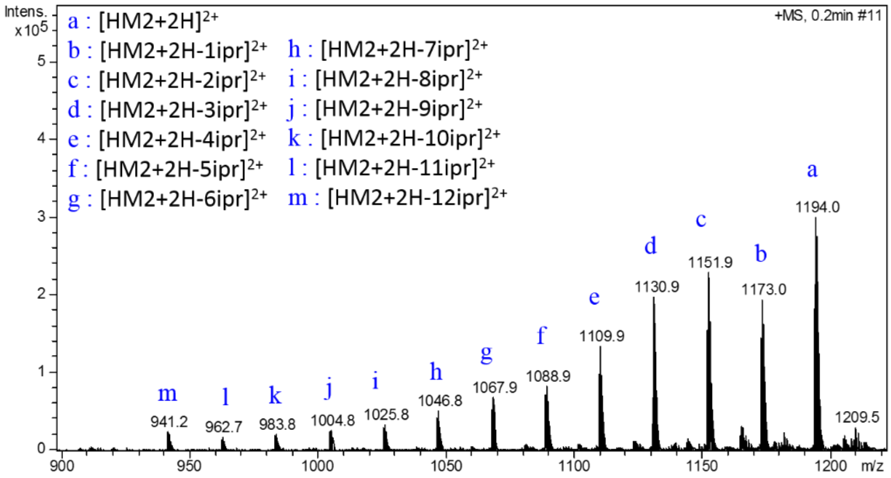

range 2

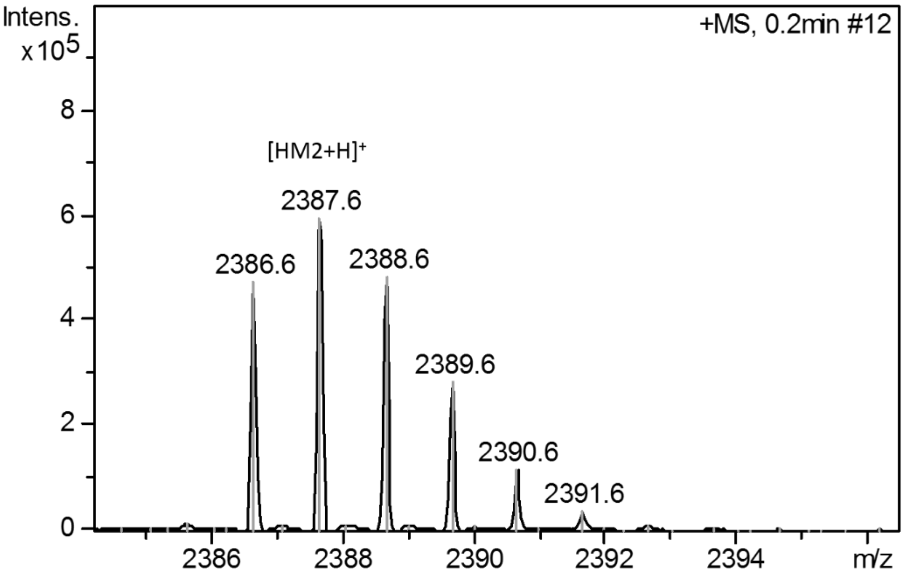

range 1

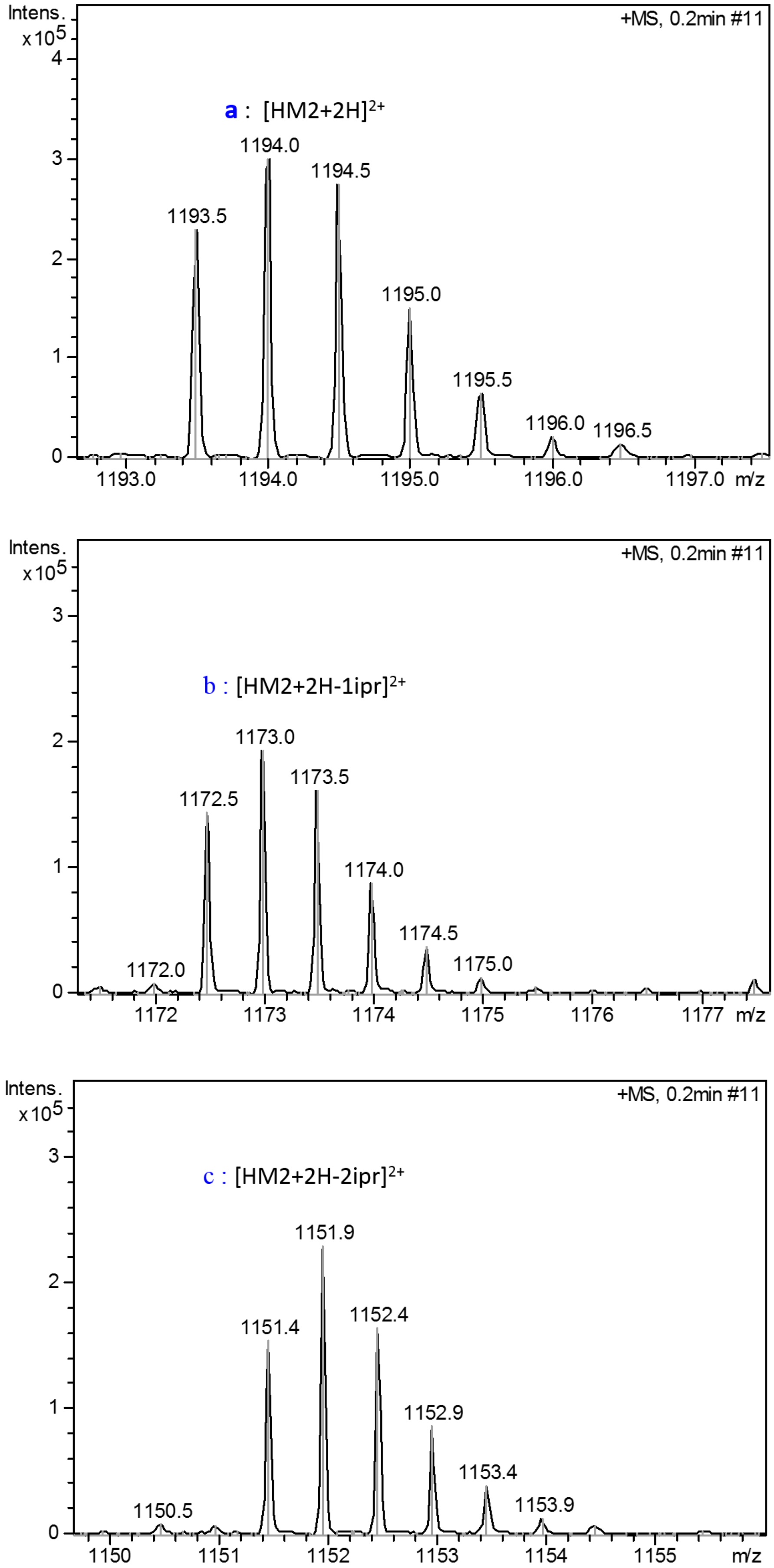
Intens. x10^5
+MS, 0.2min #11
a : [HM2+2H]2+
1193.5
1194.0
1194.5
1195.0
1195.5
1196.0
1196.5
1193.0 1194.0 1195.0 1196.0 1197.0 m/z
Intens. x10^5
+MS, 0.2min #11
b : [HM2+2H-1ipr]2+
1172.0
1172.5
1173.0
1173.5
1174.0
1174.5
1175.0
1172 1173 1174 1175 1176 1177 m/z
Intens. x10^5
+MS, 0.2min #11
c : [HM2+2H-2ipr]2+
1150.5
1151.4
1151.9
1152.4
1152.9
1153.4
1153.9
1150 1151 1152 1153 1154 1155 m/z

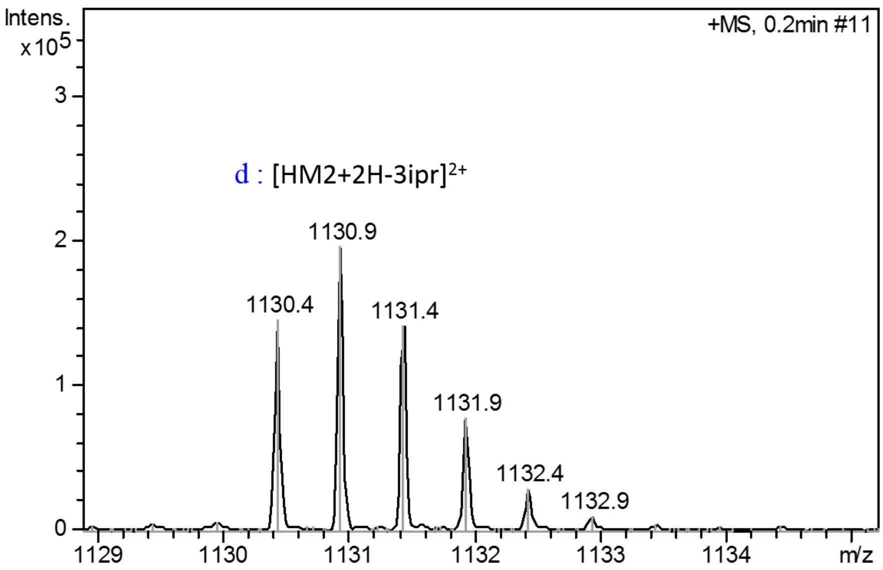
Intens.
x10^5
+MS, 0.2min #11
d : [HM2+2H-3ipr]2+
1130.4
1130.9
1131.4
1131.9
1132.4
1132.9
m/z

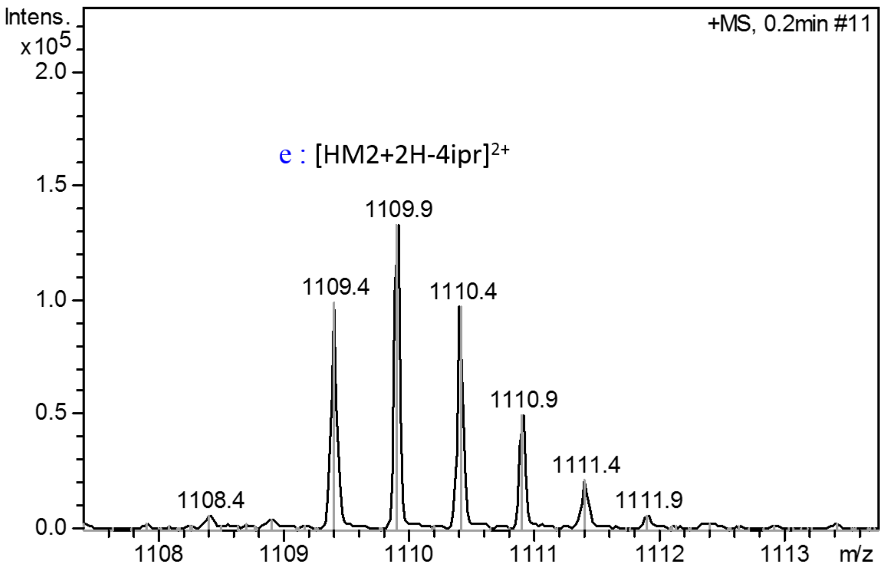
Intens.
x10^5
+MS, 0.2min #11
e : [HM2+2H-4ipr]2+
1108.4
1109.4
1109.9
1110.4
1110.9
1111.4
1111.9
m/z

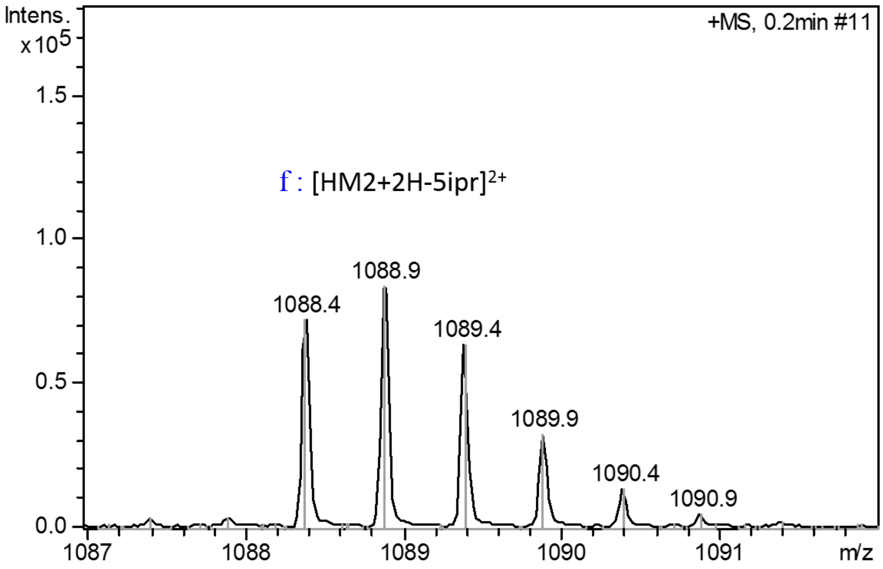
Intens.
x10^5
+MS, 0.2min #11
f : [HM2+2H-5ipr]2+
1088.4
1088.9
1089.4
1089.9
1090.4
1090.9
m/z

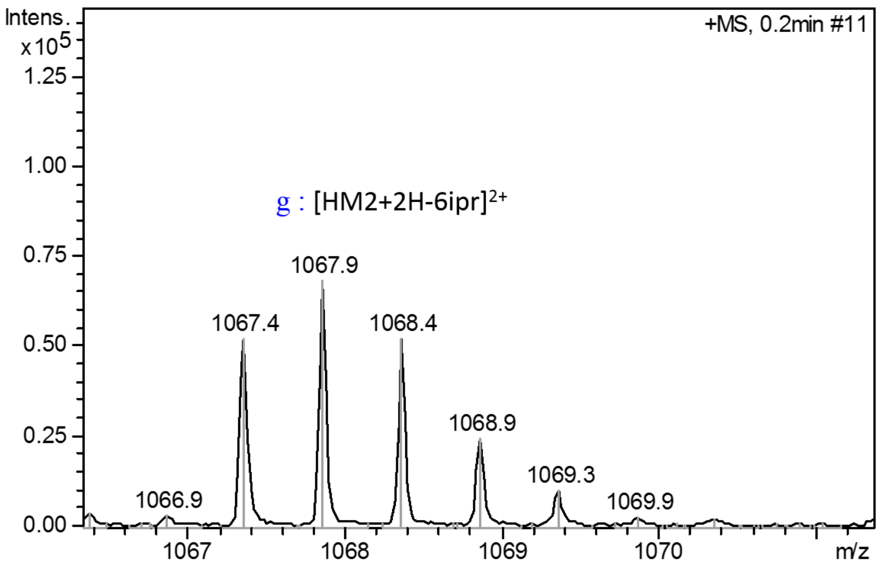
Intens.
x10^5
+MS, 0.2min #11
g : [HM2+2H-6ipr]2+
1067.9
1067.4
1068.4
1068.9
1069.3
1066.9
1069.9
1067
1068
1069
1070
m/z

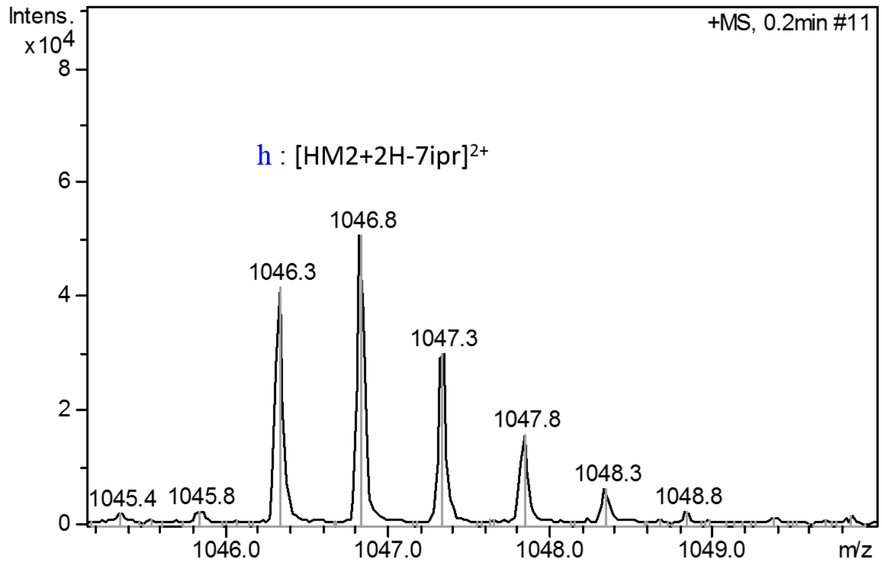
Intens.
x10^4
+MS, 0.2min #11
h : [HM2+2H-7ipr]2+
1046.8
1046.3
1047.3
1047.8
1048.3
1045.4
1045.8
1048.8
1046.0
1047.0
1048.0
1049.0
m/z

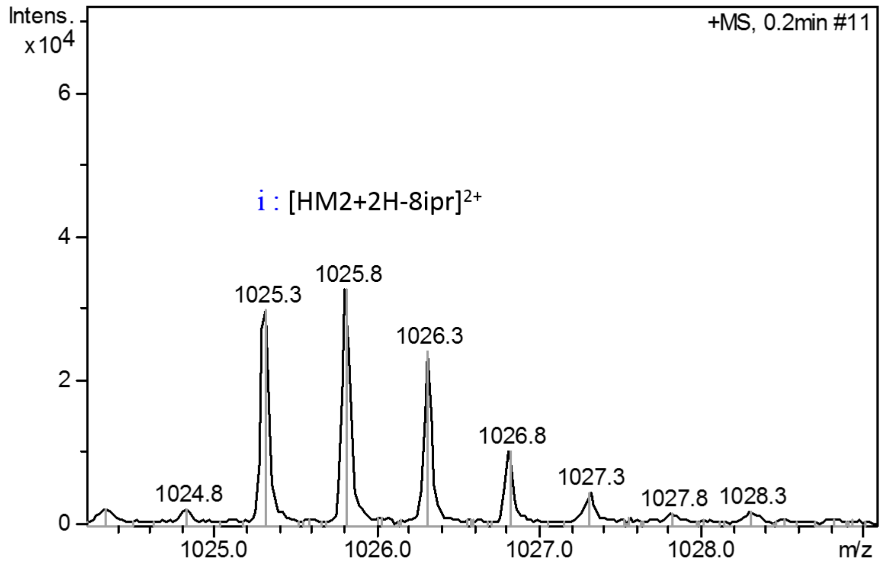
Intens.
x10^4
+MS, 0.2min #11
i : [HM2+2H-8ipr]2+
1025.8
1025.3
1026.3
1026.8
1027.3
1024.8
1027.8
1028.3
1025.0
1026.0
1027.0
1028.0
m/z

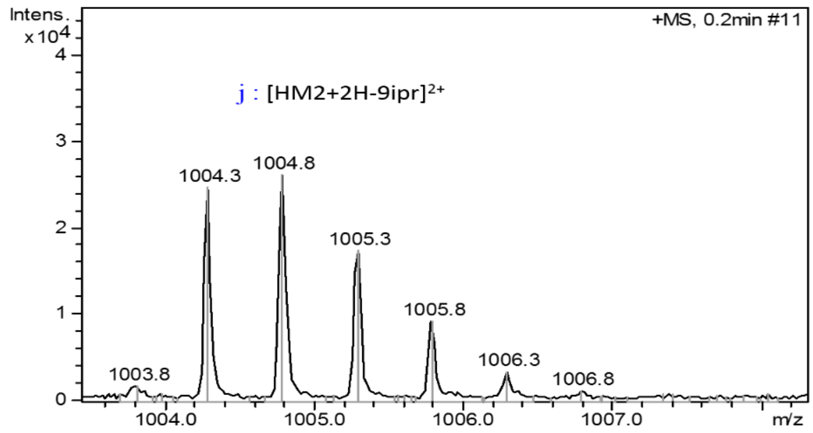
Intens.
x10^4
+MS, 0.2min #11
j : [HM2+2H-9ipr]2+
1003.8
1004.3
1004.8
1005.3
1005.8
1006.3
1006.8
1004.0
1005.0
1006.0
1007.0
m/z

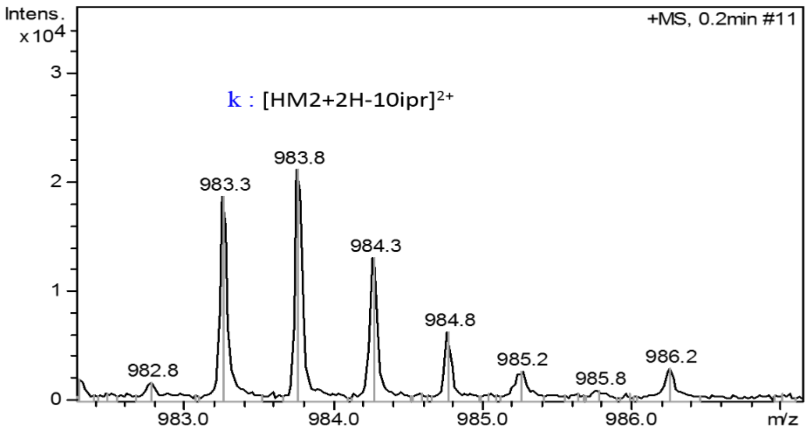
Intens.
x10^4
+MS, 0.2min #11
k : [HM2+2H-10ipr]2+
982.8
983.3
983.8
984.3
984.8
985.2
985.8
986.2
983.0
984.0
985.0
986.0
m/z

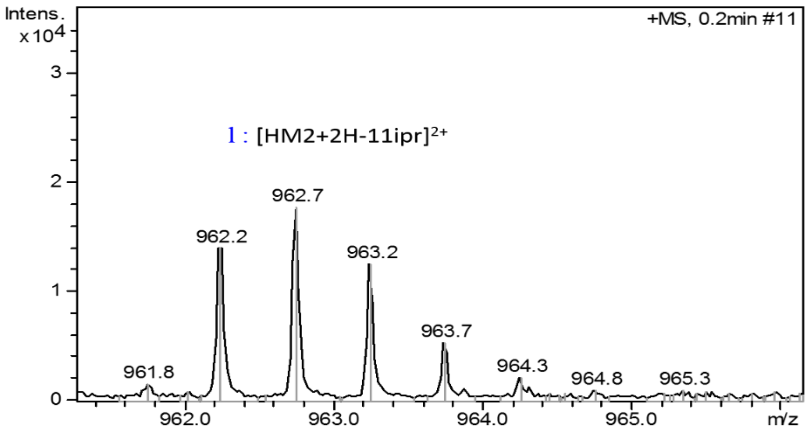
Intens.
x10^4
+MS, 0.2min #11
l : [HM2+2H-11ipr]2+
961.8
962.2
962.7
963.2
963.7
964.3
964.8
965.3
962.0
963.0
964.0
965.0
m/z

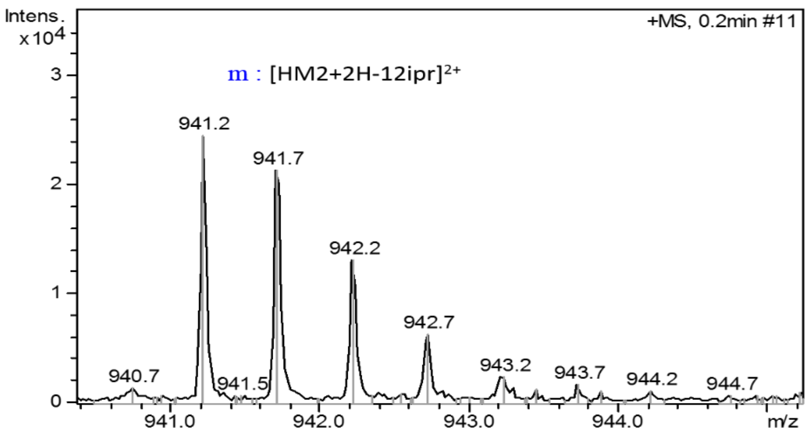
Intens.
x10^4
+MS, 0.2min #11
m : [HM2+2H-12ipr]2+
940.7
941.2
941.5
941.7
942.2
942.7
943.2
943.7
944.2
944.7
941.0
942.0
943.0
944.0
m/z

Figure S17. ESI MS spectrum of **HM2**.

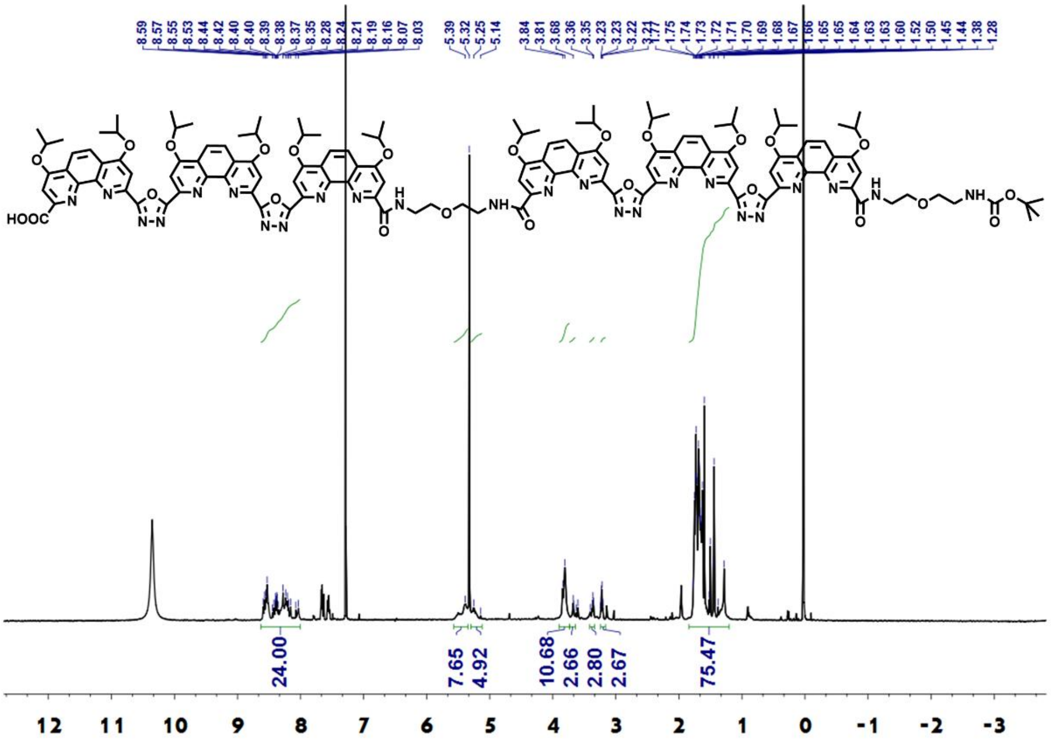

Figure S18. ^{1}H NMR spectrum of **HM3** in $CDCl_3$ ($CDCl_3$/ Trifluoroacetic acid-D 50:1).

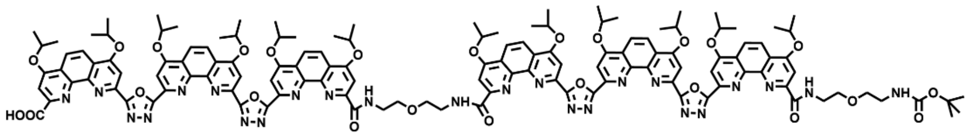

M_3=2471

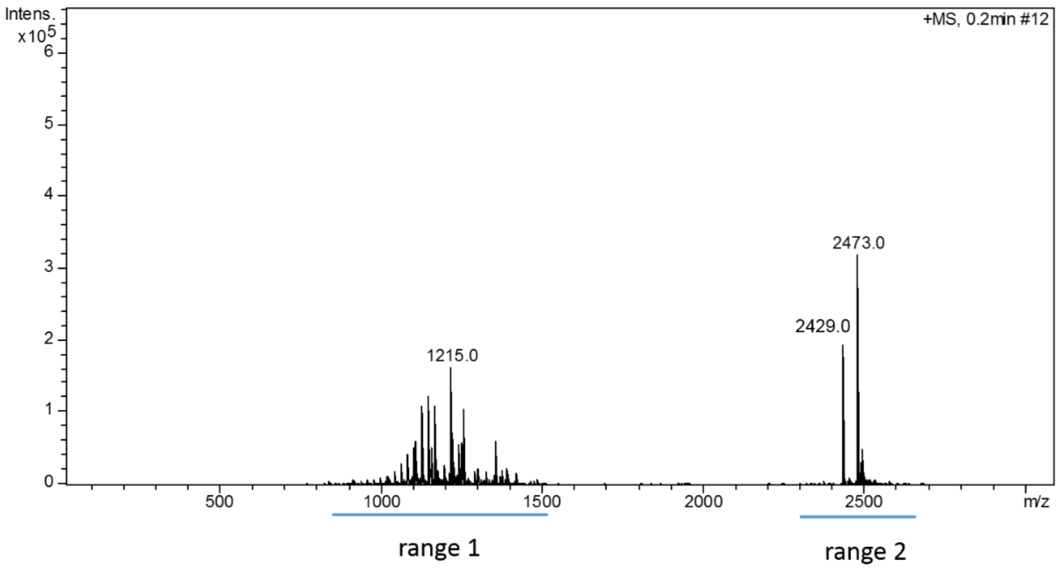
Intens.
x10^5
+MS, 0.2min #12
2473.0
2429.0
1215.0
m/z
range 1
range 2

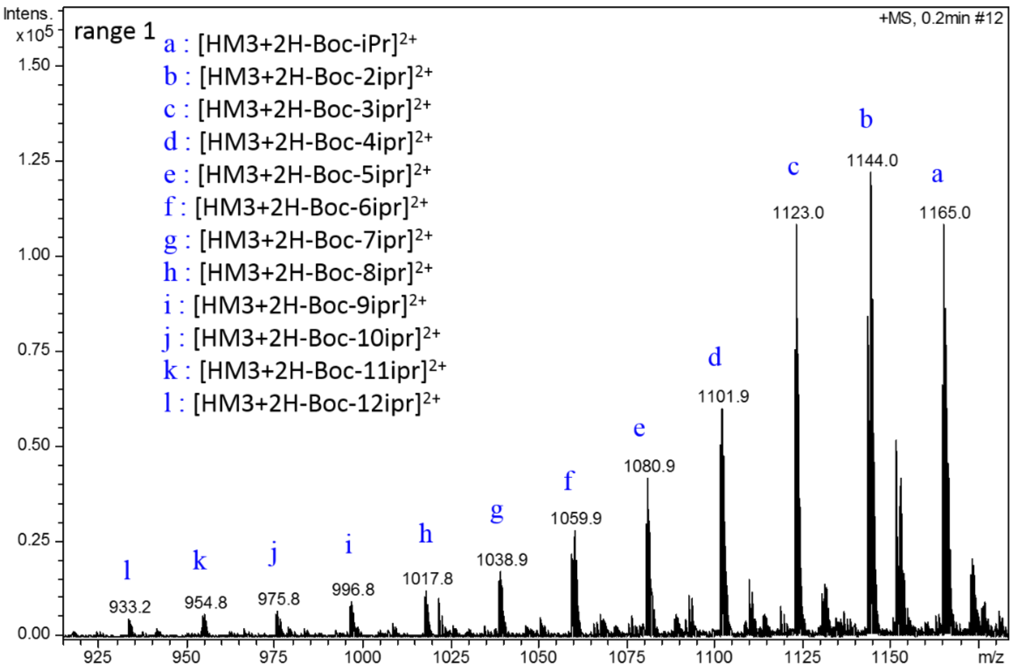
Intens.
x10^5
range 1
+MS, 0.2min #12
a : [HM3+2H-Boc-iPr]2+
b : [HM3+2H-Boc-2ipr]2+
c : [HM3+2H-Boc-3ipr]2+
d : [HM3+2H-Boc-4ipr]2+
e : [HM3+2H-Boc-5ipr]2+
f : [HM3+2H-Boc-6ipr]2+
g : [HM3+2H-Boc-7ipr]2+
h : [HM3+2H-Boc-8ipr]2+
i : [HM3+2H-Boc-9ipr]2+
j : [HM3+2H-Boc-10ipr]2+
k : [HM3+2H-Boc-11ipr]2+
l : [HM3+2H-Boc-12ipr]2+
b 1144.0
c 1123.0
a 1165.0
d 1101.9
e 1080.9
f 1059.9
g 1038.9
h 1017.8
i 996.8
j 975.8
k 954.8
l 933.2
m/z

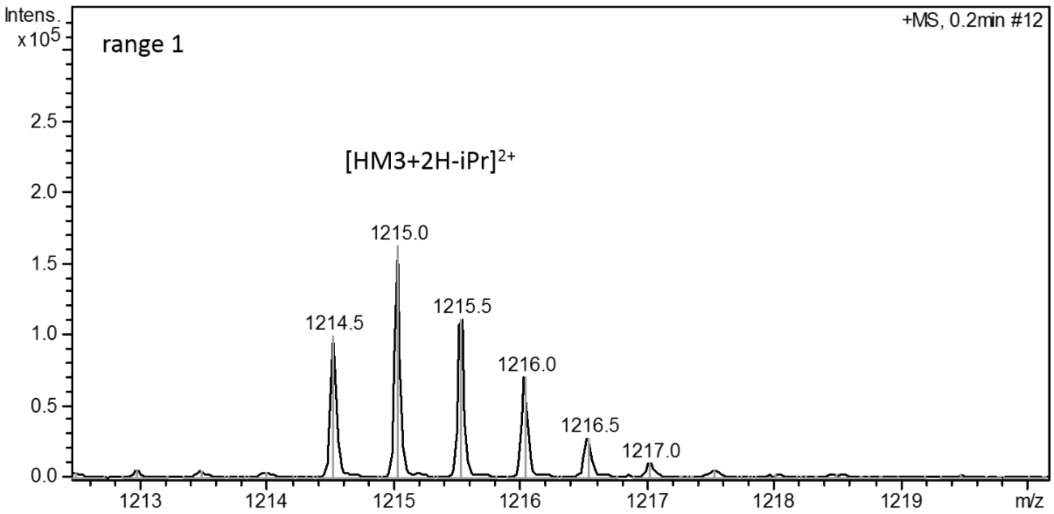
Intens.
x10^5
range 1
+MS, 0.2min #12
[HM3+2H-iPr]2+
1215.0
1214.5
1215.5
1216.0
1216.5
1217.0
m/z

Range 2

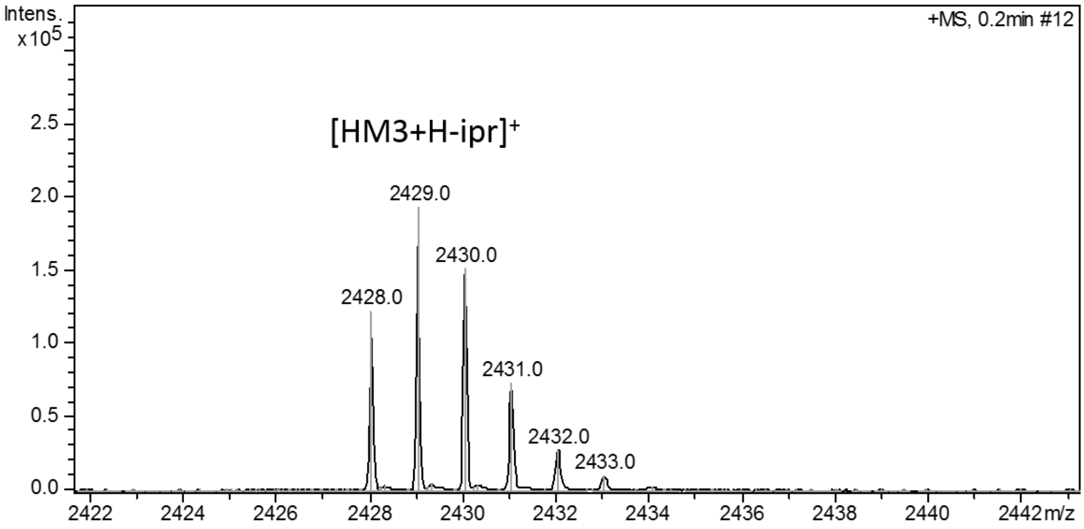

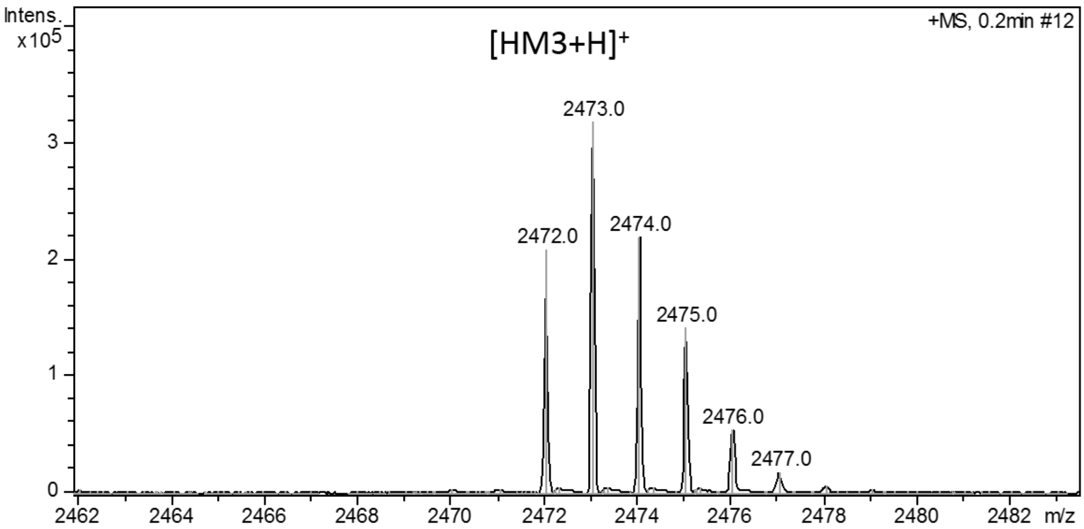

Figure S19. ESI MS spectrum of **HM3**.

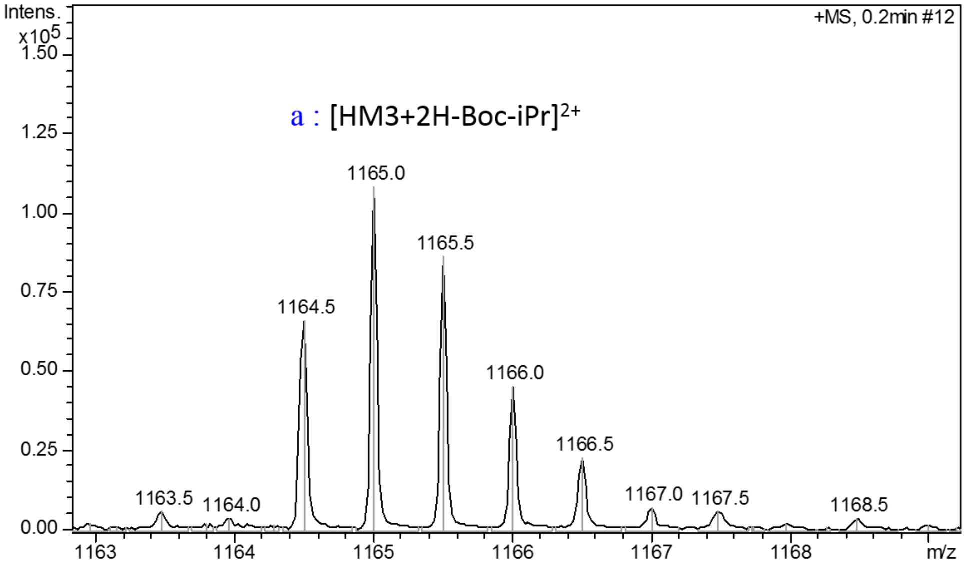
Intens.
x10^5
+MS, 0.2min #12
a : [HM3+2H-Boc-iPr]2+
1163.5
1164.0
1164.5
1165.0
1165.5
1166.0
1166.5
1167.0
1167.5
1168.5
m/z

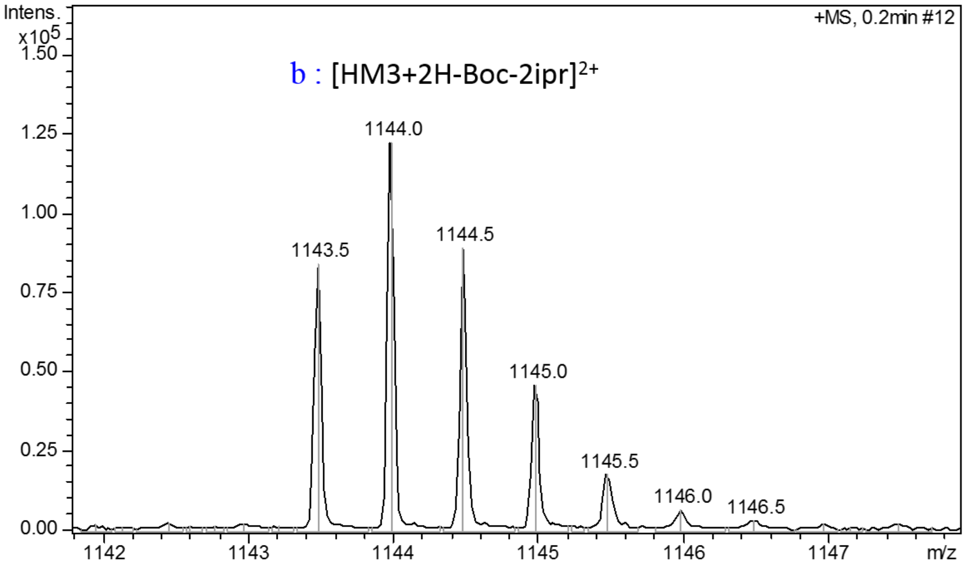
Intens.
x10^5
+MS, 0.2min #12
b : [HM3+2H-Boc-2ipr]2+
1143.5
1144.0
1144.5
1145.0
1145.5
1146.0
1146.5
m/z

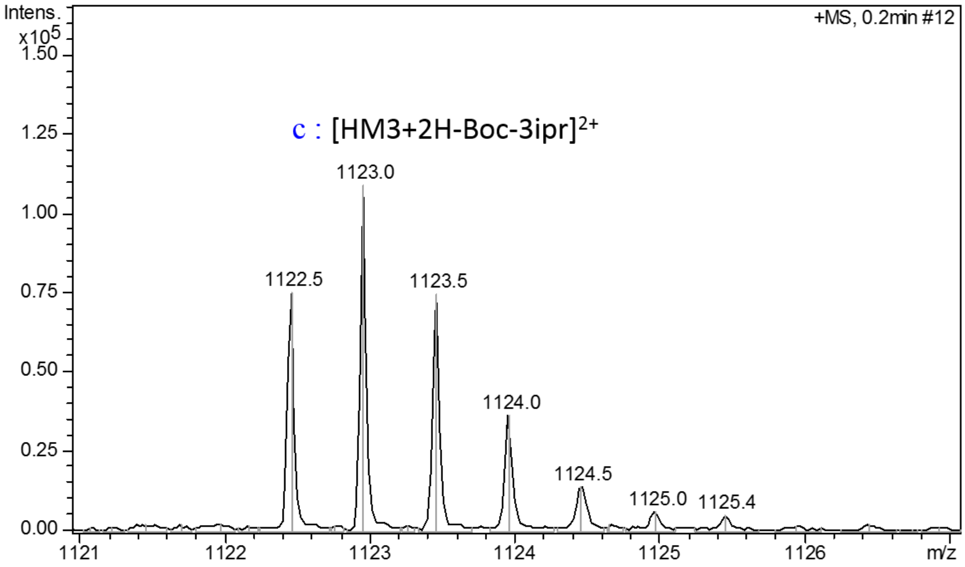
Intens.
x10^5
+MS, 0.2min #12
c : [HM3+2H-Boc-3ipr]2+
1122.5
1123.0
1123.5
1124.0
1124.5
1125.0
1125.4
m/z

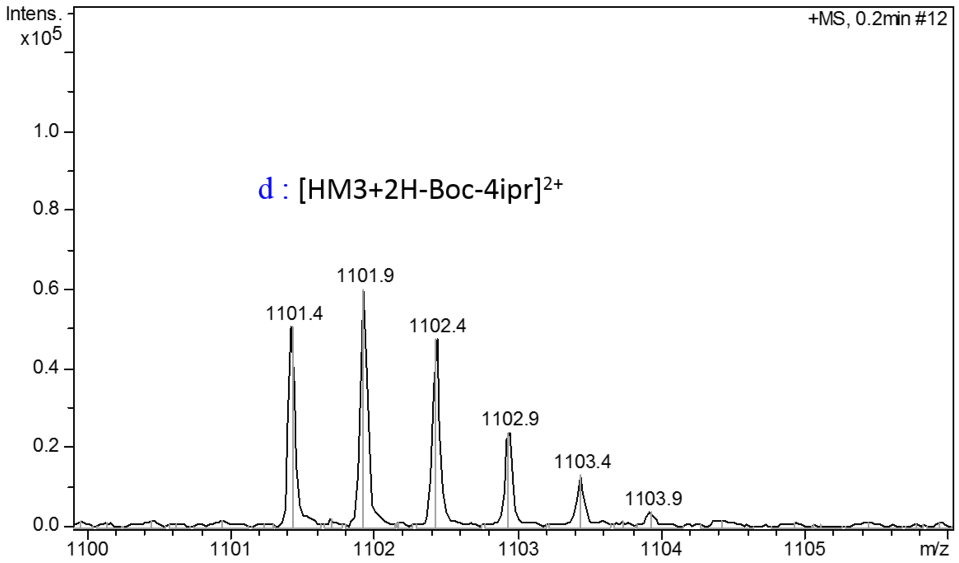
Intens.
x10^5
+MS, 0.2min #12
d : [HM3+2H-Boc-4ipr]2+
1.0
0.8
0.6
0.4
0.2
0.0
1101.4
1101.9
1102.4
1102.9
1103.4
1103.9
1100
1101
1102
1103
1104
1105
m/z

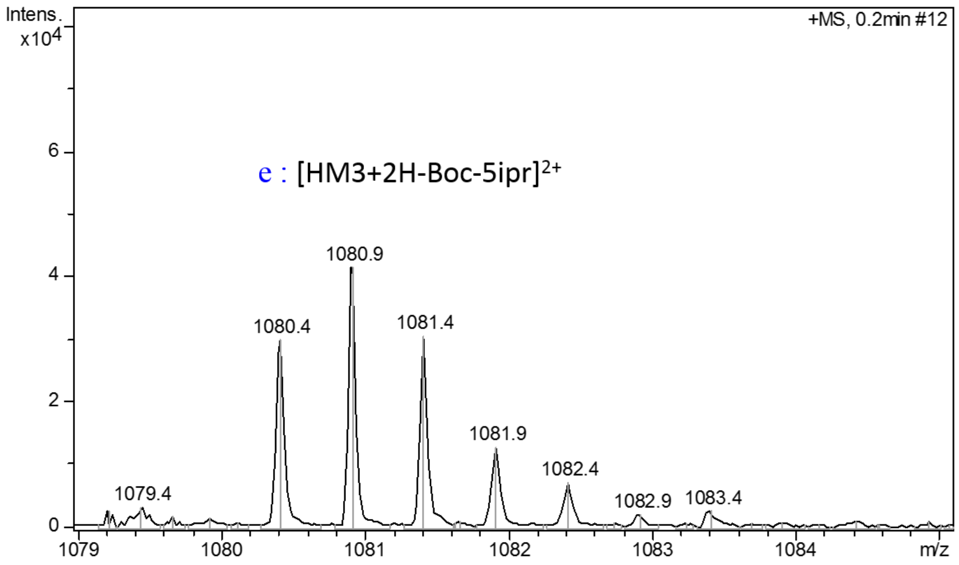
Intens.
x10^4
+MS, 0.2min #12
e : [HM3+2H-Boc-5ipr]2+
6
4
2
0
1079.4
1080.4
1080.9
1081.4
1081.9
1082.4
1082.9
1083.4
1079
1080
1081
1082
1083
1084
m/z

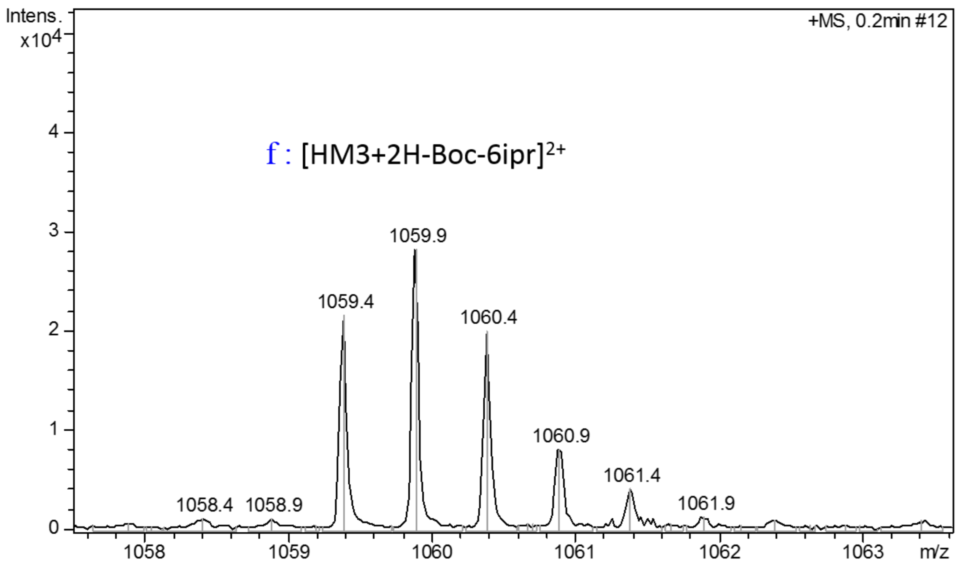
Intens.
x10^4
+MS, 0.2min #12
f : [HM3+2H-Boc-6ipr]2+
4
3
2
1
0
1058.4
1058.9
1059.4
1059.9
1060.4
1060.9
1061.4
1061.9
1058
1059
1060
1061
1062
1063
m/z

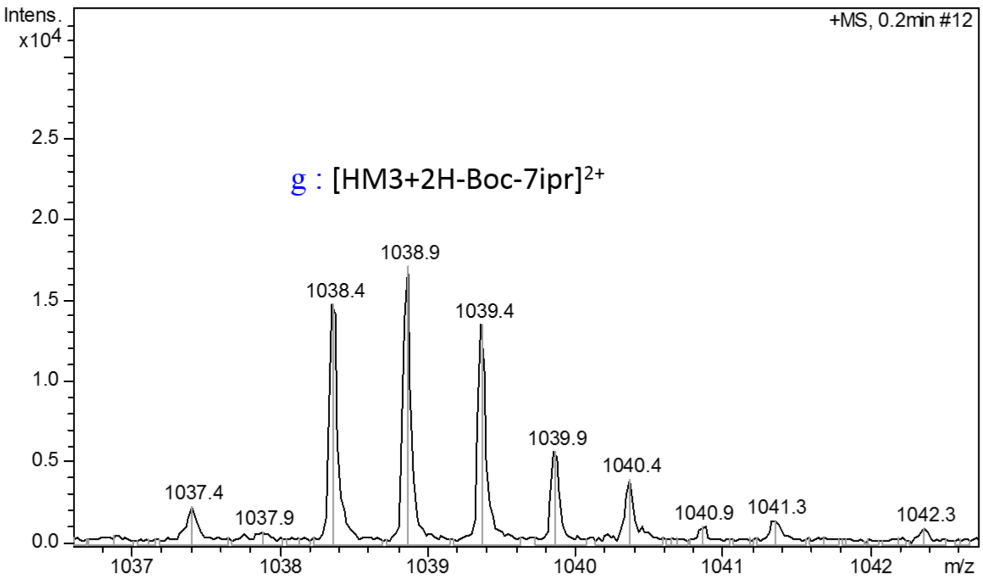
Intens.
x10^4
+MS, 0.2min #12
g : [HM3+2H-Boc-7ipr]2+
2.5
2.0
1.5
1.0
0.5
0.0
1037.4
1037.9
1038.4
1038.9
1039.4
1039.9
1040.4
1040.9
1041.3
1042.3
1037
1038
1039
1040
1041
1042
m/z

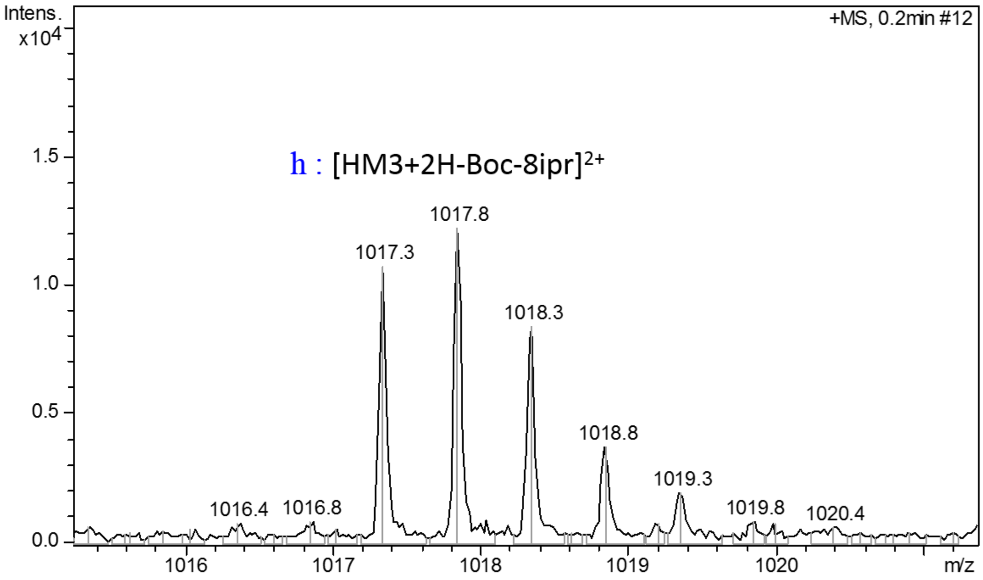
Intens.
x10^4
+MS, 0.2min #12
h : [HM3+2H-Boc-8ipr]2+
1.5
1.0
0.5
0.0
1016.4
1016.8
1017.3
1017.8
1018.3
1018.8
1019.3
1019.8
1020.4
1016
1017
1018
1019
1020
m/z

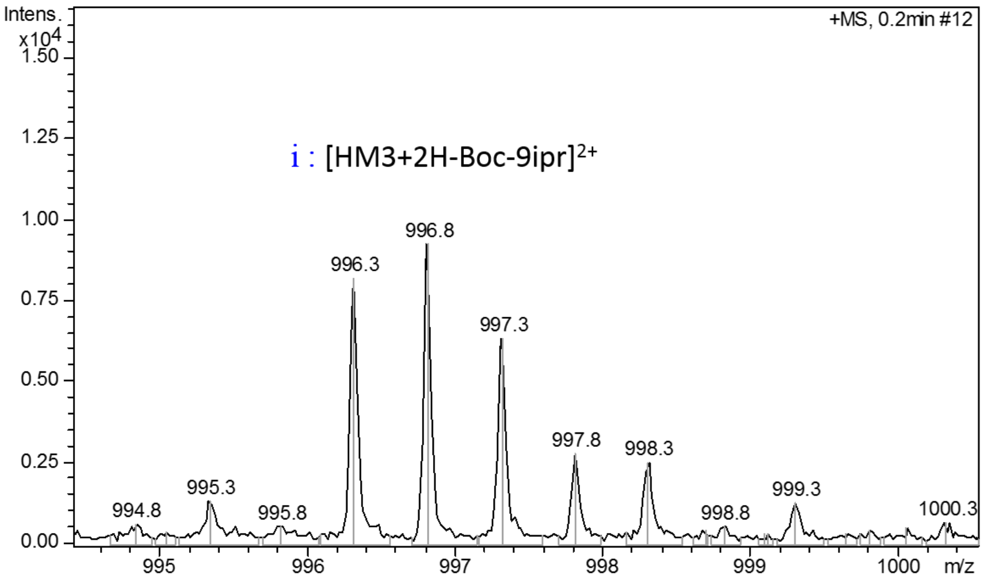
Intens.
x10^4
+MS, 0.2min #12
i : [HM3+2H-Boc-9ipr]2+
1.50
1.25
1.00
0.75
0.50
0.25
0.00
994.8
995.3
995.8
996.3
996.8
997.3
997.8
998.3
998.8
999.3
1000.3
995
996
997
998
999
1000
m/z

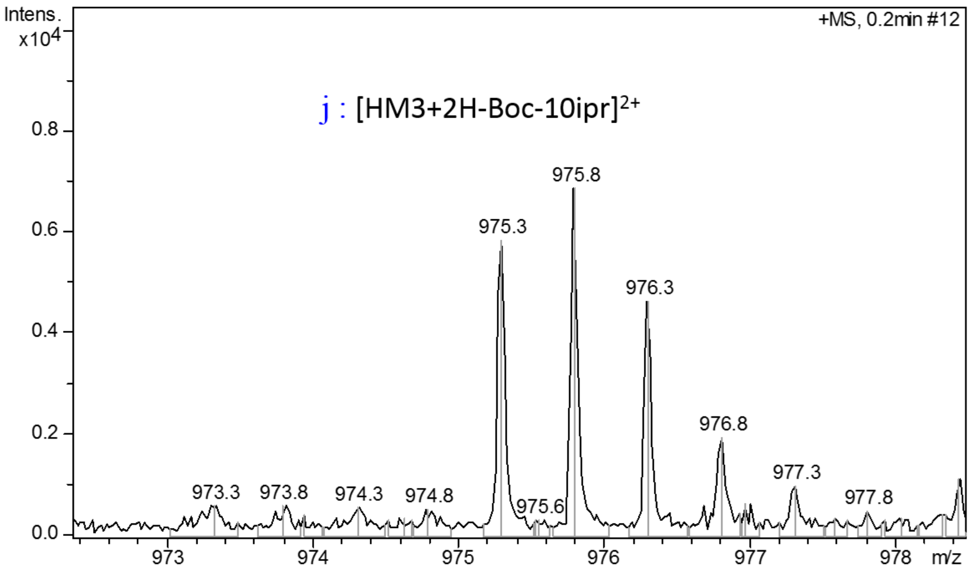
Intens.
x10^4
+MS, 0.2min #12
j : [HM3+2H-Boc-10ipr]2+
0.8
0.6
0.4
0.2
0.0
975.8
975.3
976.3
976.8
973.3
973.8
974.3
974.8
975.6
977.3
977.8
973
974
975
976
977
978
m/z

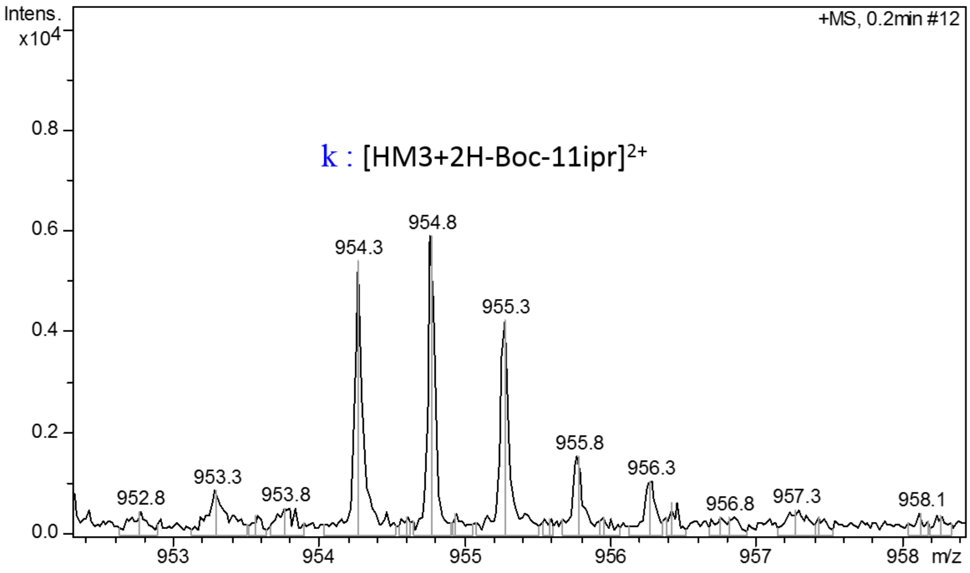
Intens.
x10^4
+MS, 0.2min #12
k : [HM3+2H-Boc-11ipr]2+
0.8
0.6
0.4
0.2
0.0
954.8
954.3
955.3
955.8
956.3
952.8
953.3
953.8
956.8
957.3
958.1
953
954
955
956
957
958
m/z

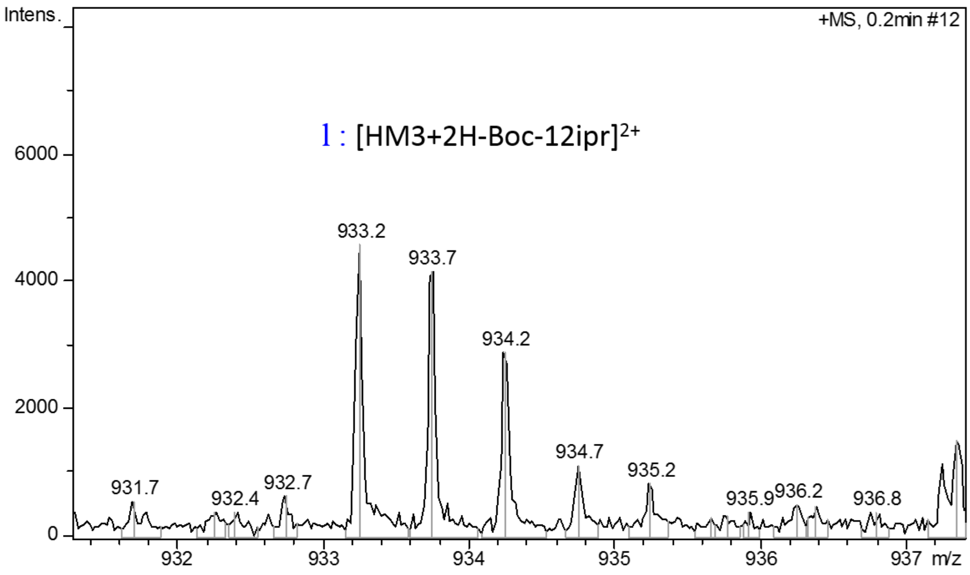
Intens.
+MS, 0.2min #12
l : [HM3+2H-Boc-12ipr]2+
6000
4000
2000
0
933.2
933.7
934.2
934.7
935.2
931.7
932.4
932.7
935.9
936.2
936.8
932
933
934
935
936
937
m/z

Figure S20. ESI MS spectrum of **HM3**.

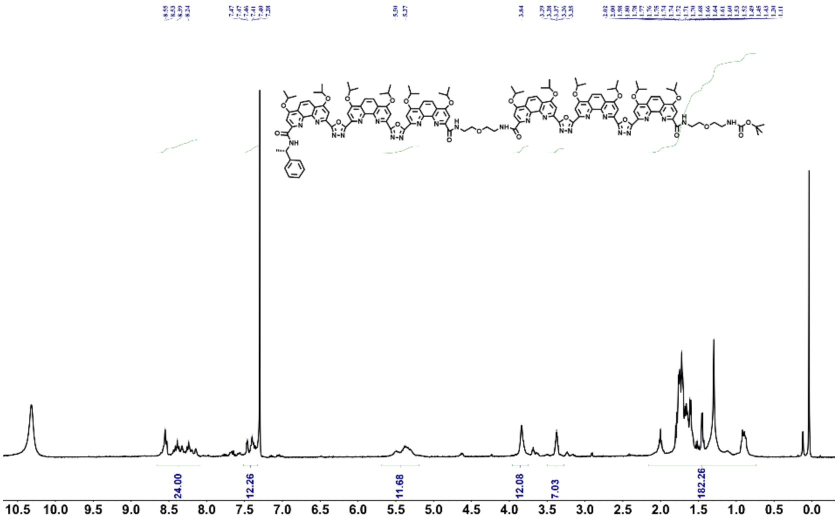

Figure S21. ¹H NMR spectrum of **HM4** in $CDCl_3$ ($CDCl_3$/ Trifluoroacetic acid-D 50:1).

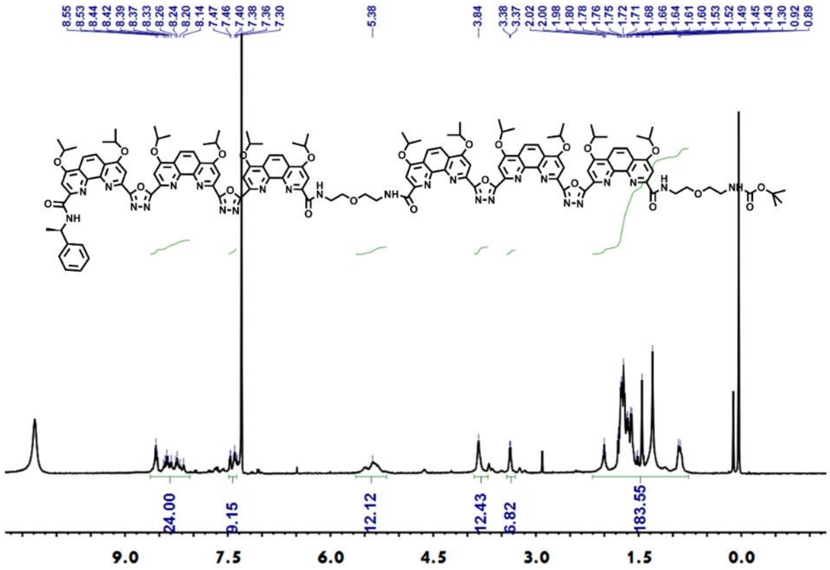

Figure S22. ¹H NMR spectrum of **HM5** in $CDCl_3$ ($CDCl_3$/ Trifluoroacetic acid-D 50:1).

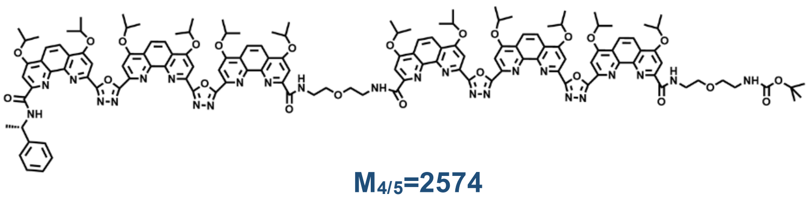

$M_{4/5}$=2574

ESI-full spectrum

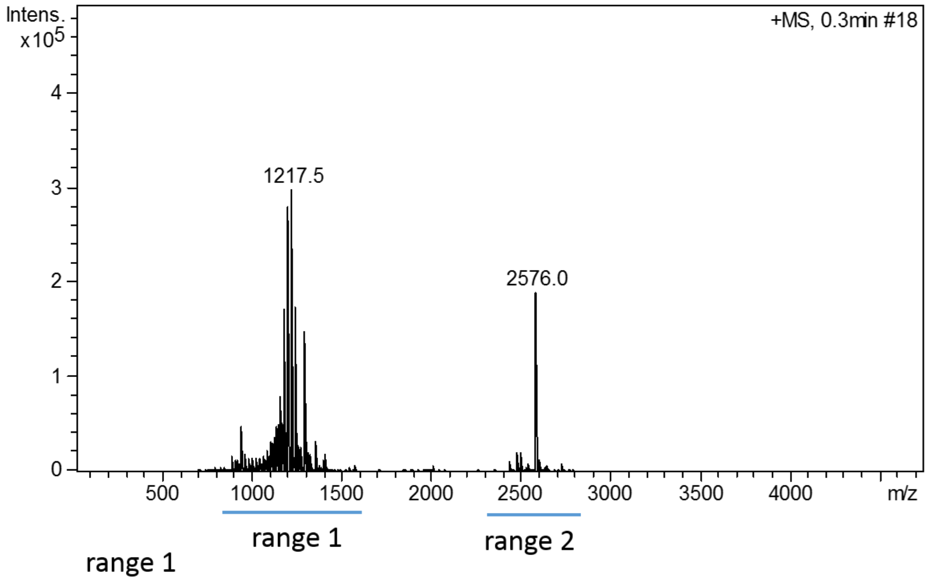

range 1

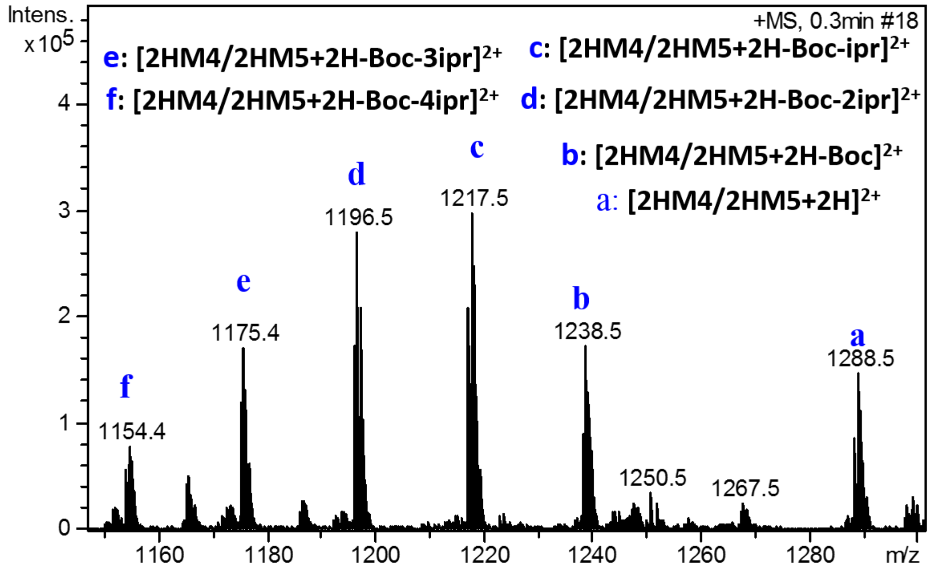

range 2

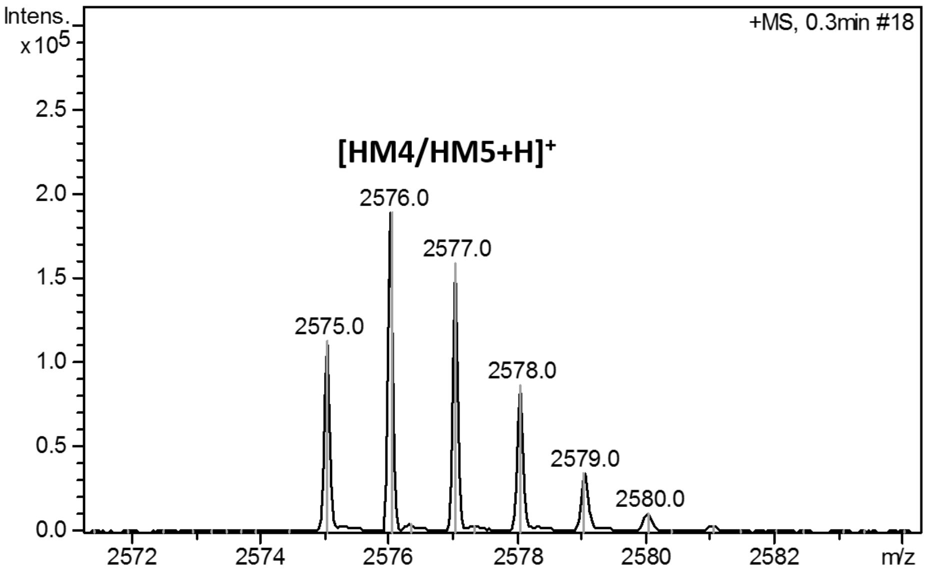

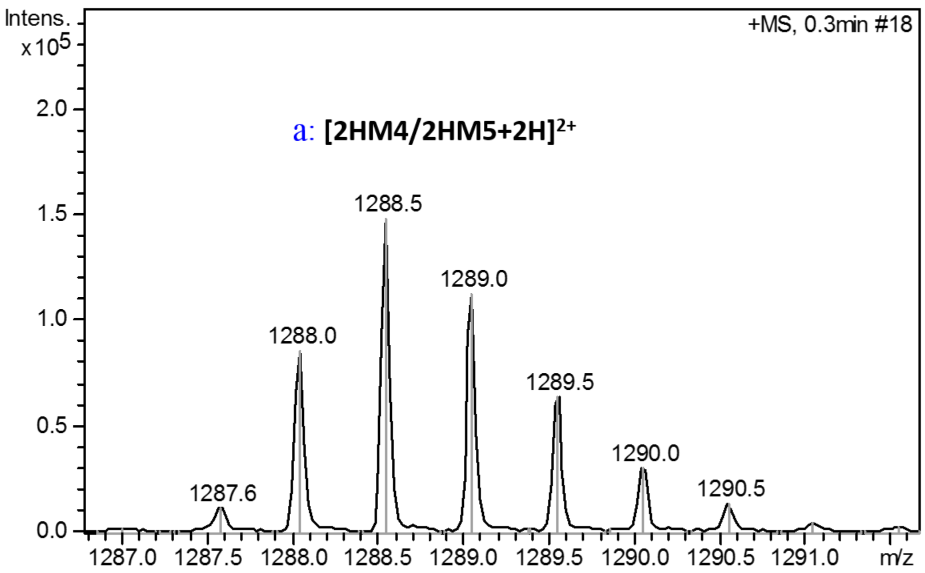
Intens.
x10^5
+MS, 0.3min #18
a: [2HM4/2HM5+2H]2+
2.0
1.5
1.0
0.5
0.0
1287.6
1288.0
1288.5
1289.0
1289.5
1290.0
1290.5
1287.0 1287.5 1288.0 1288.5 1289.0 1289.5 1290.0 1290.5 1291.0 m/z

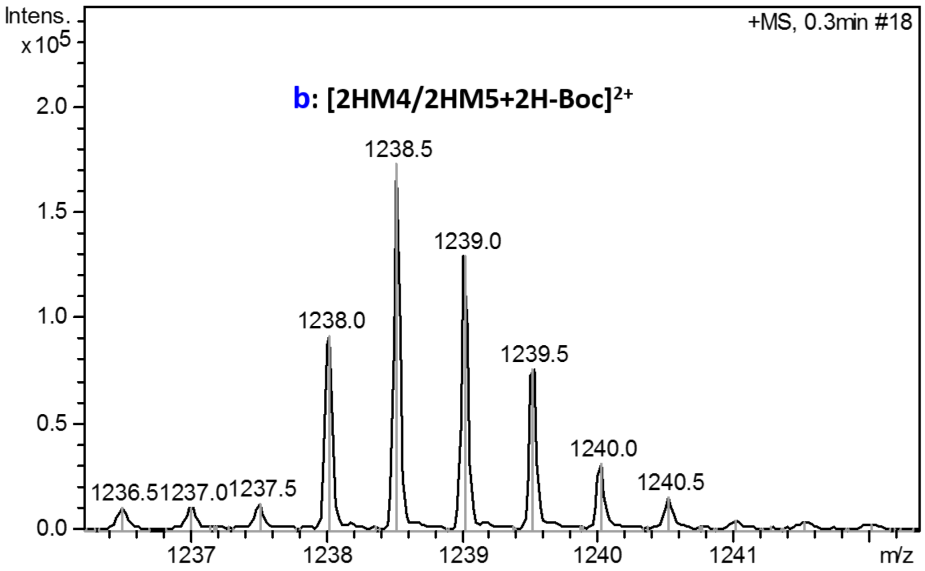
Intens.
x10^5
+MS, 0.3min #18
b: [2HM4/2HM5+2H-Boc]2+
2.0
1.5
1.0
0.5
0.0
1236.5
1237.0
1237.5
1238.0
1238.5
1239.0
1239.5
1240.0
1240.5
1237 1238 1239 1240 1241 m/z

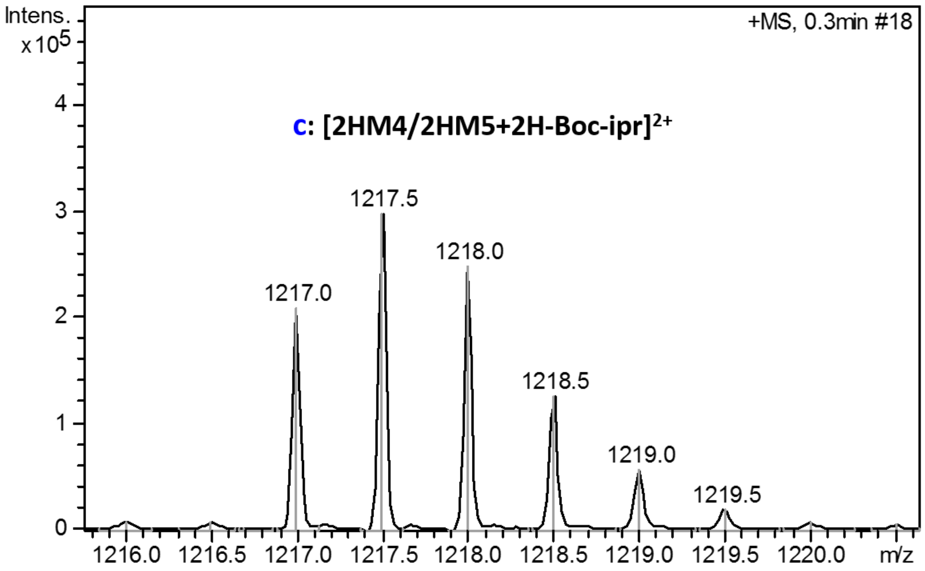
Intens.
x10^5
+MS, 0.3min #18
c: [2HM4/2HM5+2H-Boc-ipr]2+
4
3
2
1
0
1217.0
1217.5
1218.0
1218.5
1219.0
1219.5
1216.0 1216.5 1217.0 1217.5 1218.0 1218.5 1219.0 1219.5 1220.0 m/z

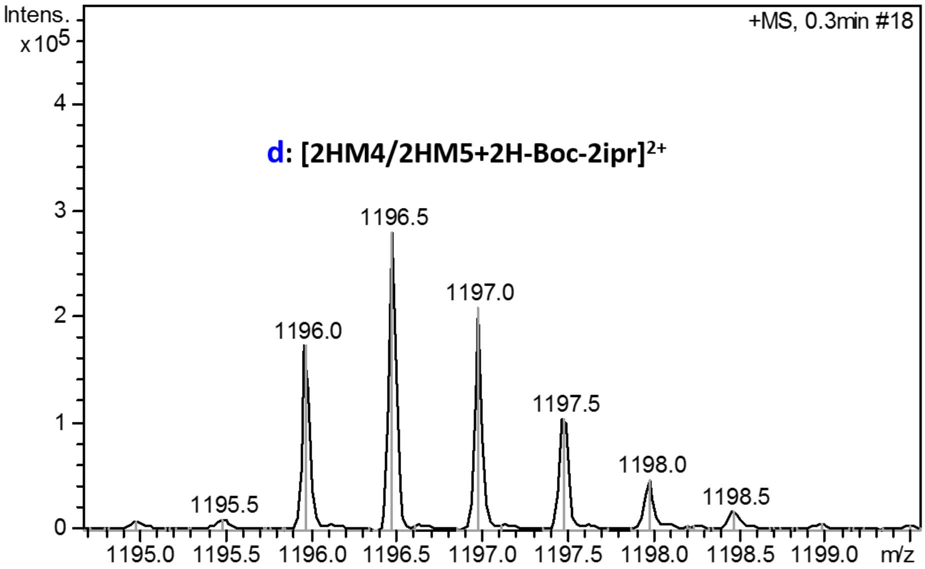
Intens. x10^5
+MS, 0.3min #18
d: [2HM4/2HM5+2H-Boc-2ipr]2+
1196.5
1197.0
1196.0
1197.5
1198.0
1198.5
1195.5
m/z

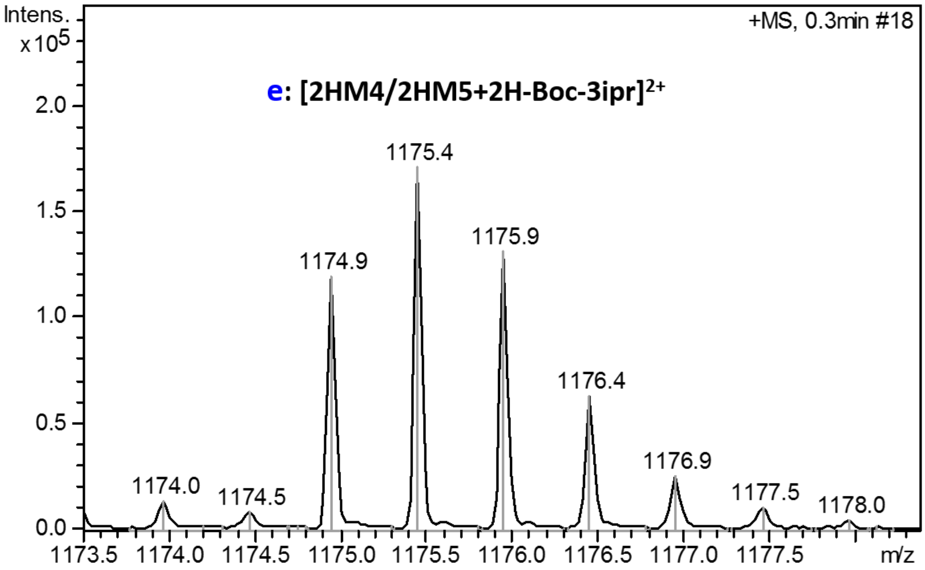
Intens. x10^5
+MS, 0.3min #18
e: [2HM4/2HM5+2H-Boc-3ipr]2+
1175.4
1175.9
1174.9
1176.4
1176.9
1174.0
1174.5
1177.5
1178.0
m/z

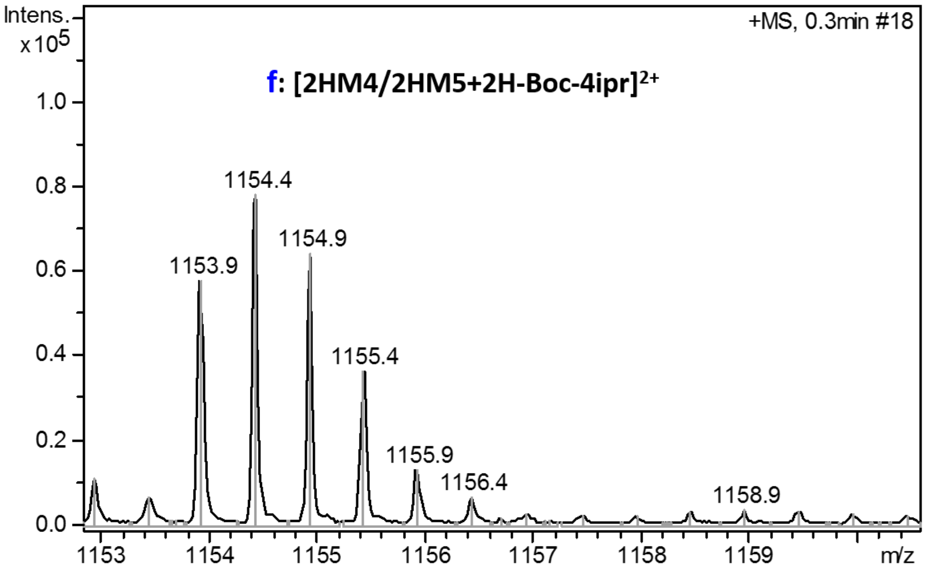
Intens. x10^5
+MS, 0.3min #18
f: [2HM4/2HM5+2H-Boc-4ipr]2+
1154.4
1154.9
1153.9
1155.4
1155.9
1156.4
1158.9
m/z

Figure S23. ESI MS spectrum of **HM4** and **HM5**.

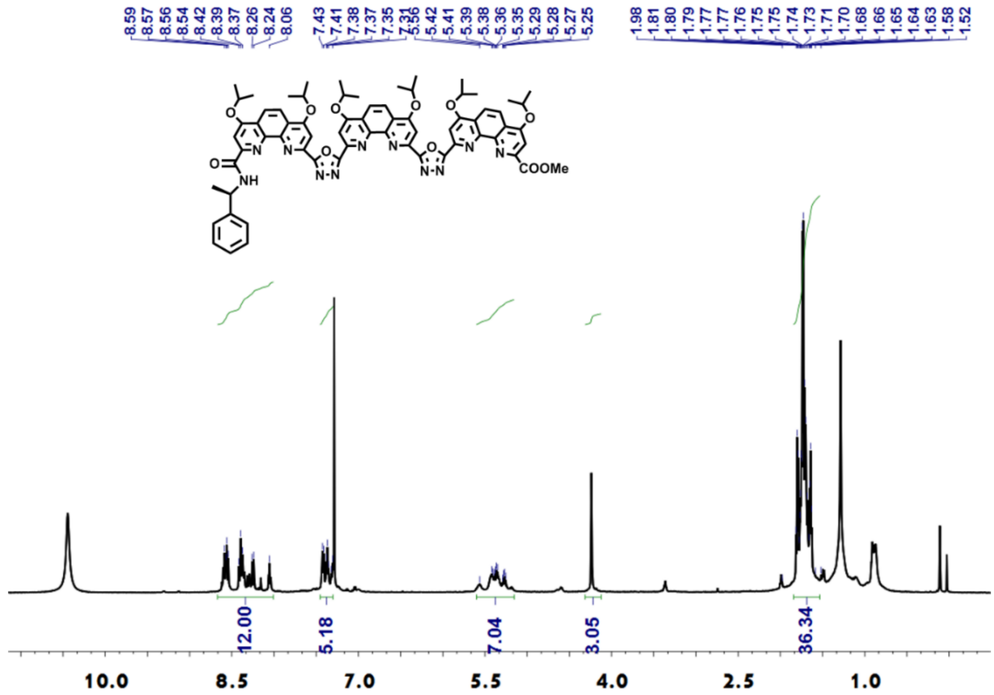

Figure S24. ^{1}H NMR spectrum of **HM7** in $CDCl_3$ ($CDCl_3$/Trifluoroacetic acid-D 50:1).

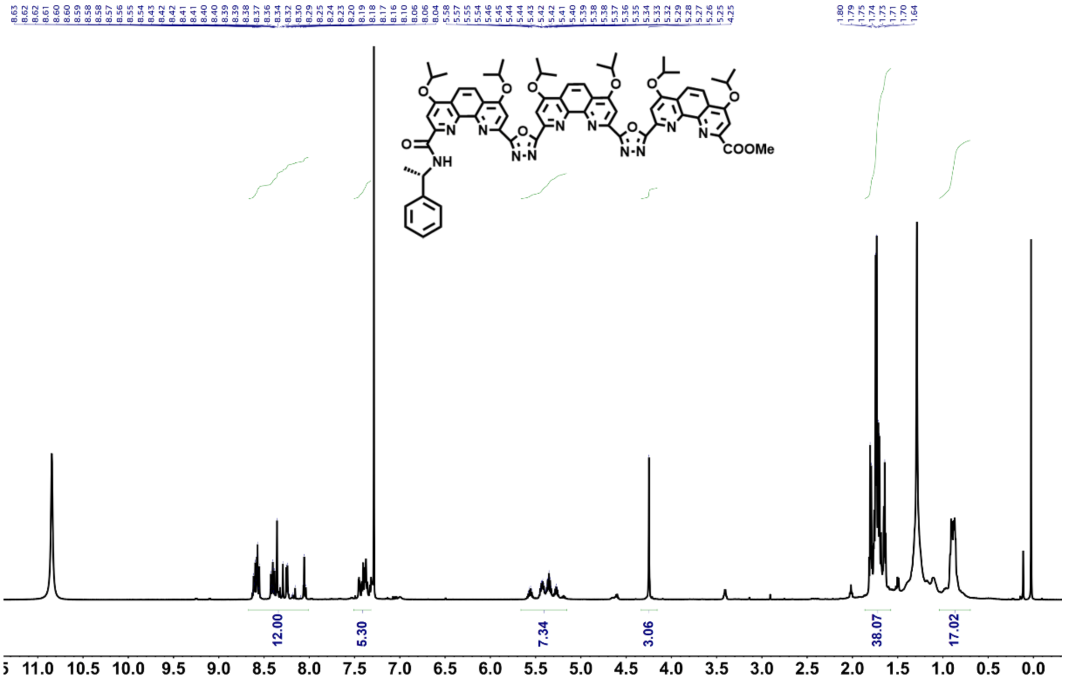

Figure S25. ^{1}H NMR spectrum of **HM8** in $CDCl_3$ ($CDCl_3$/Trifluoroacetic acid-D 50:1).

ESI-full spectrum

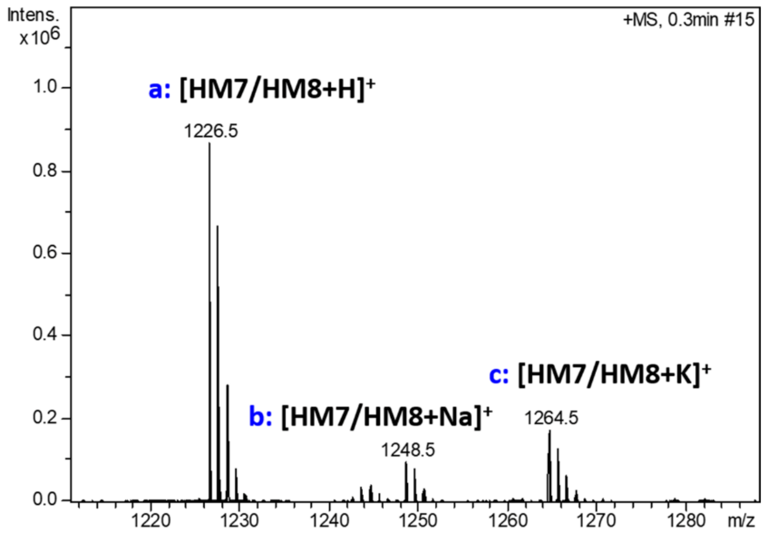

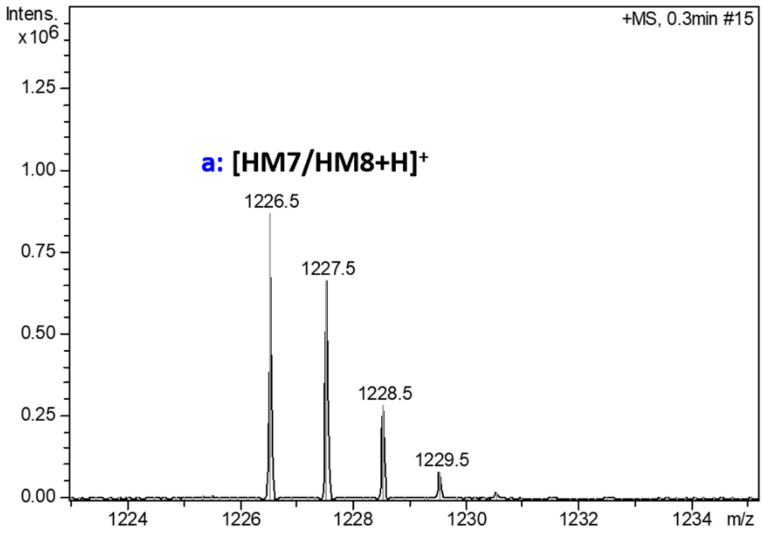

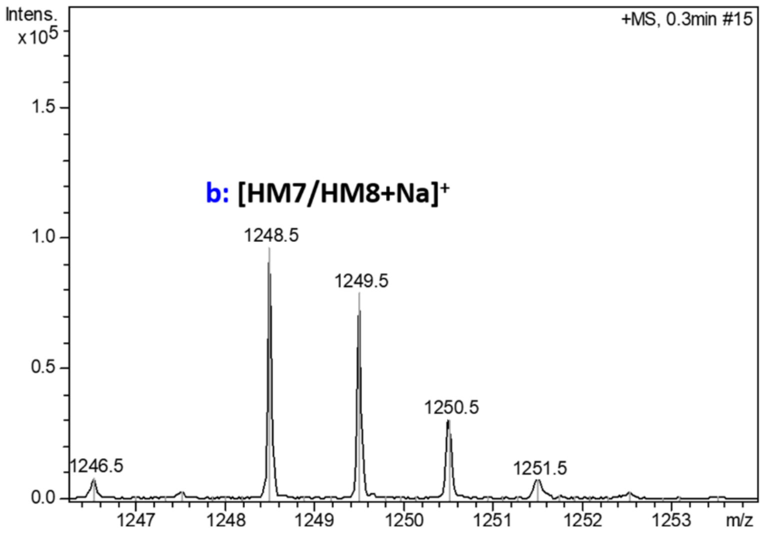

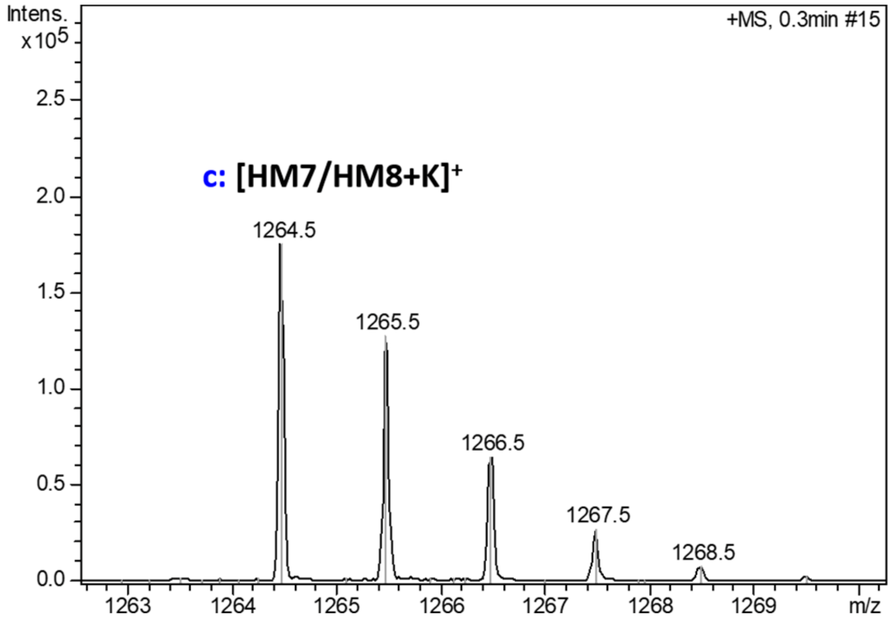

Figure S26. ESI MS spectrum of **HM7** and **HM8**.

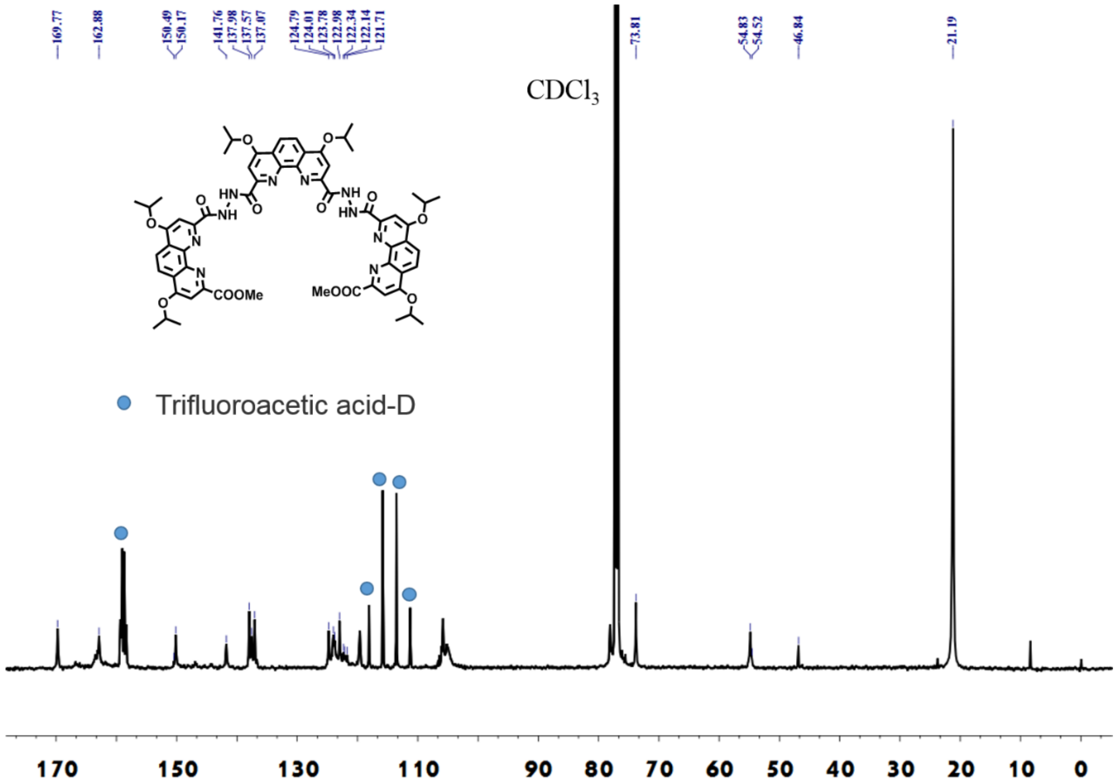

Figure S27. ^{13}C NMR spectrum of compound 19 in $CDCl_3$ ($CDCl_3$/ Trifluoroacetic acid-D 50:1).

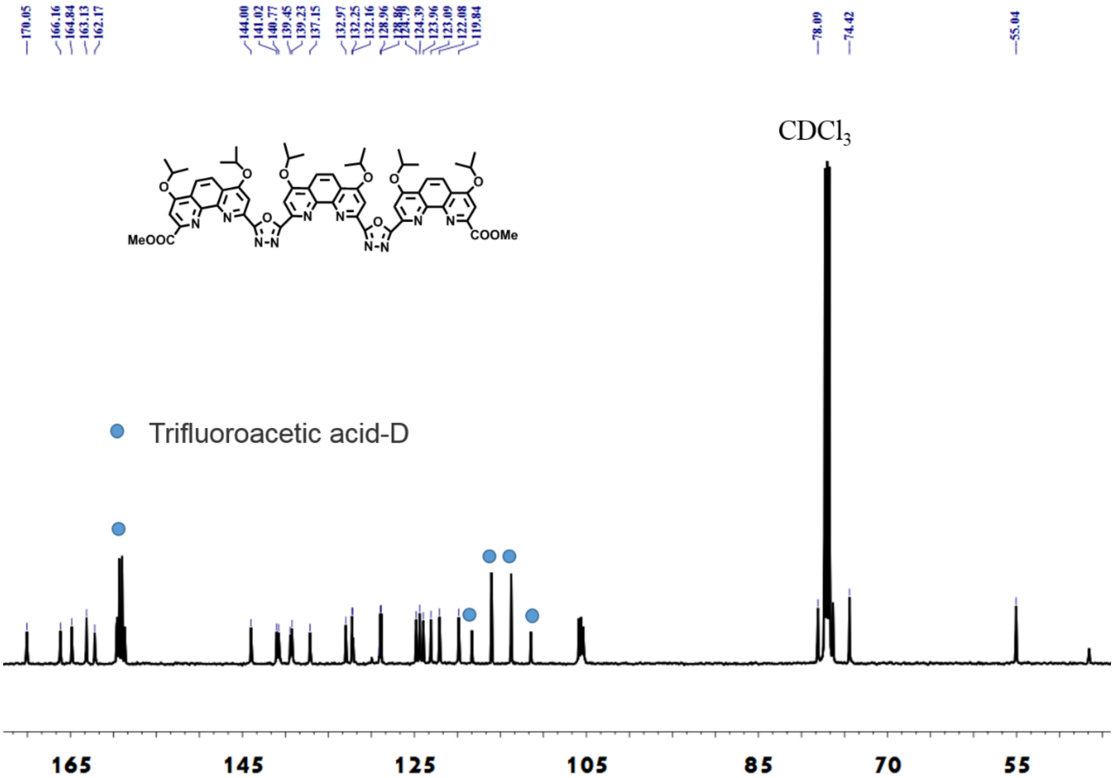

Figure S28. ^{13}C NMR spectrum of compound 20 in $CDCl_3$ ($CDCl_3$/ Trifluoroacetic acid-D 50:1).

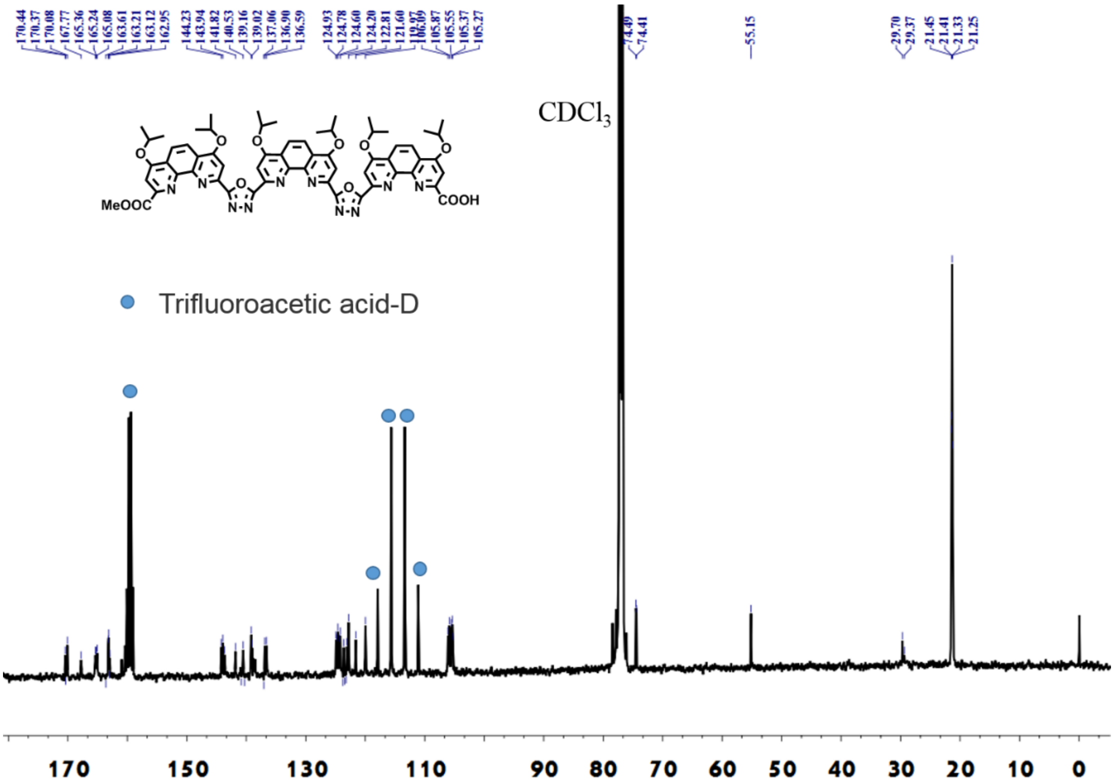

Figure S29. ^{13}C NMR spectrum of compound 9 in $CDCl_3$ ($CDCl_3$/ Trifluoroacetic acid-D 50:1).

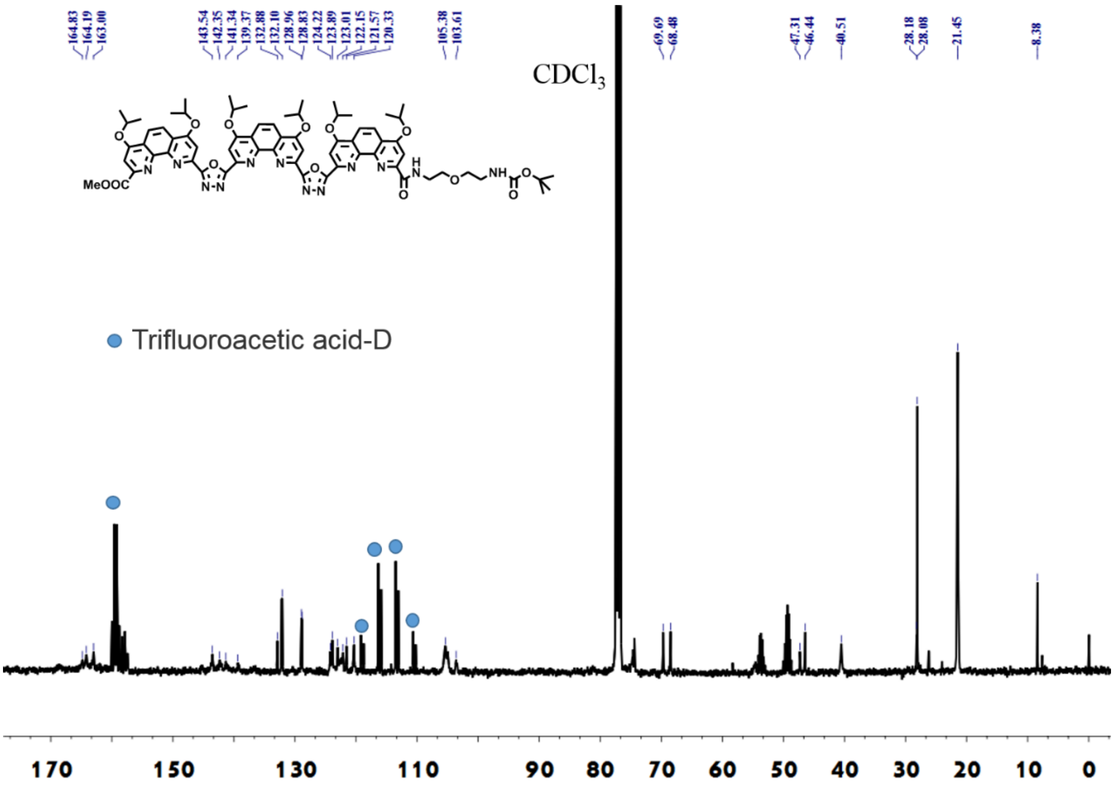

Figure S30. ^{13}C NMR spectrum of compound 6 in $CDCl_3$ ($CDCl_3$/ Trifluoroacetic acid-D 50:1).

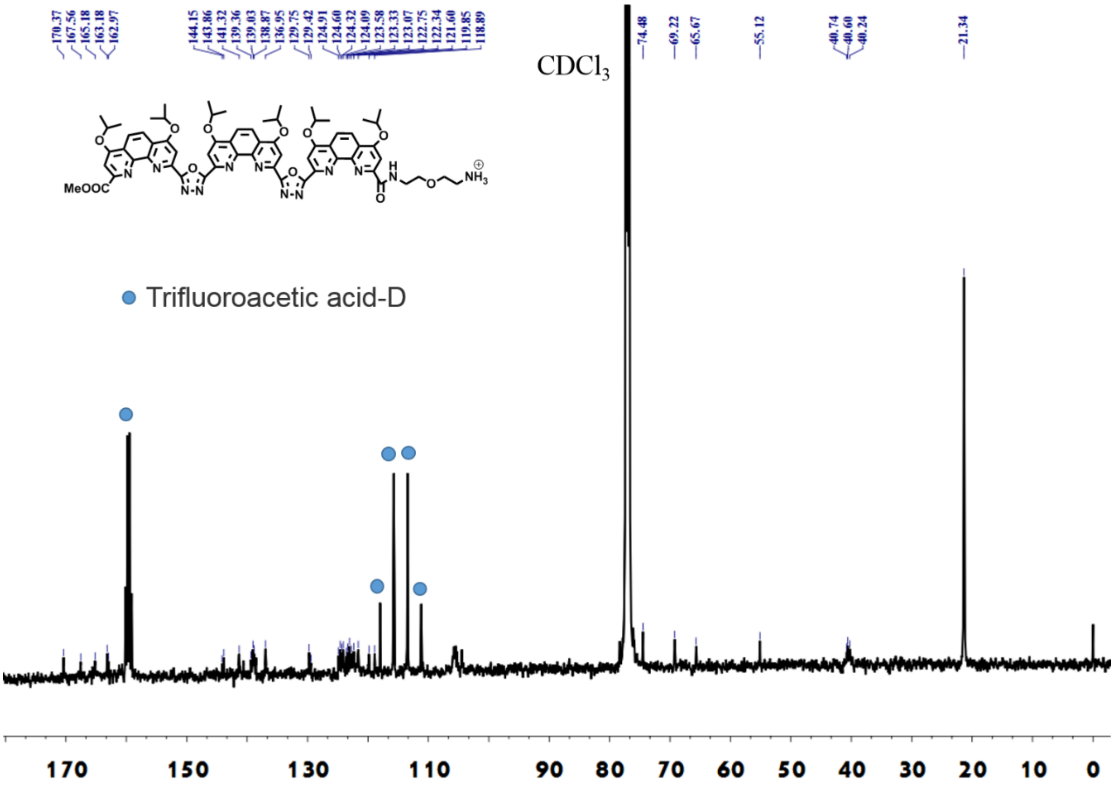

Figure S31. ^{13}C NMR spectrum of compound 21 in $CDCl_3$ ($CDCl_3$/ Trifluoroacetic

acid-D 50:1).

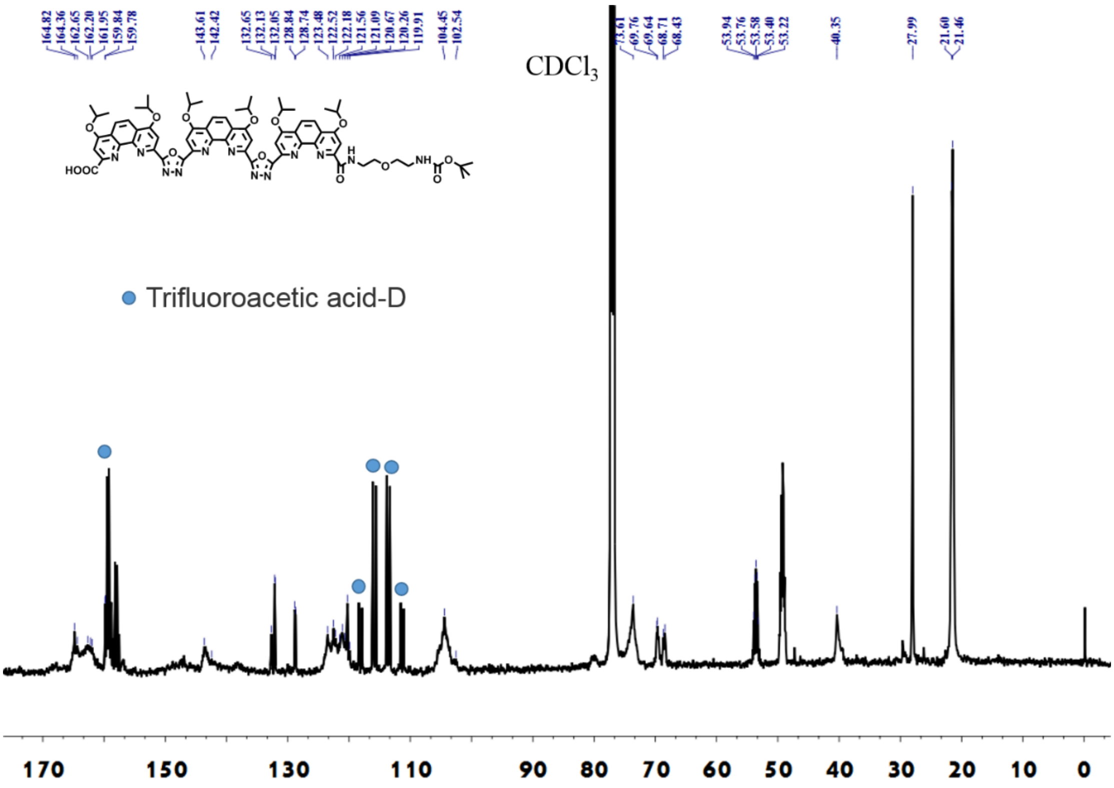

Figure S32. ^{13}C NMR spectrum of compound 22 in $CDCl_3$ ($CDCl_3$/ Trifluoroacetic acid-D 50:1).

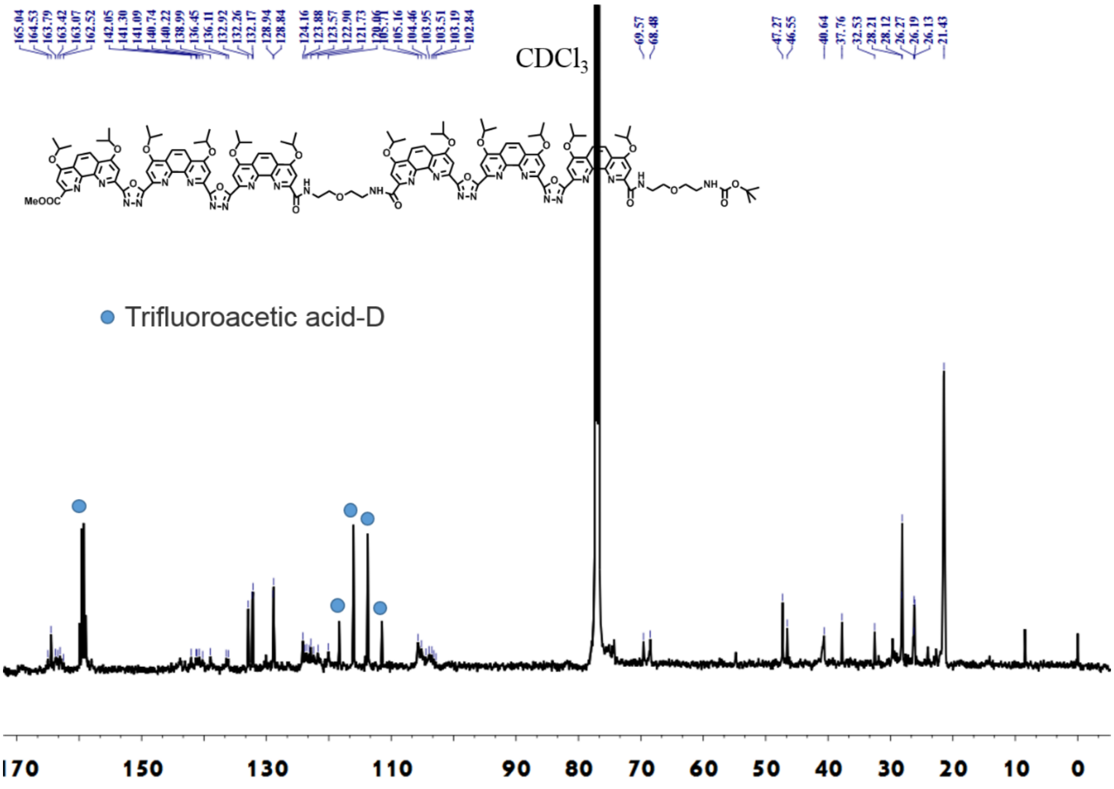

Figure S33. ^{13}C NMR spectrum of HM1 in $CDCl_3$ ($CDCl_3$/ Trifluoroacetic acid-D 50:1).

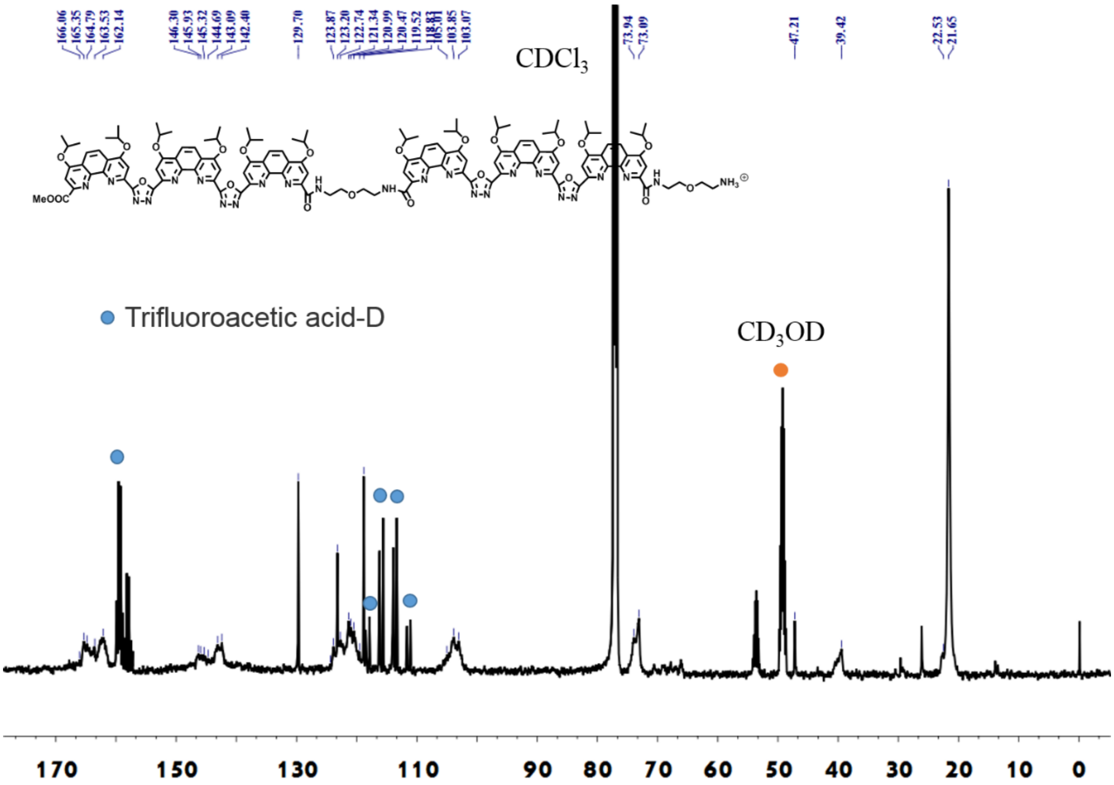

Figure S34. ^{13}C NMR spectrum of HM2 in $CDCl_3$ ($CDCl_3$/ Trifluoroacetic acid-D 50:1).

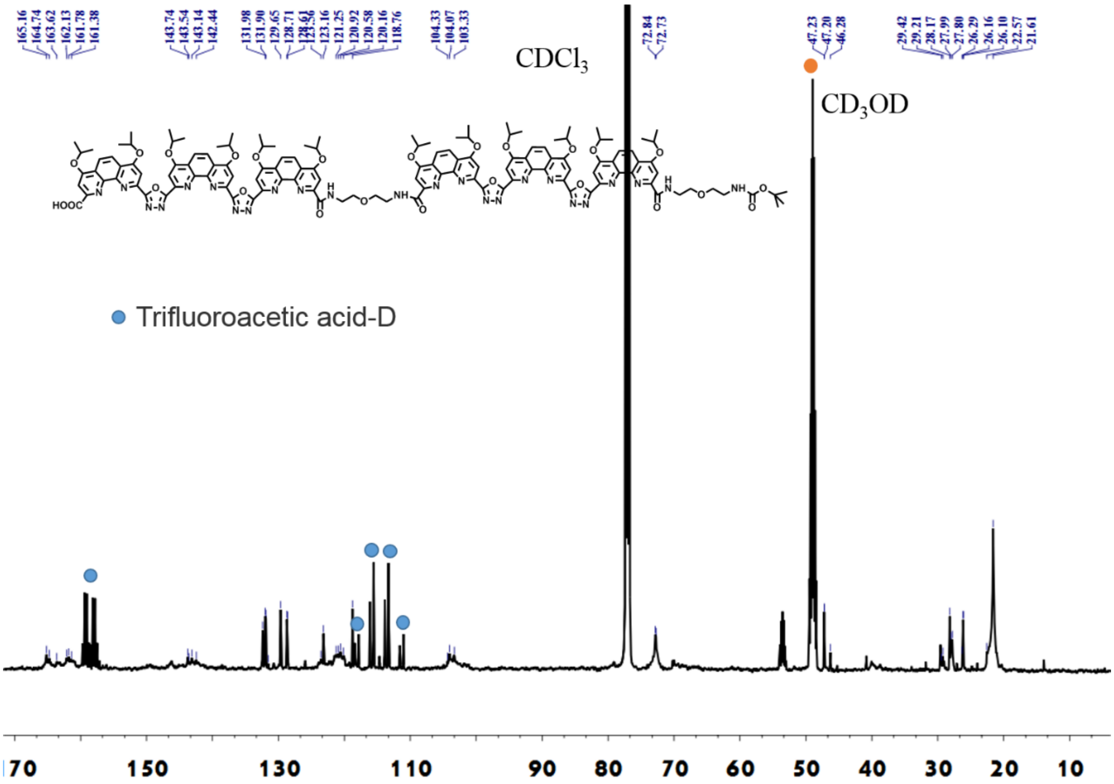

Figure S35. ^{13}C NMR spectrum of HM3 in $CDCl_3$ ($CDCl_3$/ Trifluoroacetic acid-D/CD_3OD 50:1:5).

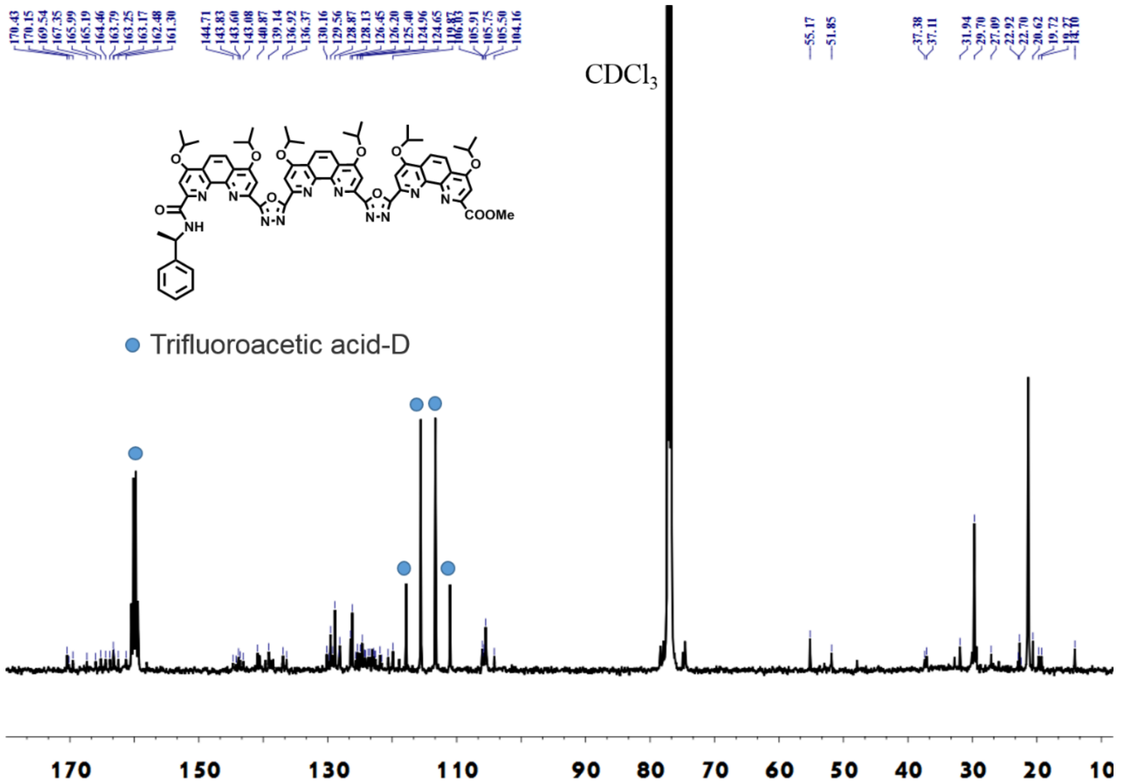

Figure S36. ^{13}C NMR spectrum of HM7(8) in $CDCl_3$ ($CDCl_3$/ Trifluoroacetic acid-D 50:1).

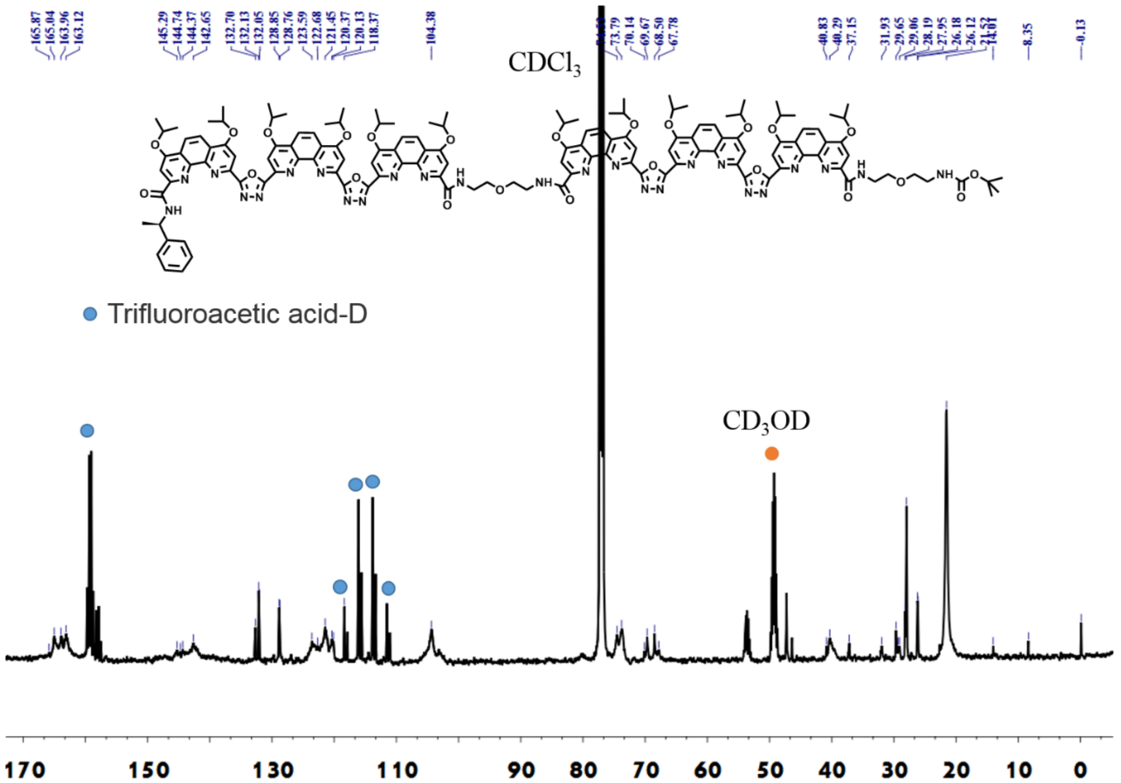

Figure S37. ^{13}C NMR spectrum of HM4(HM5) in $CDCl_3$ ($CDCl_3$/ Trifluoroacetic acid-D/CD_3OD 50:1:1).

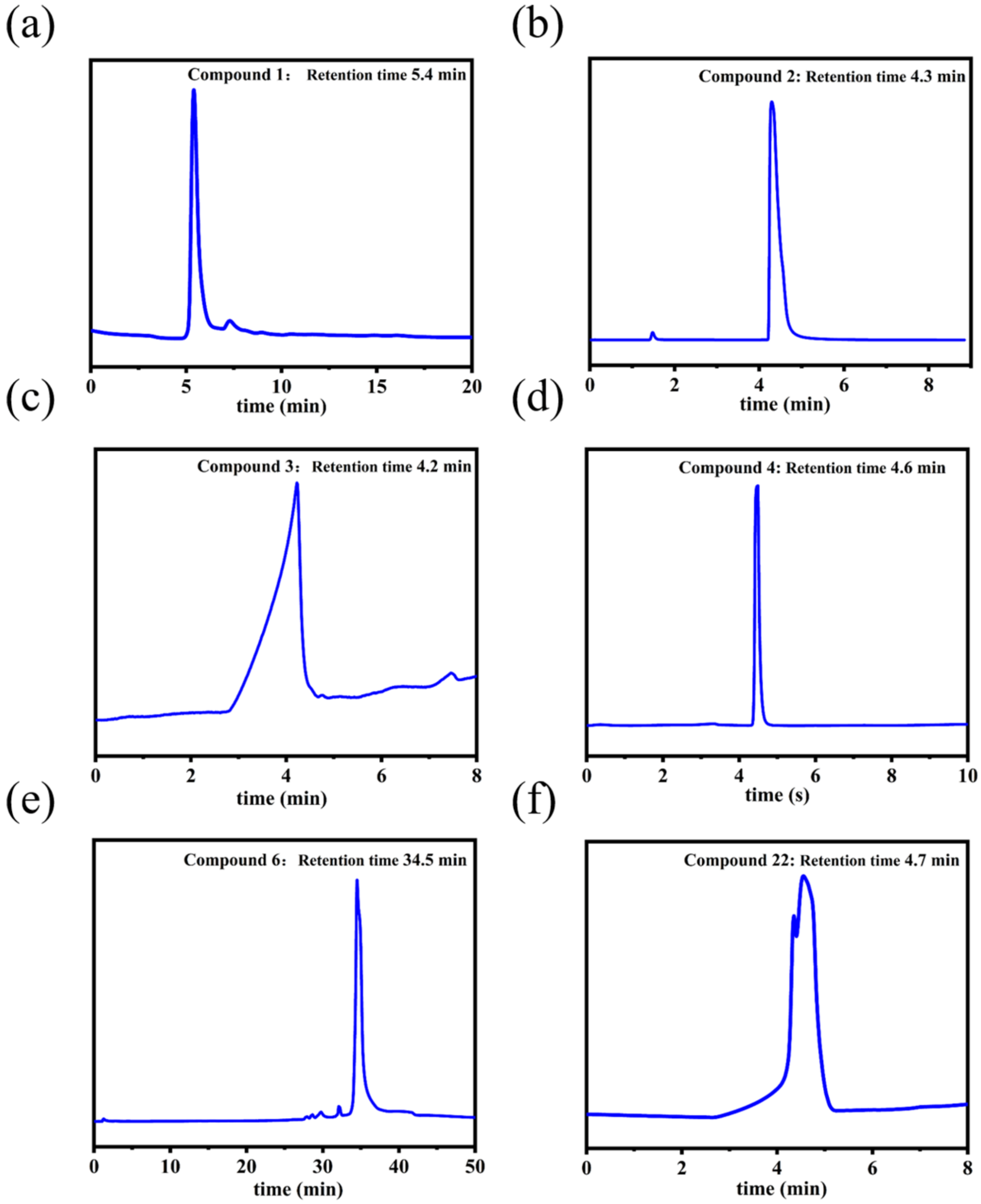

Figure S38. The HPLC chromatograph of compound 1-4, compound 6, compound 22. HPLC analysis with C4 column ((CBM-20A, YMC-Pack Pro C4; Mobile phases: water and acetonitrile; flow rate:0.5mL/min; λ = 365 nm)k

4. The CD spectra of HM1 / HM4 / HM5 / HM7 / HM8 / HM9

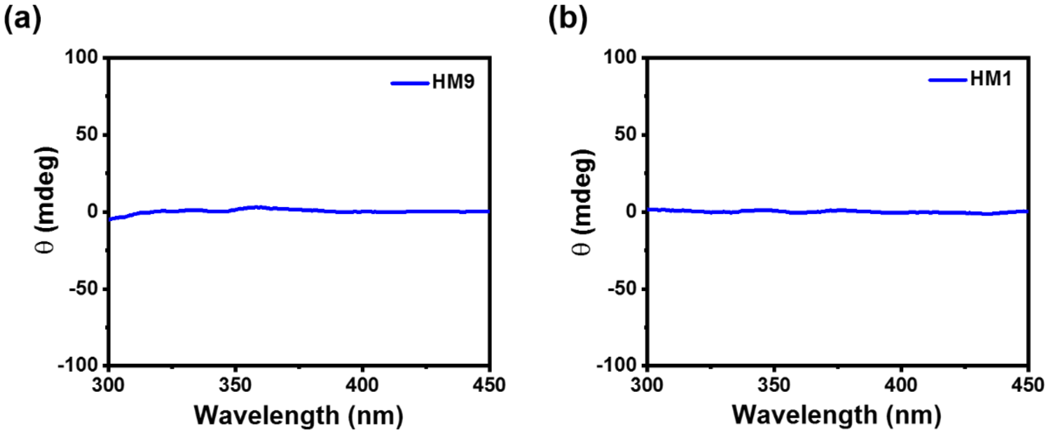

Figure S39. (a) The CD spectra of **HM9** at 25 μM. (b) The CD spectra of **HM1** at 25 μM.

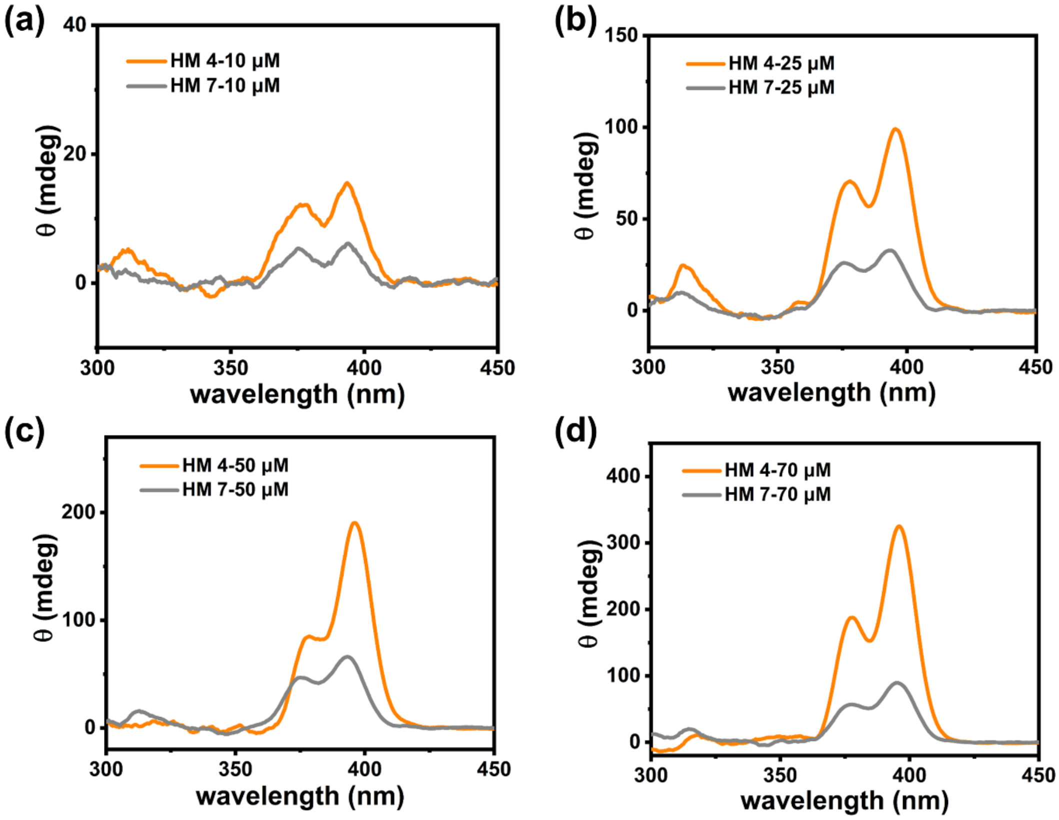

Figure S40. (a) - (d) The comparison of CD intensity by **HM4** and **HM7** in different concentration (10 μM, 25 μM, 50 μM and 70 μM).

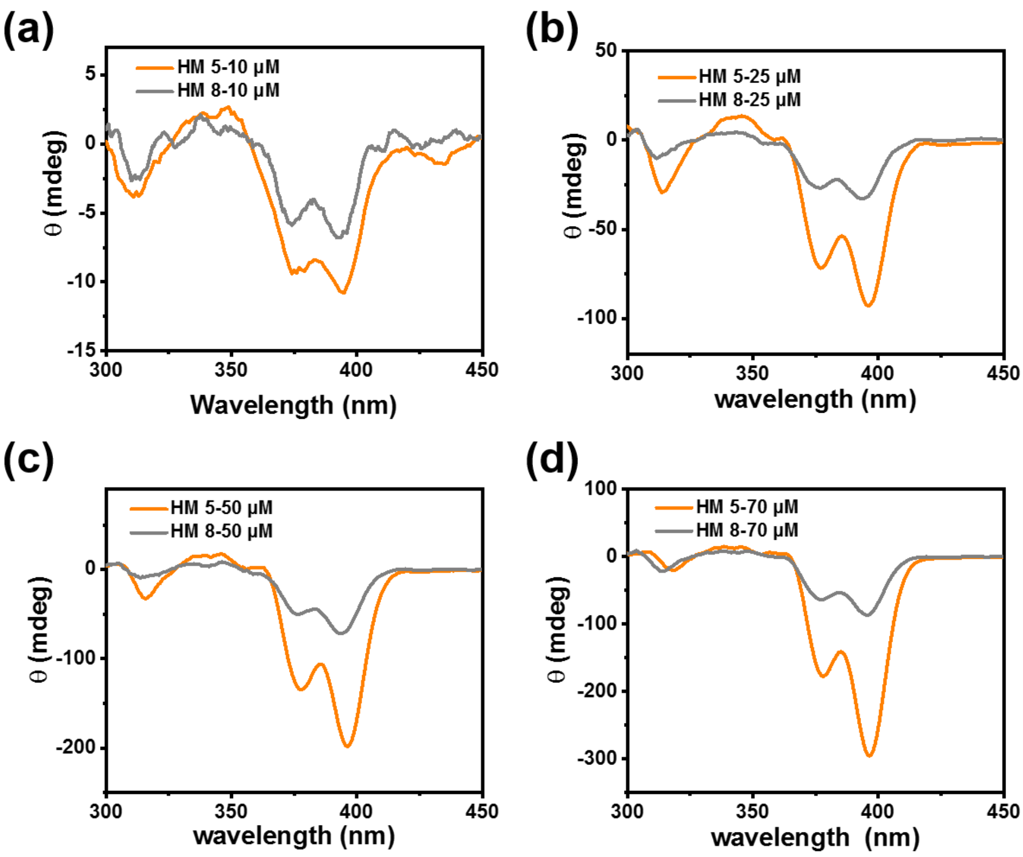

Figure S41. (a) (b) (c) (d) The comparison of CD intensity by **HM5** and **HM8** in different concentration (10 μM, 25 μM, 50 μM and 70μM).

Ration = θ(HM4)/θ(HM7) or Ration = θ(HM5)/θ(HM8)

Concentration (μM)	10	25	50	70
Ration	2.5	2.9	2.8	3.5

Table S1. The ration of the CD intensity of **HM5** and **HM8** in different concentration (10 μM, 25 μM, 50 μM and 70 μM) at the wavelength of 395 nm.

5. Fluorescence spectra of HM1 / HM3

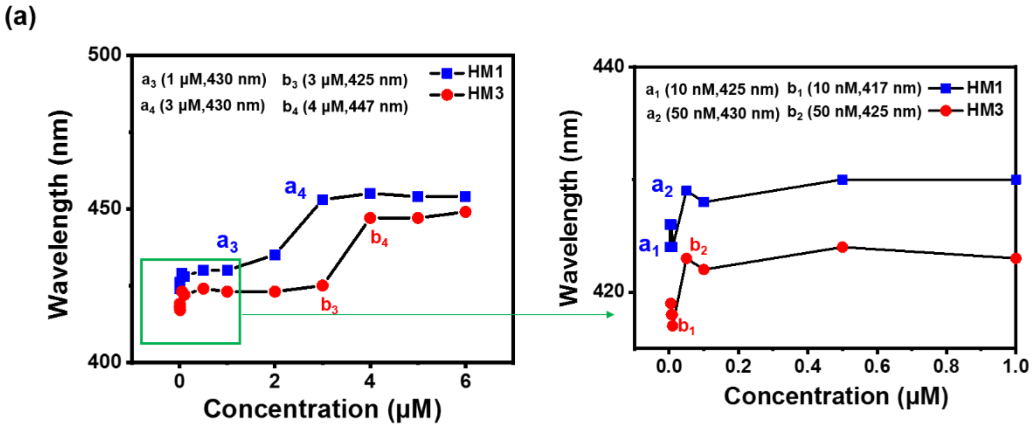

Figure S42. (a) Quantified analysis of the relationship between the maximum emission wavelength and the molar concentration of **HM1** and **HM3.**

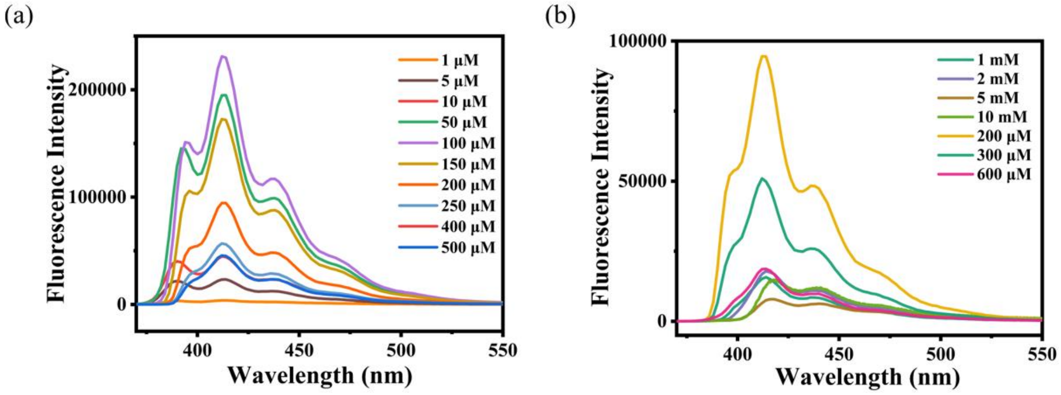

Figure S43. Fluorescence titration of ophenanthroline-oxadiazole-based trimer (excitation wavelength: 370 nm). (a) Molecule concentration between 1 μM and 500 μM. (b) Molecule concentration between 600 μM and 10 mM.

6. The UV spectra of HM1 / HM3 / HM4

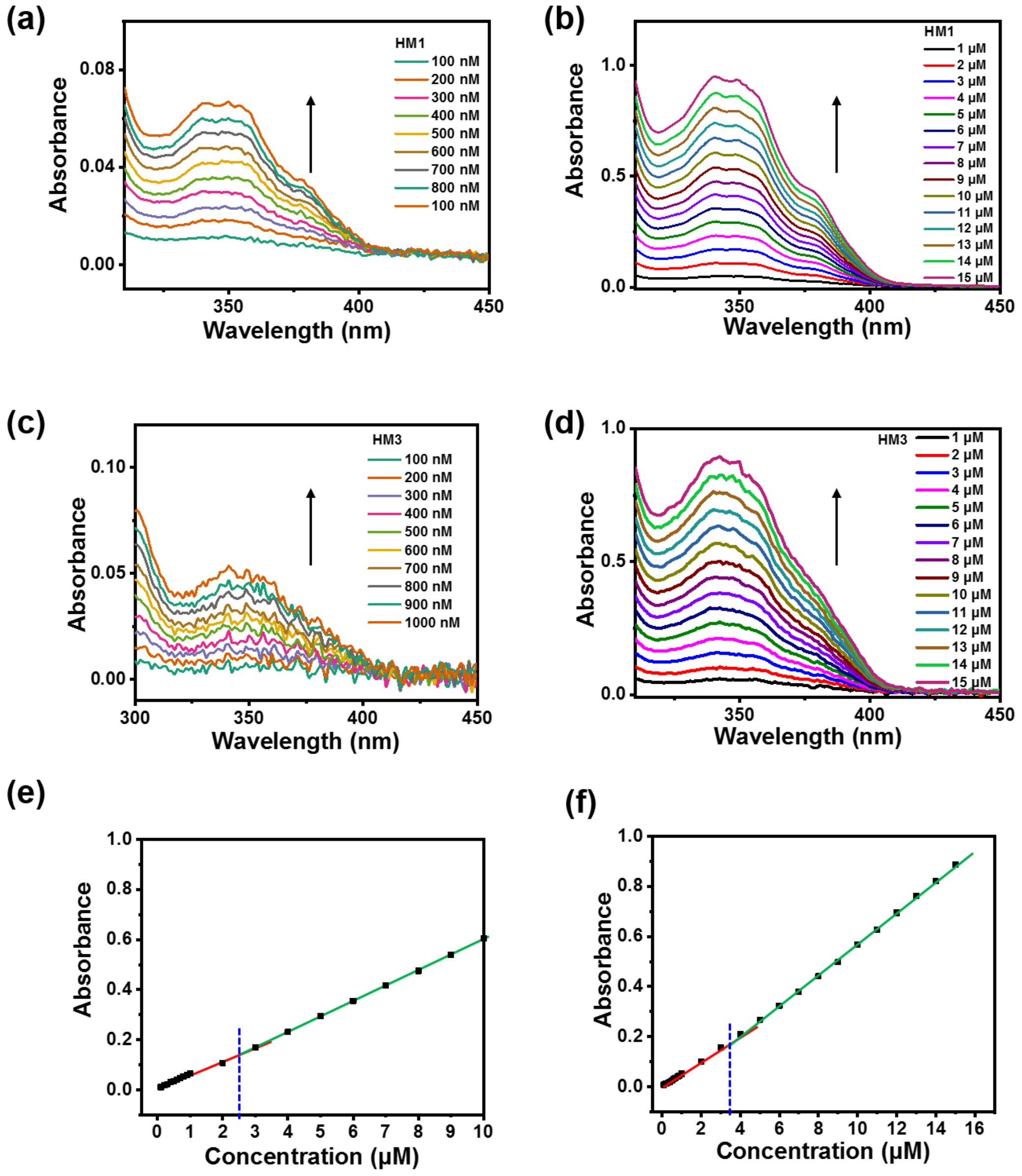

Figure S44. (a) (b) The ultraviolet-visible (UV-Vis) spectra of **HM1** in $CHCl_3$ at 298K at different concentrations. (c) (d) The ultraviolet-visible (UV-Vis) spectra of **HM3** in $CHCl_3$ at 298K at different concentrations. (e) Plots of absorbance at 370 nm versus concentrations in relation to **HM1**. (f) Plots of absorbance at 370 nm versus concentrations in relation to **HM3**.

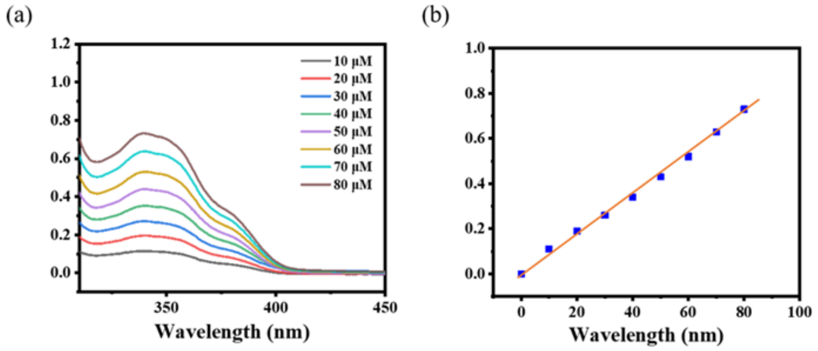

Figure S45. (a) The UV-Vis spectra of HM4 in $CHCl_3$ at 298 K at different concentrations (0-7 μM); (b) Plots of absorbance at 340 nm versus concentrations.

7. The assembly of HM1 / HM2 on the silicon wafer surface

The **HM1** and **HM2** were dissolved in acetonitrile which their concentration was varied from 10^{-8} M to 10^{-9} M. Applied 5 μL of the solution to the silicon wafer and evaporated the solvent naturally in sealed surface dish at 45 ℃. To prevent the contamination of the silicon wafers, the silicon wafers with assembled molecules must be conserved in an airtight environment before testing.

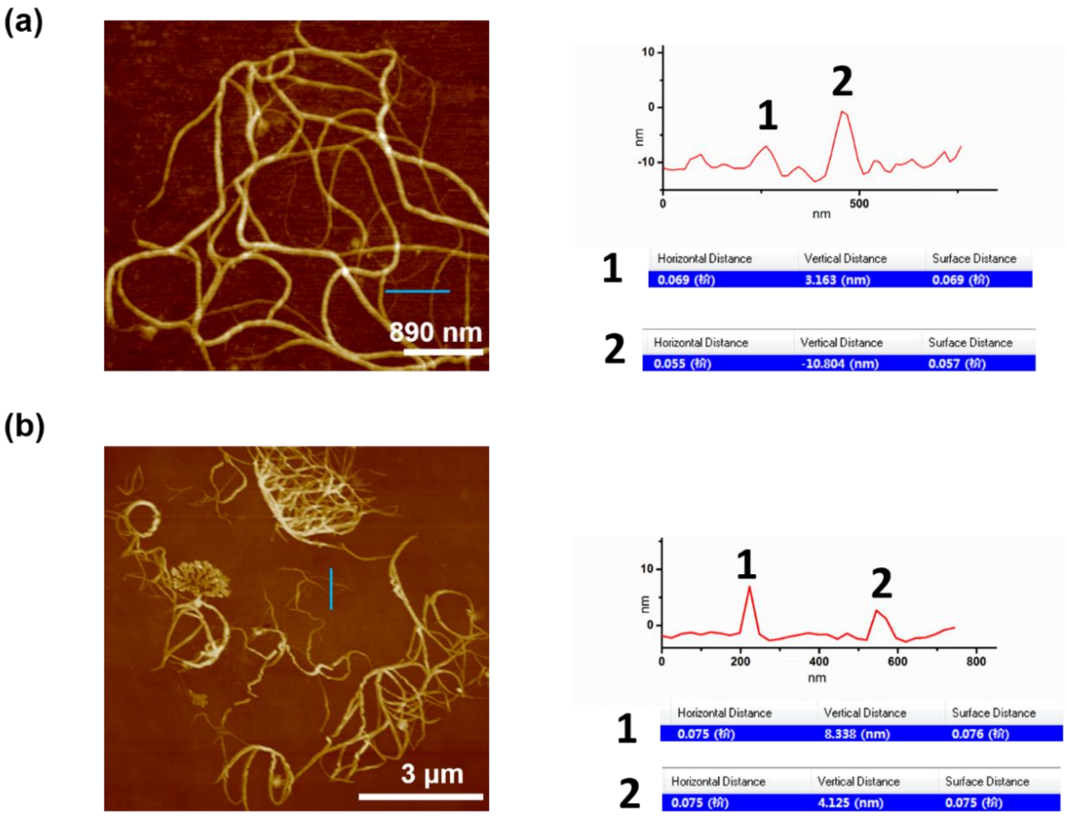

Figure S46. (a) AFM images of the self-assembly of **HM1** in acetonitrile with multiple assembly sizes. (b) AFM images of the self-assembly of **HM2** in acetonitrile with multiple assembly sizes.

8. Fluorescence titration experiments of ion-bounding

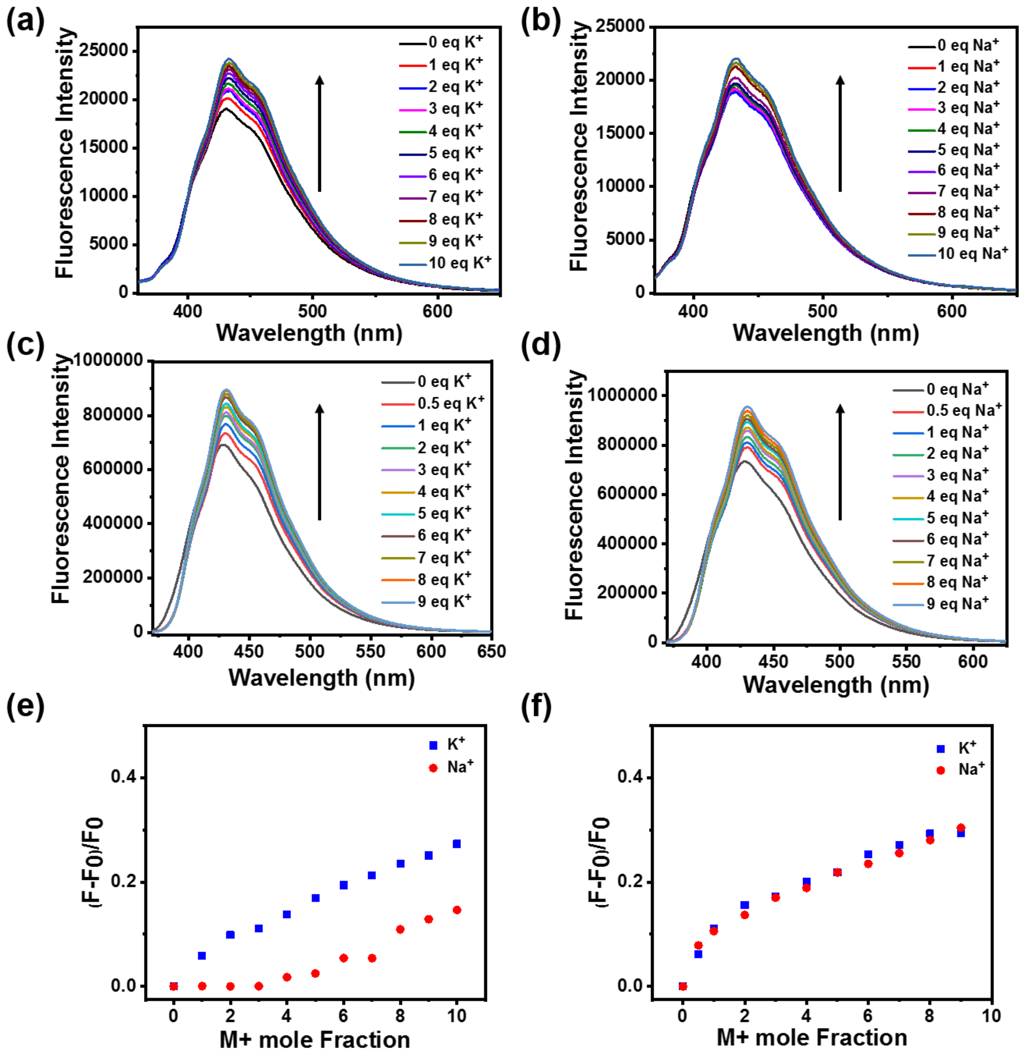

Figure S47. (a), (b) The fluorescence titrations of **HM1** for K^+ and Na^+ at the concentration of 10 μM in DMSO upon adding 10 eq the corresponding ions. (c) (d) The fluorescence titrations of **HM2** for K^+ and Na^+ at the concentration of 10 μM in the mixture solvent acetonitrile/water (9:1, vol/vol) through adding different amounts of corresponding ions. (e) The increments in fluorescence intensity of **HM1**. (f) The increments in fluorescence intensity of **HM2**.

9. Ion transport experiments using HPTS assay

The vesicles were prepared as follows: 50 mg EYPC (egg yolk L-α-phosphatidylcholine) dissolved in 5 mL chloroform and divided them into equally into five clear flat-bottomed vials then the solution was dried under a nitrogen atmosphere after that the phospholipids are kept under vacuum for 12 hours. Keep the drained phospholipids sealed in the fridge at -20°C for auxiliary. Before using the prepared phospholipids, the vial is naturally warming to room temperature. The phospholipid was hydrated in HEPES (1 mL, 10 mM HEPES 100 mM NaCl/Na_2SO_4/K_2SO_4, pH = 7.0) buffer solution with 1 mM HPTS for 3 hours in a 37°C thermostat. The suspension was subjected to ten freeze-thaw cycles through liquid nitrogen and 40°C thermostat water-bath. The mixture was extruded through 200 μm polycarbonate membrane for ten times, then the mixture was purified by Sephadex G-50 to remove the dye outside the vesicles with HEPES buffer mobile phase.

The HPTS-containing LUV suspension 50 μL (the vesicle contains 100 mM NaCl at pH = 7.0) was added into 950 μL HEPES buffer solution (10 mM HEPES, 100 mM NaCl/Na_2SO_4/K_2SO_4 pH = 7.8) to generate a pH gradient for ion transport study. The channel was dissolved in DMSO, 10 μL channel is added to the test system and stirred. Fluorescence intensity (E_t) was consecutively monitored at 510 nm (excitation 460 nm, emission of HPTS at 510 nm) at the moment of addition of the channel molecules. The monitoring of fluorescence changes was terminated at the moment of the addition of 20% Triton X-100 solution. The fluorescence data was normalized according to the equation:

$$R_f = (E_t - E_0)/(E_\infty - E_0)$$

R_f: normalized fluorescence intensity.

E_0: the initial emission intensity.

E_∞: the final emission intensity.

Y was considered as transmembrane transport activity.

The Hill coefficient n and effective concentration EC50 can be obtained by the Hill equation:

$$Y = Y_\infty + (Y_0 - Y_\infty)/(1 + (c/EC50)^n)$$

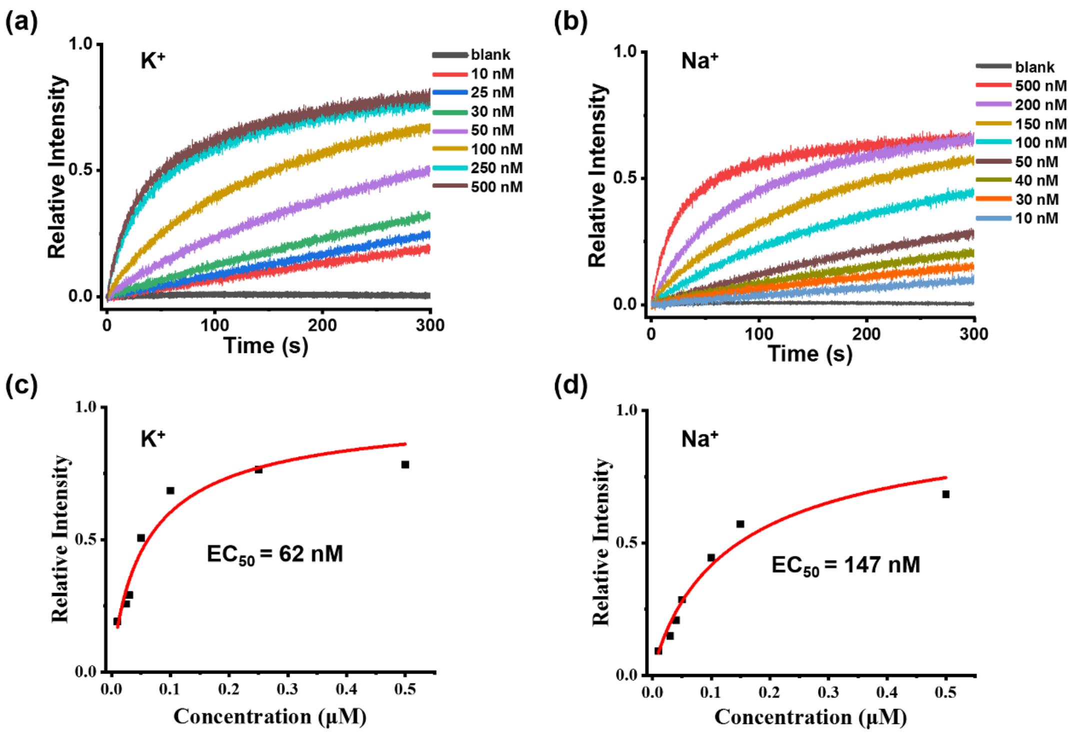

Figure S48. (a) K^+ and (b) Na^+ transport activity of **HM2** at different concentration (external-vesicle KCl buffer pH = 7.8). (c) K^+ and Na^+ Hill plot for transport by **HM2** (external-vesicle KCl buffer pH = 7.8).

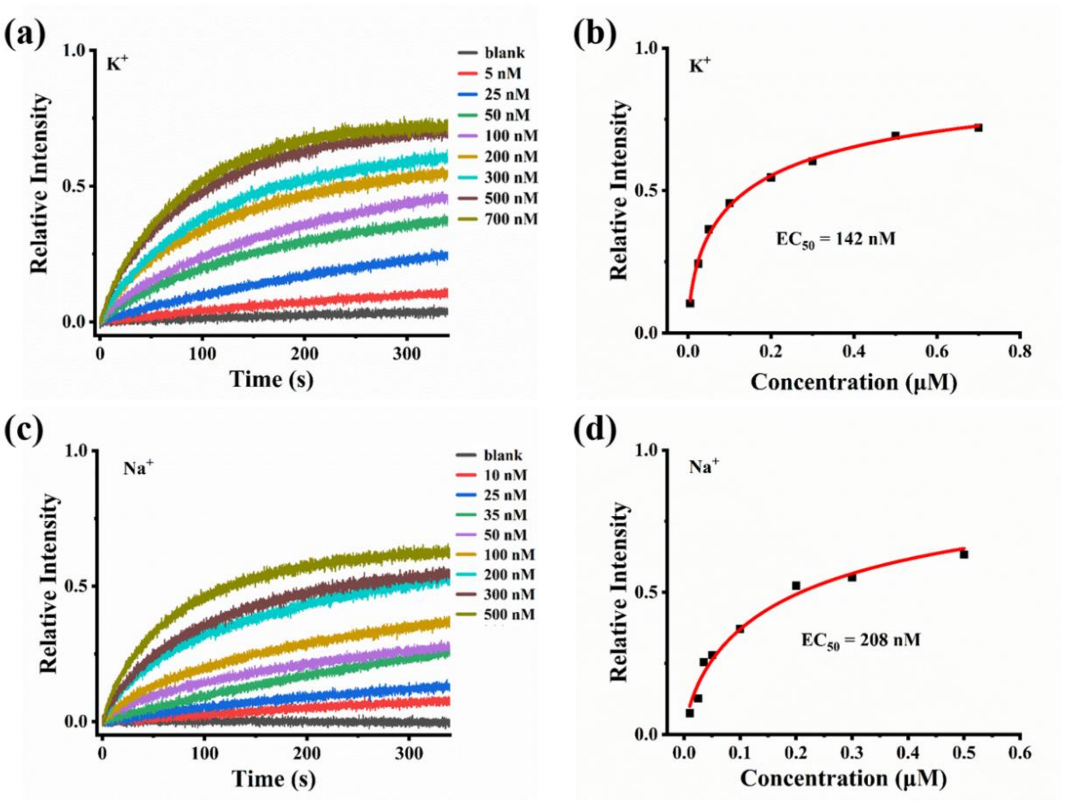

Figure S49. (a) K^+ transport activity of **HM3** at different concentration. (b) K^+ Hill plot for transport by **HM3**. (c) Na^+ transport activity of **HM3** at different concentration (d) Na^+ Hill plot for transport by **HM3**.

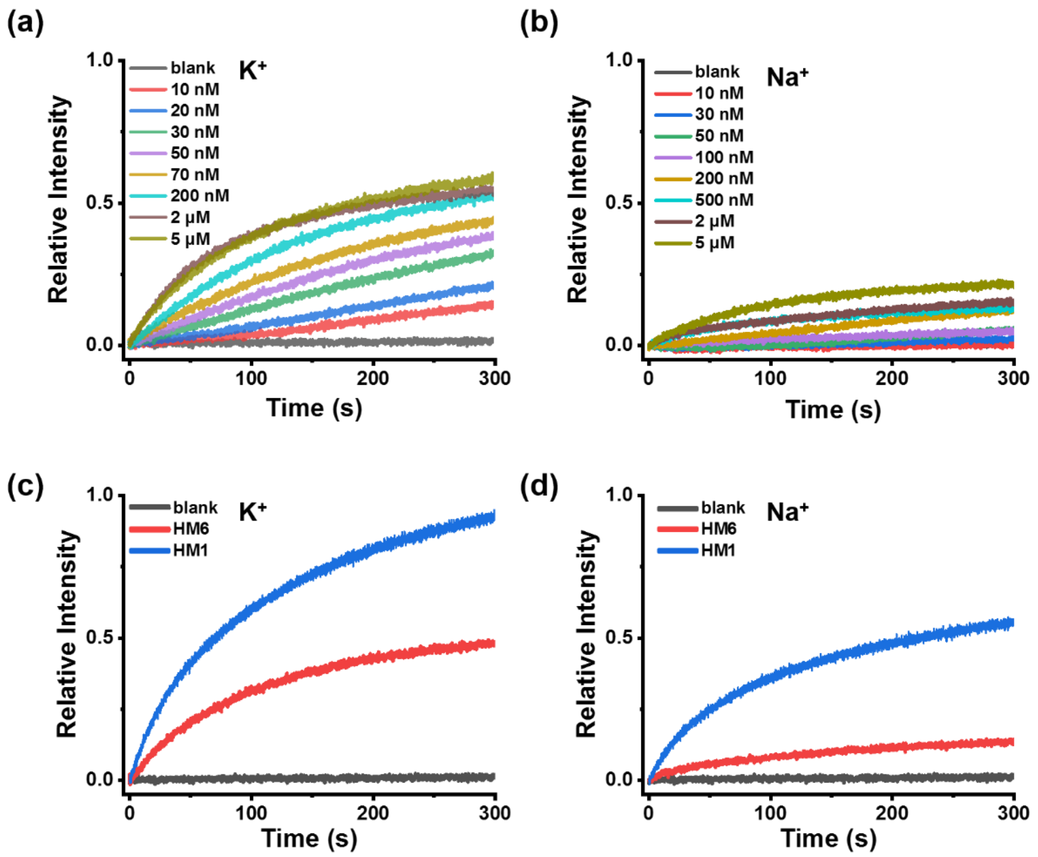

Figure S50. (a) The transport activity for K^+ of **HM6** at different concentration. (b) The transport activity for Na^+ of **HM6** at different concentration. (c) The transport activity for K^+ of H**M6** and **HM1** at 1 mM. (d) The transport activity for Na^+ of **HM6** and **HM1** at 1 μM.

10. The inhibition for K^+ transport by Lys of HM1

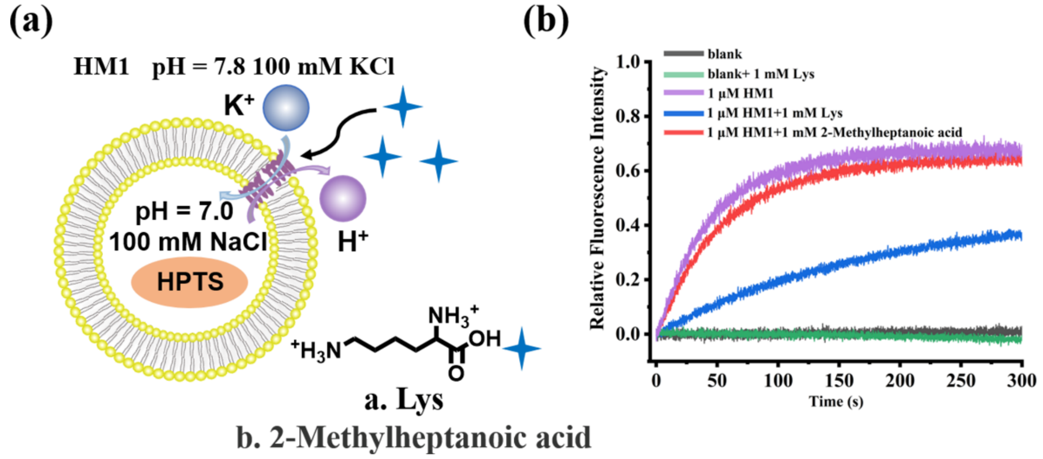

Figure S51. (a) Schematic illustration for the transport inhibition experiment by addition of Lys; (b) The inhibition to the K^+ transport of **HM1** through the addition of 1 mM Lysine **(Lys)**.

11. Continuous regulation of the pH-responsive behaviour for potassium of HM2

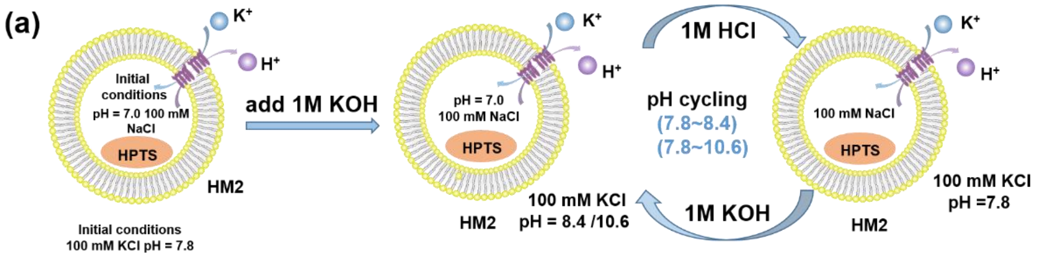

Figure S52. (a) The schematic representation of liposomes with continuous dynamic regulation for K^+ about **HM2**.

12. Proton transfer experiment

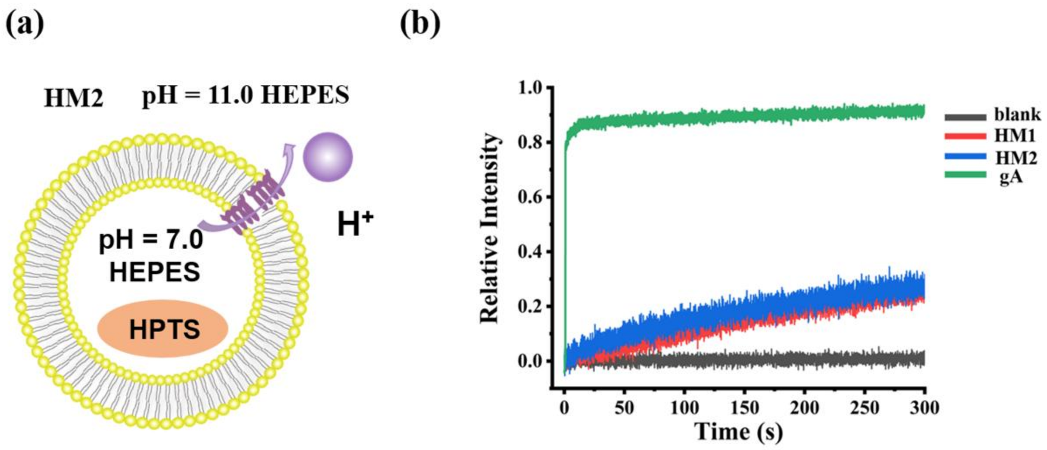

Figure S53. (a) The liposomal diagram for proton transport by **HM2** in a discharged state; (b) The proton transport activities of **HM1**, **HM2**, and **gA** at the concentration of 500 nM.

13. The selectivity ($S_{K/Na}$) of HM1 / HM2 / HM6

The selectivity of K^+ and Na^+ (S_{K^+/Na^+}) was calculated through vesicle transport experiment according to first-order rate equation.

The first-order rate equation:

y = 1-e^(-k*x)

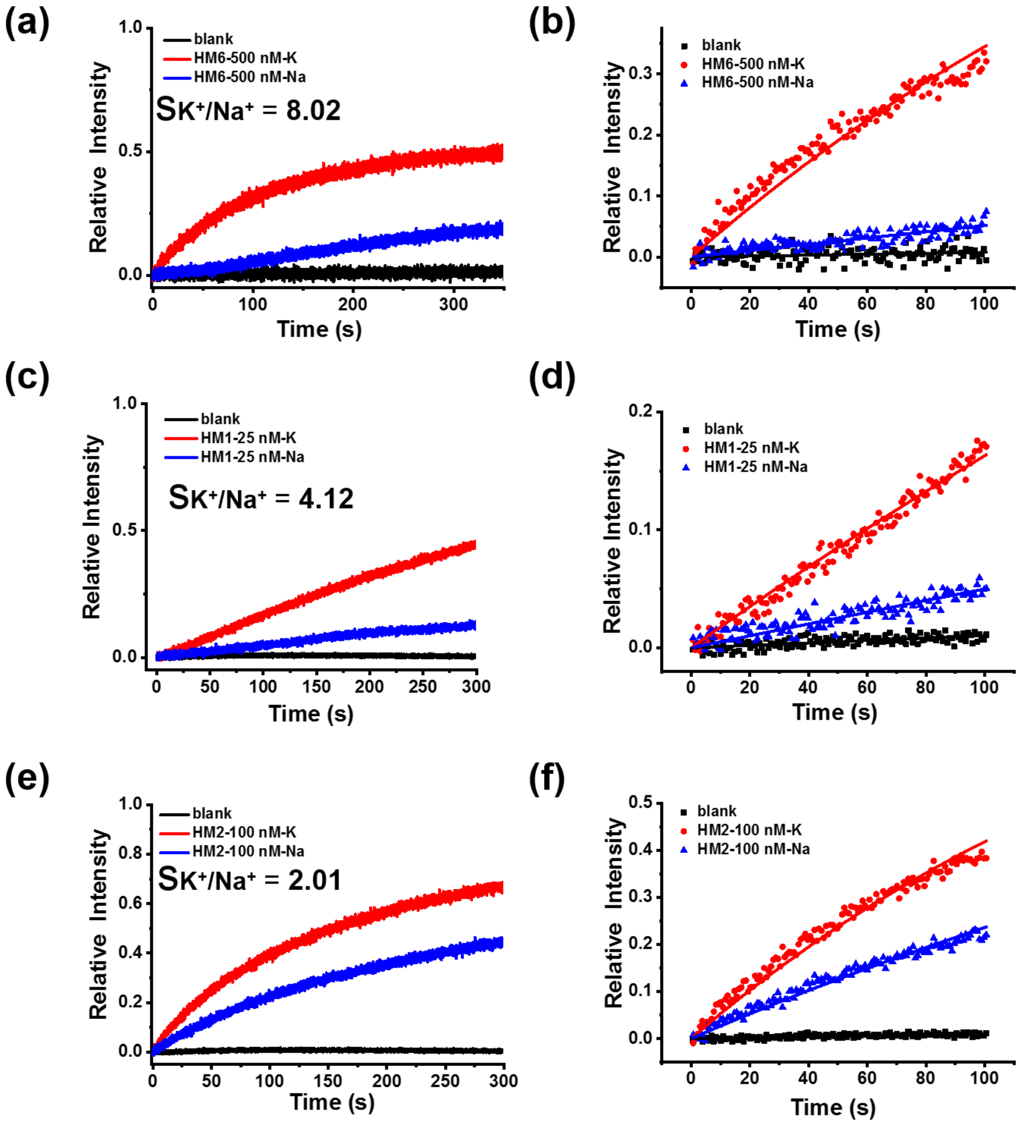

Figure S54. (a) K^+ and Na^+ transport activity of **HM6** at 500 nM. (b) Fitting of normalized fluorescence intensity of **HM6** at the concentration of 500 nM. (c) The relative fluorescence intensity of **HM1** at the concentration of 25 nM. (d) Fitting of normalized fluorescence intensity of **HM1** at the concentration of 25 nM. (e) K^+ and Na^+ transport activity of **HM2** at 100 nM. (f) Fitting of normalized fluorescence intensity of **HM2** at the concentration of 100 nM.

14. Planar lipid bilayer conductance experiment

The DiPhyPC was dissolved in $CHCl_3$ and dried under nitrogen atmosphere. Before testing, the n-decane was added into the vials with the concentration of 25 mg/mL. 0.2-0.3 μL of hexane solution containing phospholipid was pre-coated on the pore with the diameter of 200 μm in the Delrin cup. While the capacitance value is situated between 80 pF and 120 pF, which is conducive for molecules to be embedded into the film. To obtain single-channel signals of **HM1** and **HM2** to potassium ions, the 1 mL 1 M KCl or 1 M NH_4Cl was filled in both the cup (*trans*) and chambers (*cis*). The Ag-AgCl electrodes were inserted vertically into the solution of chambers. Then the 1 μL 100 μM DMSO solution of **HM1** or **HM2** was added into the cup and stir the solution so as to increase the probability of channel embedding in the phospholipid membrane. It is imperative to adjust the voltage on both sides of the membrane to obtain a current signal with a series of voltage.

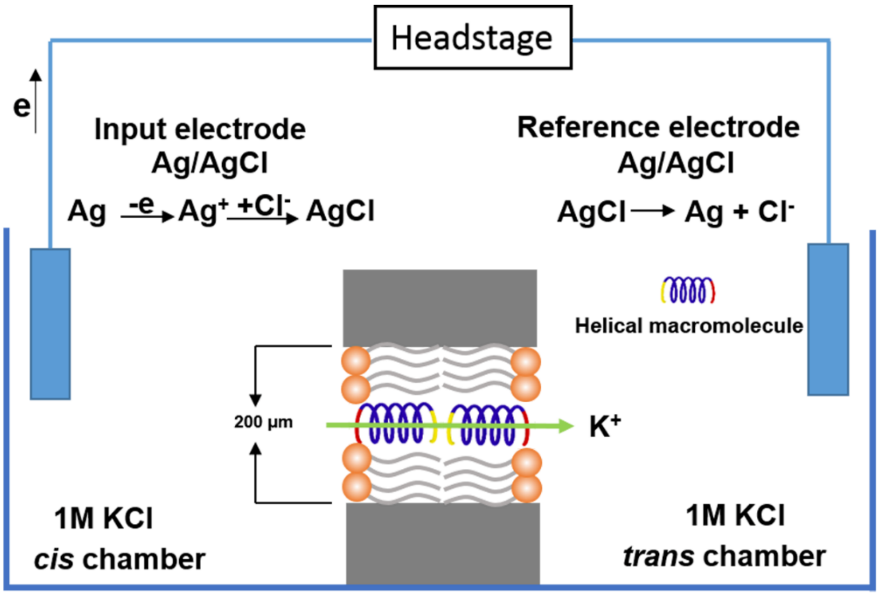

Figure S55. Schematic diagram for the patch clamp experiments with planar lipid bilayer.

14-1. Single-channel conduction current of **HM1** for K^+ (the supplementation of data for lipid bilayers). We captured some signals from **multiple channels** simultaneously embedded in phospholipid membranes fortunately we capture the longer open time of **HM1** and **HM2**.

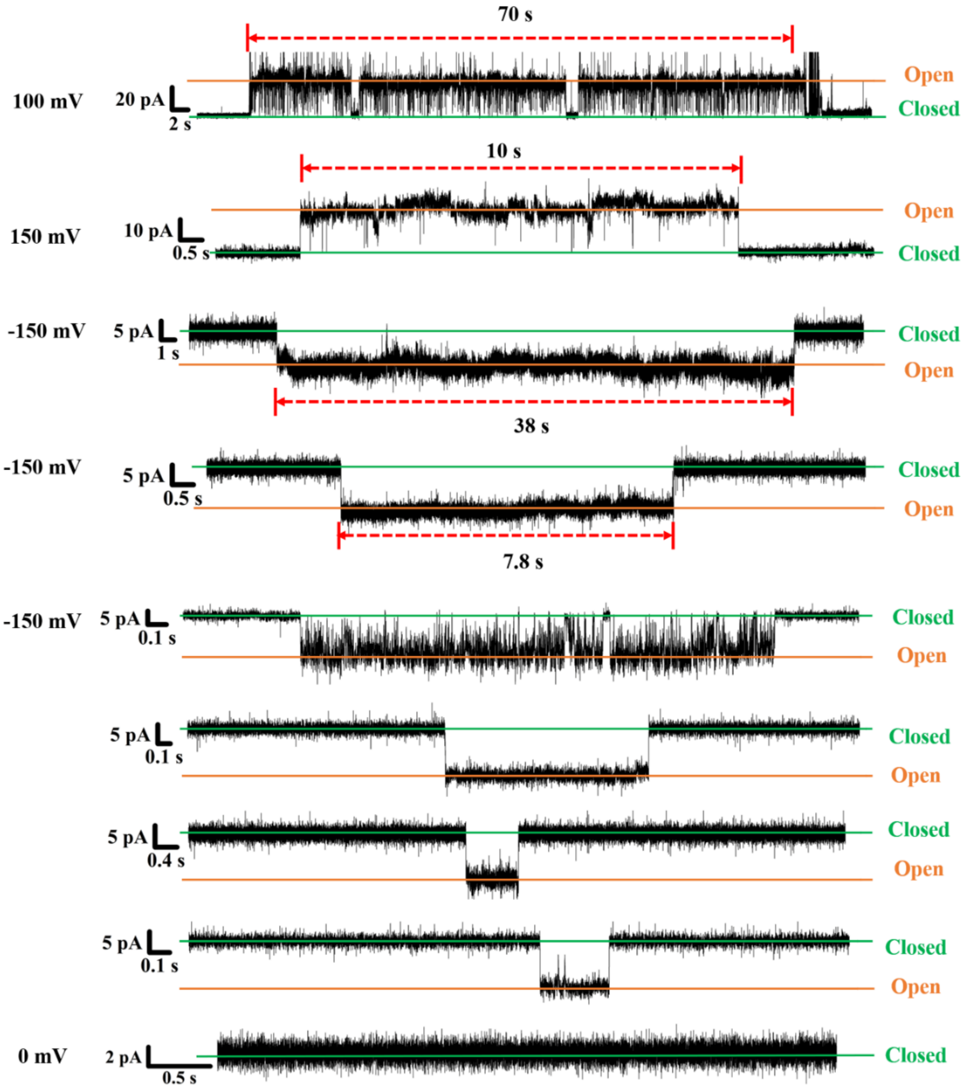

Figure S56. Single-channel current traces of **HM1** recorded at different voltages (*trans* chamber = 1 M KCl, *cis* chamber = 1 M KCl).

14-2. Single-channel conduction current of **HM2** for K^+. We captured some signals from **multiple channels** simultaneously embedded in phospholipid membranes.

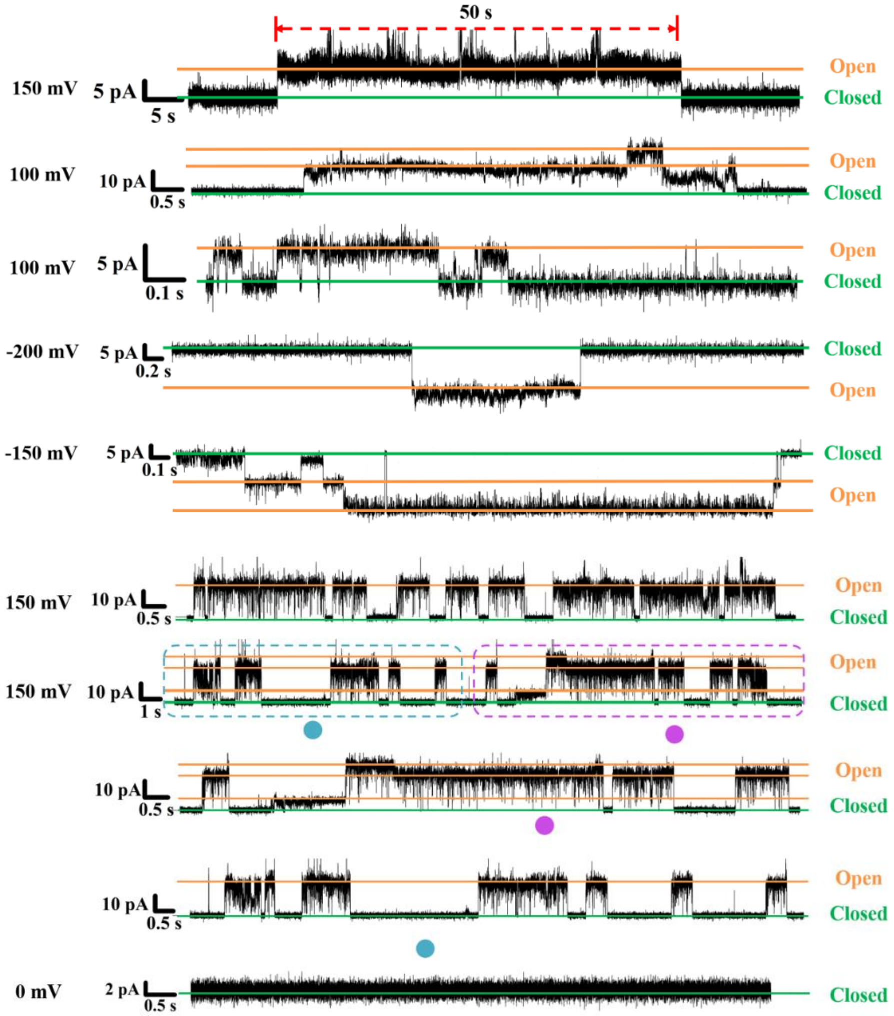

Figure S57. Single-channel current traces of **HM2** for K^+ recorded at different voltages (*trans* chamber = 1 M KCl, *cis* chamber = 1 M KCl).

14-3. Asymmetric BLM experiments of HM1.

15. Supplementary image

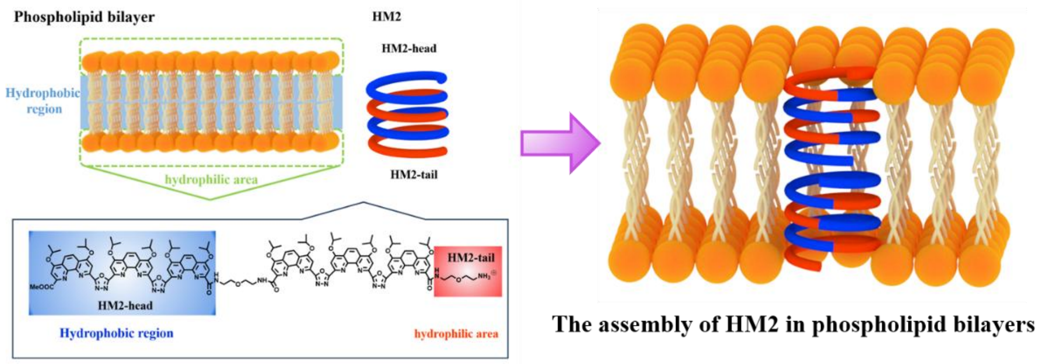

Figure S58. Preferential assembly mode of molecules on membranes.

Reference

S1. Qi, S.; Zhang, C.; Yu, H.; Zhang, J.; Yan, T.; Lin, Z.; Yang, B.; Dong, Z., Foldamer-Based Potassium Channels with High Ion Selectivity and Transport Activity. *Journal of the American Chemical Society* **2021,** *143* (9), 3284-3288.
